# Biphasic effects on human atrial arrhythmogenicity of L-type calcium channel mutations associated with a Brugada/Short QT overlap syndrome - insights from a multiscale simulation study

**DOI:** 10.1371/journal.pcbi.1013616

**Published:** 2025-11-19

**Authors:** Yirong Xiang, Jules C. Hancox, Henggui Zhang

**Affiliations:** 1 Biological Physics Group, Department of Physics and Astronomy, The University of Manchester, Manchester, United Kingdom; 2 Cardiovascular Research Laboratories, Bristol Medical School (THS), Biomedical Sciences Building, University Walk, Bristol, United Kingdom; 3 State Key Laboratory of Digital Medical Engineering, School of Instrument Science and Engineering, Southeast University, Nanjing, China; 4 Key Laboratory of Medical Electrophysiology, Ministry of Education & Medical Electrophysiological Key Laboratory of Sichuan Province, Institute of Cardiovascular Research, Southwest Medical University, Luzhou, China; Lehigh University, UNITED STATES OF AMERICA

## Abstract

Patients with abbreviated cardiac repolarization are at increased risk of cardiac arrhythmias including ventricular and atrial fibrillation (AF). In this computational simulation study, we investigated pro-arrhythmic effects of loss-of-function missense mutations in *CACNA1C* (A39V and G490R Cav1.2) identified in patients with a phenotype combining Brugada syndrome with shorter-than-normal QT intervals. Biophysically-detailed computational models of human atrial cells were modified to incorporate the functional impact of the *CACNA1C* encoded A39V and G490R mutations on the reduction of the maximal conductance (g_CaL_) of L-type calcium channels (LTCC). Varying levels of g_CaL_ reduction were considered. Effects of deficient LTCC on atrial excitation and propagation were investigated by using cellular and multi-dimensional tissue models that included a one-dimensional atrial strand, a two-dimensional idealized atrial sheet and three-dimensional human atria with realistic anatomical structure and detailed electrophysiology. Our results showed that reduced LTCC activity from the *CACNA1C* A39V and G490R mutations accelerated atrial repolarization, leading to shortened action potential duration and effective refractory period, as well as the loss of their rate-dependence. At the tissue level, decreased g_CaL_ shortened the wavelength of atrial excitation waves, slowed down atrial conduction velocity (CV) at low pacing rates but increased it at high pacing rates. It also showed bi-phasic arrhythmogenic effects in One-dimensional (1D), Two-dimensional (2D) and Three-dimensional (3D) tissue simulations. A large reduction in I_CaL_ increased tissue susceptibility to initiation and maintenance of atrial re-entrant excitation waves, while a moderate reduction showed anti-arrhythmic effects due to an increased meandering area of re-entrant excitation waves that led to early self-termination of the reentry. In conclusion, this study provides new mechanistic insights into understanding of biphasic effects of loss-of-function LTCC mutations on atrial pro-arrhythmias.

- Deficient I_CaL_ identified in patients with a phenotype combining Brugada syndrome with shorter-than-normal QT intervals has shown to be pro-arrhythmic, but the underlying mechanisms are unclear.
- This study used multi-scale computational models of the human atria to investigate potential pro-arrhythmic mechanisms of deficient I_CaL_.
- Reduced I_CaL_ abbreviated atrial APD and ERP, as well as the loss of their rate-dependence. It increased tissue vulnerability to initiation and maintenance of reentrant excitation waves. Such pro-arrhythmic effects are bi-phasic, depending on the degree of I_CaL_ deficiency.
- This study provides new mechanistic insights into understanding of biphasic effects of loss-of-function LTCC mutations on atrial pro-arrhythmic effects.

## 1. Introduction

The importance of calcium ions for cardiac electrical and mechanical activity has long been known [1,2]. L-type calcium channels (LTCCs) which mediate the long-lasting (L-type) calcium current (I_CaL_) provide the primary source of the inward Ca^2+^ flux that initiates excitation-contraction coupling, and maintains the plateau phase of cardiac myocyte action potentials (APs) [3]. LTCCs belong to the category of high voltage-activated channels (VGCCs) (also known as dihydropyridine (DHP) sensitive channels in cardiac biology), which are composed of multiple-subunits, including the main pore forming α-subunits of cardiac channel isoforms (Cav1.2 and Cav1.3), which mediate Ca^2+^ influx into cardiac myocytes [4].

Missense mutations to *CACNA1C*-encoded α-subunits (A39V, G490R, G1991R, R828H mutations) cause either loss-of-function or gain-of-function of LTCC, leading to dysfunctional I_CaL_ and increased arrhythmia risk [5–7]. It has been shown that gain-of-function of LTCC missense mutations are associated with Timothy syndrome (also called long QT syndrome 8; LQT8) with or without extracardiac phenotypes [8]. Patients with Timothy syndrome/LQT8 often show typical phenotypes of prolonged QT interval, T-wave alternans, and atrioventricular block, leading to high risk of sudden death [8–10]. On the other hand, loss-of-function of LTCC missense mutations have been identified in patients with a phenotype of short QT intervals, sometimes mixed with a Brugada-syndrome phenotype [5,11].

The short QT syndrome (SQTS) is associated with a high incidence of cardiac arrhythmias, including ventricular, atrial fibrillation and even sudden cardiac death [12,13]. So far, nine different genes have been implicated in the SQTS [14–17]. This includes three variants (SQT-1, SQT-2 and SQT-3) caused by gain-of-function mutations to genes encoding three potassium channel proteins: hERG, KCNQ1 or Kir2.1; these variants result in augmented I_Kr_, I_Ks_ and I_K1_ respectively [18–21]. Using multi-scale computational modelling approaches, potential mechanisms underlying the pro-arrhythmic effects of the increased potassium channel currents on ventricular and atrial fibrillation have been investigated [22–30].

A combined SQTS-Brugada-like phenotype has been associated with dysfunction mutations to genes encoding the α_1_- and β_2b_-subunits of the L-type calcium channel [31,32]. Antzelevitch et al. reported a new clinical entity of combined Brugada syndrome phenotype and shorter-than-normal QT intervals in patients with a family history of sudden cardiac death [5]. In that study, after screening 82 probands with Brugada syndrome, gene mutations to the α_1_ (A39V and G490R to *CACNA1C*; SQT-4) and β_2b_ (S481L to *CACNB2b*; SQT-5) subunits of L-type calcium channels were identified in the probands exhibiting short QT_c_ intervals [5]. Later, Templin et al. reported loss-of-function mutation to *CACNA2D1* gene (SQT-6), which also resulted in an abbreviated QT interval and ventricular fibrillation in patients (SQT-6) [11].

Atrial arrhythmias have been documented in patients with SQT-4/Brugada *CACNA1C* mutations. In the study by Antzelevitch et al. [5], the proband with *CACNA1C* Cav1.2 G490R mutation was a 41-year-old male who presented with AF and a QT_c_ interval of 346 ms, which showed poor rate adaptation. The patient showed no structural heart disease, but monomorphic VT could be elicited by programmed electrical stimulation. The proband with the *CACNA1C* Cav1.2 A39V mutation was a 44-year-old male with prominent ST elevation in V_1_ and saddleback ST elevation in V_2_ and a QT_c_ interval of 360 ms [5]. Electrophysiological investigation of recombinant Ca^2+^ channels using Chinese hamster cells (CHO) showed that both *CACNA1C* G490R and A39V mutations led to marked reductions in ${\text{I}}_{\text{CaL}}$ due to a trafficking defect for the *CACNA1C* A39V mutation and to reduced I_CaL_ without trafficking impairment for the *CACNA1C* G490R mutation.

Defective L-type calcium channels have been shown to be related to arrhythmogenic cardiac diseases, such as atrial fibrillation and heart failure [33–37]. In those cases, it was believed that a marked reduction in I_CaL_ may increase the risk of atrial fibrillation. Although it is known that the *CACNA1C* A39V and *CACNA1C* G490R mutations lead to reduced I_CaL_, the mechanisms underlying the pro-arrhythmic consequences for atrial electrophysiology are not well understood; it is unclear either how the mutations affect intrinsic atrial heterogeneity and influence dispersion of repolarisation that would form substrates for arrhythmogenesis.

Mathematical and computational modelling is valuable for the investigation of cardiac function in normal and pathophysiological conditions [38]. It also provides an alternative method for the interrogation of the functional consequences for cardiac electrophysiology of gene mutations, where appropriate genetically modified animal models are lacking. Therefore, this study implemented multi-scale computer modelling approaches, to investigate possible causative links between a reduction/loss of LTCC channel activity and arrhythmogenesis in AF. Effects of the *CACNA1C* A39V and *CACNA1C* G490R mutations and of graded reductions in I_CaL_ were investigated.

## 2. Methods

In this study, the Courtemanche–Ramirez–Nattel (CRN) human atrial cell action potential model was used [39]. The original CRN model has been updated for several ion channels, including the transient outward potassium current (${\text{I}}_{\text{to}}$) by Maleckar et al. [40] and the ultra-rapid rectifier potassium current (${\text{I}}_{\text{Kur}}$) by Colman et al. [41]. Additionally, models of human atria cells in different regions have also been developed to account for tissue electrophysiological heterogeneity [24]. Parameters for the ratio of maximal ion channel conductance between different cell types in relative to the right atria (RA) cell type have previously been discussed in a study by Whittaker et al. [28]; these formed the basal models of this study for simulating atrial heterogeneity. Details of the models and their parameters for representing different cell types of the atria incorporating regional differences in cellular electrophysiological properties have been detailed in our previous study [24].

### 2.1 Model of ${𝐈}_{𝐂𝐚𝐋}$for WT and mutation conditions

The model equations for the L-type calcium channel current in the CRN model, described in the Online Supporting Materials (Section 1 in S1 Text) in detail, were updated to simulate ${\text{I}}_{\text{CaL}}$in control and deficient conditions based on biophysical experimental data of Antzelevitch et al. for the *CACNA1C* A39V and *CACNA1C* G490R mutations [5,39]. The updated ${\text{I}}_{\text{CaL}}$ model equations were then incorporated into the updated CRN cellular and tissue models [28,39]. To validate the developed ${\text{I}}_{\text{CaL}}$ models, simulations of normalized voltage-current (I-V) relationships for wild-type (WT) and *CACNA1C* A39V and *CACNA1C* G490R mutation conditions were compared to experimental data obtained by using the same patch-clamp protocols as used experimentally by Antzelevitch et al. [5]. Results are shown in S1 Fig in the Online Supporting Materials (Section 2 in S1 Text) for comparing the simulation data to experimental data of Antzelevitch et al. [5] and corresponding parameters modifying CRN model to incorporate experimental data were listed in S1 Table in the Supporting Materials (Section 2 in S1 Text).

However, incorporation of the updated WT ${\text{I}}_{\text{CaL}}$ equations shown in S1 Fig (Online Supporting Materials) into the WT atrial cell models produced an abnormally shorter action potential duration at 90% repolarization (${\text{APD}}_{\text{90}}$) as compared to that of normal healthy atrial cells. This might be due to the lack of experimental data on the changes of activation and inactivation kinetics of ${\text{I}}_{\text{CaL}}$ in the study of Antzelevitch et al. [5], as well as the fact that those data were from recombinant channels expressed in CHO cells at room temperature rather than from native human atrial cells at body temperature. Considering the fact that for both the *CACNA1C* A39V and *CACNA1C* G490R mutations, Antzelevitch et al. [5] showed that the voltage at which the peaks of the I-V relationship for both WT and mutation conditions remained unchanged, indicating no shift in the voltage at which the I_CaL_ peak current occurred between WT and mutation conditions, it was suggested that the primary distinction in the I-V relationship between WT and mutation stems from the maximal conductance of LTCC. Consequently, in our simulations we utilised a decreased maximal conductance as a simplified representation of the reduced I_CaL_ associated with the mutations.

${\text{I}}_{\text{CaL}}$ in the basal CRN model of is given by the equation:


$$\begin{array}{c} I_{CaL}=g_{CaL}\cdot d\cdot h\cdot h_{Ca}\cdot \left(V_{m}-E_{Ca}\right), \end{array}$$ (1)

where $g_{CaL}$ is the maximal macroscopic channel conductance, *d* and *h* are voltage-dependent activation and inactivation gate variables respectively, and $h_{Ca}$ is the intracellular calcium dependent inactivation gate variable. In Equation (1), $V_{m}$ is the cell transmembrane potential and $E_{Ca}$ the reversal potential of the L-type calcium channel. Details of the formula of ${\text{I}}_{\text{CaL}}$ in the CRN model are presented in the Online Supporting Materials (S1 Text). In simulations, the time constant of $h_{Ca}$ was set to a constant (2.0 ms), as used by Colman et al [24].

As for both of the *CACNA1C* A39V and *CACNA1C* G490R mutations, a significant decrease in ${\text{I}}_{\text{CaL}}$ density was observed, the following equation of mixed WT and mutant I_CaL_ was used to simulate the reduced ${\text{I}}_{\text{CaL}}$ caused by the mutations, as well as for mimicking a general case of ${\text{I}}_{\text{CaL}}$ reduction,


$$\begin{array}{c} I_{CaL}^{\prime }=(\text{1}-f)\cdot I_{CaL}^{WT}+f\cdot I_{CaL}^{mut}, \end{array}$$ (2)

where $I_{CaL}^{WT}$ and $I_{CaL}^{mut}$ are the L-type calcium channel currents in WT and mutation conditions, and the proportion factor *f* was used to denote their relative contribution to the total $I_{CaL}^{\prime }$. In simulations, conditions of WT, heterozygous and homozygous mutation conditions were denoted by *f *= 0, 0.5 and 1 respectively; and other general conditions of intermediate I_CaL_ deficiency were simulated by 0 < *f* < 1.

For the use of *f* = 0.5 for mimicking the heterozygous mutation, i.e., a 50% wild-type and 50% mutant channel composition, we followed a common assumption that WT and mutant alleles contribute equally to the functional channel population. However, the actual distribution of functional channels with and without the mutation remains uncertain. For instance, variable expressivity could result in currents that more accurately reflect a different WT-to-mutant channel ratio than the presumed 50:50 distribution. Consequently, our simulations, which focus solely on conductance ratios, do not necessarily accurately represent physiological heterozygous conditions when using a fixed 50:50 ratio. Therefore, a graded scaling factor in our simulations was implemented for modeling different extents of I_CaL_ reduction, including those ratios (0, 0.5 and 1) assumed to represent the WT, heterozygous, and homozygous cases.

In simulations, ${\text{I}}_{\text{CaL}}^{\text{WT}}$ was computed using the basal CRN model and ${\text{I}}_{\text{CaL}}^{\text{mut}}$ was computed by reducing the maximal macroscopic channel conductance of ${\text{I}}_{\text{CaL}}$ in the CRN model proportionally based on the experimental data of Antzelevitch et al. [5].

For the mutant LTCC current, ${\text{I}}_{\text{CaL}}$ was simulated by:


$$\begin{array}{c} I_{CaL}^{mut}=g_{CaL}^{mut}\cdot d\cdot h\cdot h_{Ca}\cdot \left(V_{m}-E_{Ca}\right), \end{array}$$ (3)

where $g_{CaL}^{mut}$ represents for the maximal macroscopic channel conductance of the LTCC in mutations. The relative values of $g_{CaL}$ for the G490R, A39V (exon 8A), and A39V (exon 8) mutations were set to be 0.07, 0.13, and 0.20, respectively, in relation to the value in the original CRN model for the WT condition. These values were derived from experimental data quantifying the relative reduction in I_CaL_ compared to the WT condition [5], as extracted from Supplementary S1 Table and subsequently documented in S2 Table (Section 3 in S1 Text). Other parameters retained their previous definitions.

**Fig 1:**
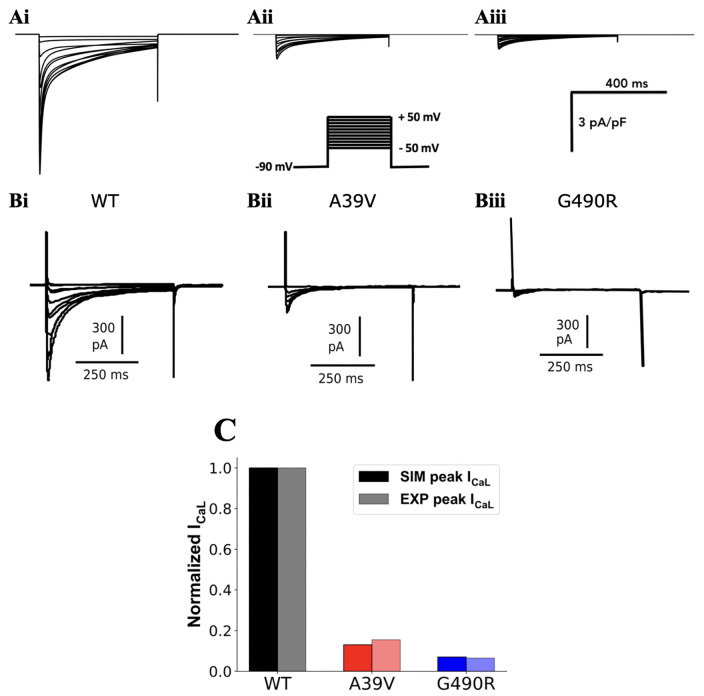
Simulated ${𝐈}_{\text{CaL}}$ traces during voltage-clamp for WT and *CACNA1C* A39V and *CACNA1C* G490R mutations by using the simple approach of g_CaL_ reduction for the mutation conditions. (A) Simulation data. (B) Experimental data reproduced from Antzelevitch et al. [5]. The patch-clamp protocol (inset) used in simulations is the same as used in Antzelevitch et al. [5]. It had a holding potential of -90 mV, and testing potentials ranging from -50 mV to 50 mV with an increment of 10 mV. In the panel, (i), (ii) and (iii) denote WT, *CACNA1C* A39V and *CACNA1C* G490R mutations respectively. (C) Normalized peak ${𝐈}_{\text{CaL}}$ amplitudes and their relative reduction as compared to the WT condition. Simulated (dark bars) and experimental (pale bars) I-V relationship peaks for WT (black), A39V (red), G490R (blue). The amplitude of I_CaL_ in mutation conditions was normalized to the WT condition for both experimental and simulation settings.

Fig 1 shows the validation of the mutant ${\text{I}}_{\text{CaL}}$ model by using the simplified approach of decreasing the maximal channel conductance of I_CaL_ for modelling *CACNA1C* A39V and *CACNA1C* G490R mutation conditions, in which the simulated ${\text{I}}_{\text{CaL}}$ traces by using the same voltage-clamp protocol as used experimentally were plotted. It was shown that the simulated ${\text{I}}_{\text{CaL}}$ in *CACNA1C* A39V (Fig 1, Aii) and *CACNA1C* G490R (Fig 1, Aiii) mutation conditions were much smaller than that in WT condition (Fig 1, Ai), which agreed with the experimental data of Antzelevitch et al. [5] (Fig 1, Bi, Bii and Biii). In Fig 1C, the relative reduction in the normalized peak amplitude of simulated ${\text{I}}_{\text{CaL}}$ in the I-V relationship in the mutation conditions, as compared to the WT condition, closely resembled that seen in the experimental data, validating the adequacy of the simple conductance-scaling approach for capturing the mutation-induced relative reduction of ${\text{I}}_{\text{CaL}}$ in A39V and G490R conditions.

### 2.2 Simulations of atrial myocytes

The updated ${\text{I}}_{\text{CaL}}$ equations for WT and CACNA1C A39V/G490R mutation conditions were incorporated into the single-cell model of Colman et al. [24], in which the change in the transmembrane potential of an individual cell is given by:


$$\begin{array}{c} \frac{{dV}_{m}}{dt}=\;-\;\frac{I_{ion,tot}+I_{st}}{C_{m}}, \end{array}$$ (4)

where V_m_ is the transmembrane potential, I_ion,tot_ the total ionic current including ${\text{I}}_{\text{CaL}}$, ${\text{I}}_{\text{st}}$ the stimulus current, and C_m_ the membrane capacitance.

To solve Equation (4) of the action potential (AP) model, the forward explicit Euler method was employed, using a time step 0.01 ms. The Rush-Larsen method was utilized to solve ordinary differential equations of gating variables of ion channels [42]. The functional impact of the reduced ${\text{I}}_{\text{CaL}}$ condition was quantitatively assessed via effects on the characteristics of APs, such as the amplitude of action potentials (APA), the maximum upstroke velocity (MUV) during depolarization phase, the resting membrane potential (RMP), the action potential duration at 90% repolarization level (${\text{APD}}_{\text{90}}$), the effective refractory period (ERP) and the Action Potential Duration (APD) and ERP restitution curves. Details of the methods for single-cell simulations are documented in Section 4.1 in S1 Text of the Online Supplementary Materials.

### 2.3 Multidimensional tissue level

The monodomain equation for cardiac tissue models was employed, which takes the form:


$$\begin{array}{c} \frac{\partial V_{m}}{\partial t}=\nabla \cdot D\nabla V_{m}-\frac{I_{ion,tot}+I_{st}}{C_{m}}, \end{array}$$ (5)

where **D** is the diffusion tensor and ∇ is the 3D spatial gradient operator, and other parameters retained their previous definition.

In numerical simulations, the explicit finite difference method (FDM) was employed to solve Equation (5) with non-flux boundary conditions at the edges, in order to obtain numerical solutions of AP across the tissue [43–45].

*1D simulations.* The 1D strand model consisted of 100 isotropic myocardial cells, arranged in a linear configuration with a spacing of 0.25 mm between each cell. The diffusion parameter D is a scalar coefficient in the 1D model, taking the same value as that in our previous study, 0.21 mm^2^ ms^−1^ [28].

Using the 1D strand model, tissue excitation properties, including the restitution curves of the CV, ERP and the wavelength (WL) of excitation waves, were quantitatively assessed for the wild-type, mutation conditions and general conditions of ${\text{I}}_{\text{CaL}}$ deficiency. To characterise the functional impact of reduced I_CaL_ on tissue excitability to provide mechanistic insights into its pro-arrhythmogenic effects, the excitation threshold measured as the minimal amplitude of an external stimulus (with a duration of 2 ms) to evoke a full action potential was computed. The rate-dependence of the atrial excitability in the 1D model was investigated in the WT, mutation conditions and ${\text{I}}_{\text{CaL}}$ deficient cases. Details of the methods for conducting the 1D simulations (computing CV, ERP, WL and the excitation threshold) are given in Section 4.2 in S1 Text, S2 Fig of the Online Supporting Materials.

*Tissue vulnerability.* APD dispersion at junctions of two distinctive atrial regions was also quantified using the 1D model as it is related to tissue vulnerability for generating uni-directional conduction block in response to a premature stimulus at the junctions [24]. The temporal vulnerable window (VW), defined as the period during which a premature stimulus (S2) applied to the proceeding excitation wave refractory tails in cardiac tissue causes uni-directional conduction block was measured. Such uni-directional conduction block may result in the formation of re-entrant excitations, underlying a major mechanism of atrial fibrillation [46–48]. The greater the VW width, the more prone the tissue to arrhythmogenesis. In this study, VW was measured at two junctions following previous studies [49–51], which included the junction at the crista terminalis (CT)/ pectinate muscles (PM) and the left atrium (LA)/ pulmonary veins (PV). Details of the one-dimensional simulations are presented in the Online Supporting Materials (Section 4.2 in S1 Text).

*2D simulations of re-entrant excitation waves.* Simulations using an idealised 2D sheet were performed to investigate the dynamic behaviours of re-entrant excitation waves. The 2D model had a size of 100 × 100 ${mm}^{\text{2}}$, which was discretised into 400 × 400 nodes by using a spatial resolution of 0.25 mm. To initiate reentry in the 2D tissue model, an S1-S2 stimulus protocol was used. The S1 stimulus was applied at the bottom of the 2D sheet tissue to generate a planar wave, and the S2 stimulus was applied on the quarter area at the lower left corner of the 2D sheet. The S1-evoked excitation wave was blocked and interacted with the excitation wave generated by the S2 stimulus, leading to the formation of re-entry. Details of the S1-S2 stimulus protocol used in 2D simulations to generate spiral waves are presented in the Online Supporting Materials (Section 4.3 in S1 Text, S3 Fig). In simulations, the tip trajectory of reentrant excitation waves was traced by using the method of phase singularities [52], and the functional impacts of the deficient ${\text{I}}_{\text{CaL}}$ conditions on the tip meandering pattern and the average dominant frequency (DF) of re-entrant excitation waves were investigated.

*3D realistic anatomical model.* The realistic anatomical structure of human heart was employed in our 3D simulations. The 3D model took the spatial heterogeneity in electrophysiological properties, anisotropic in atrial fibre spatial arrangements and electrical coupling into account, as developed in our previous studies [24,28]. Measurements of the spatial distribution of ${\text{APD}}_{\text{90}}$ (i.e., atrial repolarisation dispersion) across the atria and the average lifespan of re-entrant excitations (scroll waves) in WT and the deficient ${\text{I}}_{\text{CaL}}$ conditions were taken to investigate the tissue susceptibility and the dynamics of re-entry. To initiate reentry in the 3D model, phase distribution method was used [53]. Details of the methods used in 3D simulations including the phase distribution method for initiation of 3D reentry are documented in the Online Supporting Materials (S4 Fig and S3 Table, Section 4.4 in S1 Text).

*Pseudo-*Electrocardiogram *(ECG)*. The integrated method was utilized to calculate the integrated transmembrane potential of the atria, according to [54,55], referred to as a pseudo-ECG, at an electrode $\left({\text{x}}^{\prime },{\text{y}}^{\prime },{\text{z}}^{\prime }\right)$, by these following equations:


$$\begin{array}{c} \emptyset^{\prime }\left(x^{\prime },y^{\prime },z^{\prime }\right)=\int (-\nabla V)\cdot \left(\nabla \frac{\text{1}}{r}\right)d\Omega , \end{array}$$ (6)

$$\begin{array}{c} r=x(x,y,z)-\;x^{\prime }\left(x^{\prime },y^{\prime },z^{\prime }\right) \end{array}$$ (7)

where vector 𝐫 is the position difference vector from an electrical source x(x,y,z) at the atria to the electrode ${x^{\prime }(x}^{\prime },y^{\prime },z^{\prime })$. And Ω is the integration domain of the whole atria. Using the computed pseudo-ECG, the dominant frequencies of reentrant excitation waves could be calculated by the fast Fourier transformation method [56]. Details for computing the pseudo-ECG and locations for the placement of electrode to register the pseudo-ECG in the 2D and 3D models are given in S1 Text.

## 3. Results

### 3.1 Effects of reduction in ${𝐈}_{𝐂𝐚𝐋}$ on atrial APs and APD restitution properties

**Fig 2:**
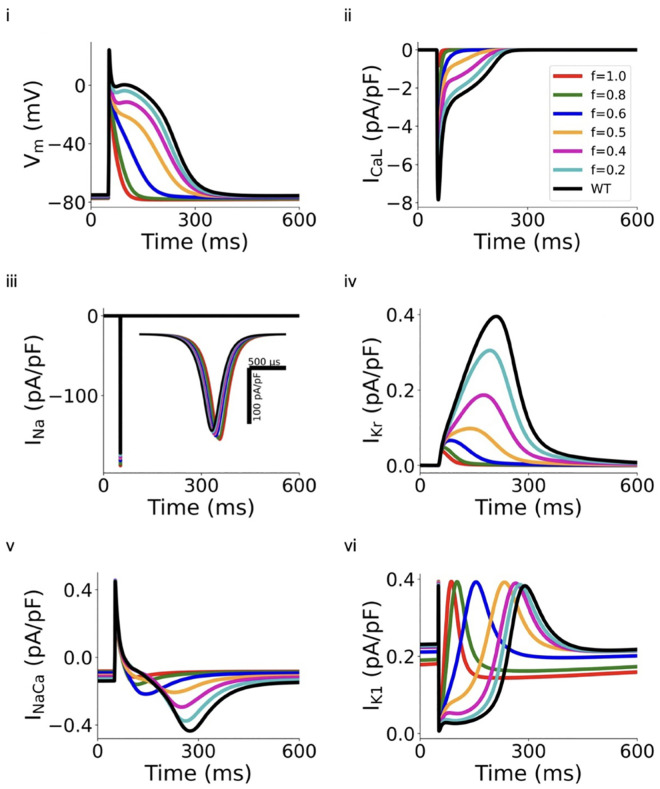
Computed APs and related ion channel currents in WT, G490R mutation (*f* = 1) and intermediate ${𝐈}_{CaL}$ deficiency (*f* = 0.2, 0.4, 0.5, 0.6 and 0.8) conditions. (i) APs. (ii) ${𝐈}_{\text{CaL}}$. (iii) ${𝐈}_{\text{Na}}$. (iv) ${𝐈}_{\text{Kr}}$. (v) ${𝐈}_{\text{NaCa}}$. (vi) ${𝐈}_{\text{K1}}$.

We first investigated the functional consequences of reduced ${\text{I}}_{\text{CaL}}$ arising from the *CACNA1C* G490R mutation condition, on atrial APs. Fig 2 shows results computed from the right atrial cell model as results from other atrial cell types were similar. In the figure, results from the *CACNA1C* G490R mutation only were shown as results from the *CACNA1C* A39V and A39V (exon 8) mutations are quantitatively similar (S4 Table and S5 and S6 Figs, Section 5.1 in S1 Text in the Online Supporting Materials).

Fig 2i shows the computed APs from WT (*f* = 0), heterozygous (*f* = 0.5) and *CACNA1C* G490R (*f* = 1) mutation, and other intermediate deficient ${\text{I}}_{\text{CaL}}$ (*f* = 0.2, 0.4, 0.6 and 0.8) conditions. As compared to WT condition, reduced ${\text{I}}_{\text{CaL}}$ accelerated atrial repolarization, leading to abbreviated APD (from 247.4 ms in WT to 192.4 ms and 41.7 ms for heterozygous and homozygous *CACNA1C* G490R mutation conditions respectively). With a gradual increase of *f* (i.e., an increased level of ${\text{I}}_{\text{CaL}}$ deficiency), the computed ${\text{APD}}_{\text{90}}$ decreased monotonically as shown in Table 1. In addition, the deficient ${\text{I}}_{\text{CaL}}$ also altered the AP morphology, which changed from a spike-dome shape to a triangular shape in WT and *CACNA1C* G490R mutation conditions. The reduced ${\text{I}}_{\text{CaL}}$ also slightly hyperpolarised resting membrane potential, but increased the amplitude and the maximal upstroke velocity as shown in Table 1.

**Table 1:** Effects of reduced $I_{CaL}$ on AP characteristics. As compared to WT, G490R mutation condition (*f* = 1) and intermediate ${\text{I}}_{\text{CaL}}$ deficiency (*f* = 0.2, 0.4, 0.5, 0.6 and 0.8) increased the amplitude of action potential and the maximal upstroke velocity, but caused a more negative resting membrane potential and reduced action potential durations.

WT/MT	𝐀𝐏𝐀(𝐦𝐕)	MUV(V/s)	𝐑𝐌𝐏(𝐦𝐕)	${𝐀𝐏𝐃}_{90}\left(𝐦𝐬\right)$
WT	99.2	192.0	-75.6	247.4
*f* = 0.2	99.6	194.8	-75.8	234.6
*f* = 0.4	100.0	197.4	-76.1	223.1
*f* = 0.5	100.1	198.4	-76.1	192.4
*f* = 0.6	100.6	201.3	-76.8	113.4
*f* = 0.8	101.0	205.2	-77.8	58.3
*f* = 1.0	101.1	206.9	-78.3	41.7

The reduced I_CaL_ was associated with abbreviation of atrial APs (Fig 2ii). Secondary effects of I_CaL_ reduction were observed on other currents, including a slight increase in I_Na_ (Fig 2iii), reduced outward ${\text{I}}_{\text{Kr}}$ (Fig 2iv), decreased amplitude of ${\text{I}}_{\text{NaCa}}$ (Fig 2v), attenuated outward ${\text{I}}_{\text{K1}}$ (Fig 2vi) during repolarisation ${\text{g}}_{\text{CaL}}$. The slight increase in APA might be caused by an increased ${\text{I}}_{\text{Na}}\;$(Fig 2iii) due to a more negative RMP, which increased the activation of ${\text{I}}_{\text{Na}}$channel. The more negative RMP was also related to the decreased inward component of ${\text{I}}_{\text{NaCa}}$ (Fig 2v) and other secondarily-affected ion channels as there was negligible change in ${\text{I}}_{\text{K1}}$ (Fig 2vi) at the resting potential.

**Fig 3:**
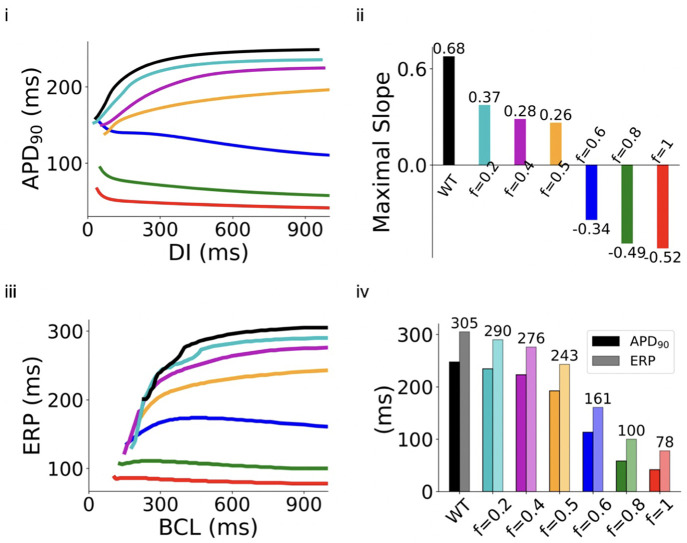
Simulations at the single cell level: (i) APD restitution (APDr) curve. (ii) The maximal slope of the APDr curves. (iii) ERP restitution (ERPr) curves. (iv) APD and ERP versus various f in G490R mutations.

Reductions in ${\text{I}}_{\text{CaL}}$ caused a loss of rate dependence of atrial excitation as shown in Fig 3i and 3ii, in which computed ${\text{APD}}_{\text{90}}$ at different diastolic intervals (DIs) was plotted to reconstruct the APD restitution (APDr) curves. As compared to the WT condition, deficiency of ${\text{I}}_{\text{CaL}}$ flattened the APDr curves (Fig 3i), which was reflected by decreased maximal slopes of those curves (Fig 3ii). At all pacing rates considered, ${\text{I}}_{\text{CaL}}$ deficiency shortened ${APD}_{\text{9}\text{0}}$. However, it showed subtle differences in modulating the rate-dependence of APDr with different extents of I_CaL_ reduction. Though APDr curves with I_CaL_ reductions of *f *≤ 0.5, followed a similar trend (i.e., APD decreased with the decrease of DI) to that of WT, those with larger degrees of ${\text{I}}_{\text{CaL}}$ deficiency (f≥ 0.6 to *f *= 1) followed an opposite trend. In the latter cases, the computed APD increased rapidly with the decrease of DI at fast rates, resulting in negative slopes (Fig 3ii) of the APDr curves. In such a case, the deficient ${\text{I}}_{\text{CaL}}$ tissue showed supernormal excitability, facilitating atrial conduction at high rates, which is a typical feature of atrial fibrillation [57]. The A39V mutation and corresponding intermediate deficient cases exhibited similar results at the single cell level, as given in S5 and S6 Figs, Section 5.1 in S1 Text of the Online Supporting Materials.

In addition, the electrophysiological characteristics of APs in WT conditions and for all mutations (homozygous) were simulated in S7 Fig and S5 Table, Section 5.1 in S1 Text of the Online Supporting Materials. In order to test whether our simulation results are model-independent, the Grandi et al. model of human atrial cells [22] was also used to quantify the functional impacts of mutations due to reduced ${\text{I}}_{\text{CaL}}$ on the characteristics of APs. Results with the Grandi *et al*. model at the single cell level are provided in S8 Fig in the Section 5.1 of the Supporting Materials and are consistent with the results of the CRN models.

### 3.2 Effects on atrial conduction at the tissue level

**Fig 4:**
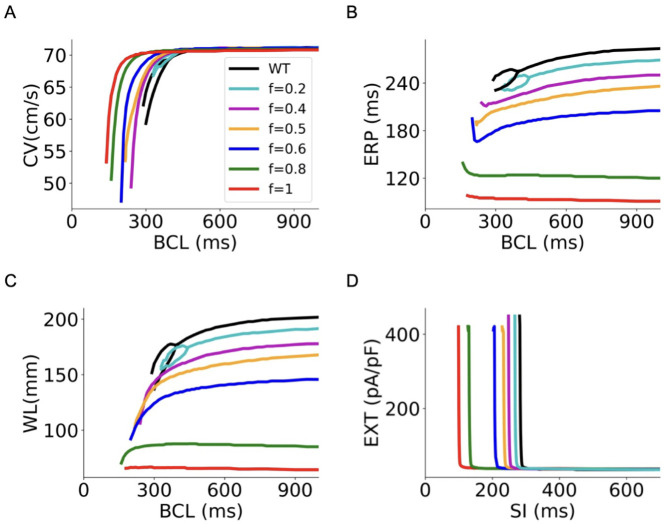
Restitution curves of CV, ERP, WL and excitation threshold (EXT) in WT, heterozygous (*f* = 0.5), homozygous (*f* = 1) *CACNA1C* G490R mutation and intermediate ${𝐈}_{CaL}$ deficiency (*f* = 0.2, 0.4, 0.6 and 0.8) conditions. (A) Rate-dependency of CV. Bifurcation of the CVr curve at WT and low degrees of I_CaL_ deficiency (*f* ≤ 0.2) was observed, indicating conduction alternans of electrical excitation waves. The bifurcation disappeared at a high degree of I_CaL_ deficiency (*f* > 0.2). (B) Rate-dependency of ERP. Bifurcation in the ERPr curves with low degrees of I_CaL_ deficiency was observed and disappeared at high degrees of I_CaL_ deficiency. (C) Rate-dependency of WL. Bifurcation in the WLr curves with low degrees of I_CaL_ deficiency was observed and disappeared at high degrees of I_CaL_ deficiency. (D) Tissue excitation threshold against S1-S2 intervals (SI).

***Conduction velocity and tissue excitability in 1D***. Using the 1D atrial strand model, we then investigated the consequences of reduced ${\text{I}}_{\text{CaL}}$ on the conduction velocity, effective refractory period, wavelength of excitation waves and tissue excitability (which was reciprocally measured by the excitation threshold (EXT) of tissue to evoke propagating excitation waves). Results are shown in Fig 4.

Fig 4A displays the computed CV restitution (CVr) curves of atrial tissue in WT and deficient ${\text{I}}_{\text{CaL}}$ conditions. As illustrated in the figure, reductions in G490R ${\text{I}}_{\text{CaL}}$ of up to *f* = 1 (the homozygous mutation condition) did not cause noticeable changes in the CV at slow pacing rates. At the basic cycle length (BCL) of 1000 ms, the measured CV was 71.1 cm/s in WT and 70.8 cm/s for the G490R mutation condition. In all cases, the measured CV decreased monotonically with the decrease in BCL. However, at high pacing rates, the measured CV dropped to zero at a cut-off BCL, below which atrial excitation failed to conduct. In the WT condition, the measured cut-off BCL was 280 ms, which was reduced by ${\text{I}}_{\text{CaL}}$ deficiency, as manifested by a leftward shift of the CVr curves. Thus, ${\text{I}}_{\text{CaL}}$ reduction facilitated atrial excitation waves at fast excitation rates that was not sustained in WT condition. Note that in the WT condition and when *f* ≤ 0.2, a bifurcation of the CVr curves was observed (black line(s)), indicating conduction alternans of excitation waves. This bifurcation disappeared at higher degrees of I_CaL_ deficiency (*f* > 0.2).

Fig 4B shows the computed ERP restitution curves in WT and deficient ${\text{I}}_{\text{CaL}}$ tissue models. It was shown that decreasing ${\text{I}}_{\text{CaL}}$ reduced ERP across all considered BCL range (e.g., in a range of 200 – 1000 ms). In WT (*f *= 0) and slight ${\text{I}}_{\text{CaL}}$ deficiency (*f* = 0.2) conditions, the measured ERP first decreased with a decrease in BCL, but a bifurcation occurred in the ERPr curve with a further decrease in BCL, indicating the genesis of atrial electrical alternans at these BCLs. The onset BCL for bifurcation to occur was greater in the slightly ${\text{I}}_{\text{CaL}}$ deficient tissue (*f* = 0.2) than that in WT tissue. Bifurcation in the ERPr curve disappeared when ${\text{I}}_{\text{CaL}}$ was further reduced (*f* > 0.2). In the cases of 0.4≤f<0.8 that included the heterozygous (*f* = 0.5) mutation condition, though the measured ERP followed a similar monotonic decrease with BCL to that seen in the WT case, it showed a sharp increase at very small BCLs before atrial conduction failed. With a substantial reduction of ${\text{I}}_{\text{CaL}}$ (*f* = 0.8) and the homozygous (*f* = 1) G490R mutation conditions, the measured ERP increased with the decrease in BCLs, indicating supernormal excitability at fast excitation rates in those settings. Such changes in the rate-dependence of ERP were also reflected by the WL of excitation waves in atrial tissue, as shown in Fig 4C. The wavelength at fast rates was larger than it was at slow rates with a large reduction in ${\text{I}}_{\text{CaL}}$. In the WT condition, the measured WL was 202.8 mm, which was reduced to 167.8 mm and 64.4 mm for the heterozygous and homozygous G490R mutation conditions respectively at a pacing BCL of 1000 ms.

**Fig 5:**
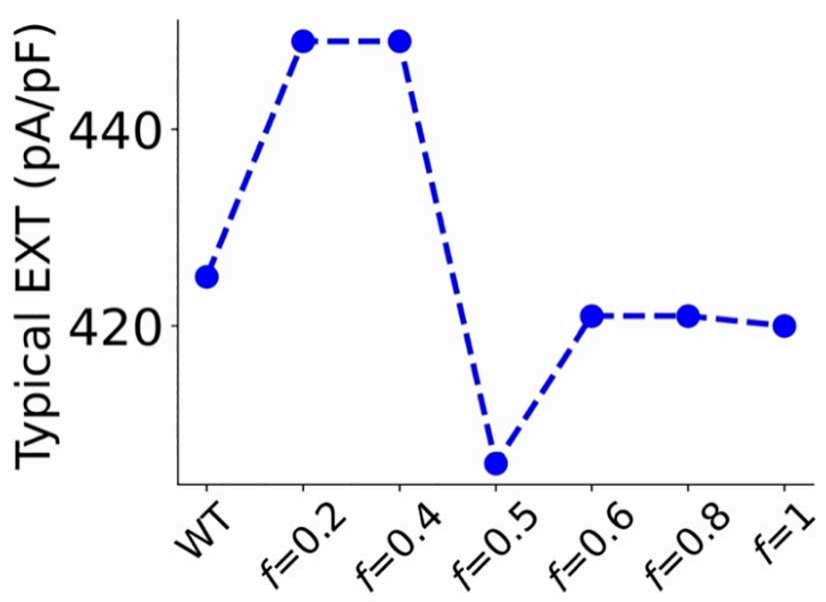
Computed excitation threshold at the critical SI (corresponding typical EXT) when significant alterations of tissue excitability were observed. The typical EXT at critical SIs: 282, 267, 248, 233, 206, 130 and 99 ms for WT and deficient ${𝐈}_{\text{CaL}}$ with f equal to 0.2, 0.4, 0.5, 0.6, 0.8 and 1 (*CACNA1C* G490R mutation) respectively.

Tissue excitability may affect atrial arrhythmia genesis and maintenance. The rate-dependent excitation threshold (EXT) measured as the minimal stimulus strength to generate propagating excitation waves in the 1D tissue model is shown in Fig 4D for WT and attenuated ${\text{I}}_{\text{CaL}}$ conditions. At slow pacing rates (e.g., SI = 700 ms), the computed EXT was 35 pA/pF for WT and 37 pA/pF for homozygous G490R mutation. In the intermediate ${\text{I}}_{\text{CaL}}$ deficiency and heterozygous G490R mutation cases, the measured EXTs at slow pacing rates did not show marked differences to that of WT tissue. However, at fast pacing rates (SI < 280 ms), the measured EXT showed marked differences between WT and reduced ${\text{I}}_{\text{CaL}}$ conditions. At a SI = 280 ms below which atrial excitability was lost in WT, the measured EXT in WT was over 500 pA/pF, but the measured EXT in deficient ${\text{I}}_{\text{CaL}}$ was around 37 pA/pF, largely smaller than it in WT. For the ${\text{I}}_{\text{CaL}}$ deficient tissue, the critical SI (i.e., at which the measured EXT increased sharply from below 250 pA/pF to over 400 pA/pF) was reduced as manifested by a leftward shift of the rate-dependent EXT curve, indicating atrial tissue sustained excitability at faster excitation rates in the deficient ${\text{I}}_{\text{CaL}}$ tissue than that in WT. At the critical SIs, atrial excitabilities underwent a significant alteration (i.e., a switch of EXT between >400 pA/pF and EXT < 250 pA/pF). The measured EXTs (at the critical SI) were plotted in Fig 5, with values listed in Table 2. It was shown that deficient I_CaL_ had a complex effect on atrial tissue excitability. It first increased EXT for small I_CaL_ deficiency (*f* < 0.4), then decreased it for intermediate I_CaL_ deficiency (*f* = 0.5) and increased it again from a greater I_CaL_ deficiency (f > 0.5).

**Table 2:** Measured corresponding excitation thresholds at critical SIs for WT and G490R-related deficient ${𝐈}_{CaL}$ atrial tissue.

@fast rates	𝐂𝐫𝐢𝐭𝐢𝐜𝐚𝐥 𝐒𝐈 (𝐦𝐬)	𝐂𝐨𝐫𝐫𝐞𝐬𝐩𝐨𝐧𝐝𝐢𝐧𝐠 𝐄𝐗𝐓 (𝐩𝐀/𝐩𝐅)
WT	282	425
*f* = 0.2	267	449
*f* = 0.4	248	449
*f* = 0.5	233	406
*f* = 0.6	206	421
*f* = 0.8	130	421
*f* = 1	99	420

**Fig 6:**
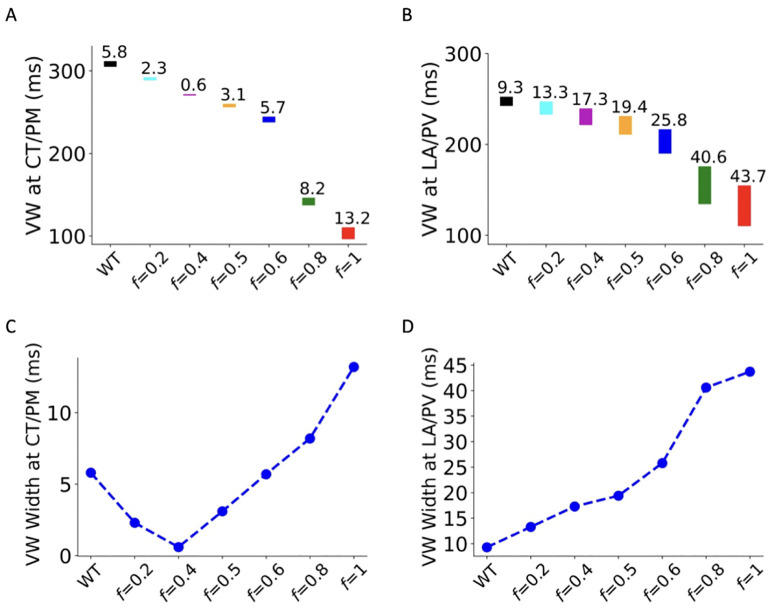
Computed temporal vulnerability of atrial tissue for WT, G490R and corresponding ${𝐈}_{CaL}$ deficient conditions at the CT/PM (A) and LA/PV (B) junctions. Biphasic variations in the measured width of VW at the CT/PM (C). At the LA/PV (D) junction, the VW width was increased with the increase of deficient coefficient *f*.

We further computed the temporal vulnerability window (VW) during which a premature stimulus induced uni-directional conduction block for WT and deficient ${\text{I}}_{\text{CaL}}$ tissue at CT/PM and LA/PV junctions. Results are shown in Fig 6. At the CT/PM junction (Fig 6A), VW width changed from 5.8 in WT ms to 3.1 ms and 13.2 ms in the heterozygous (*f *= 0.5) and homozygous (f = 1) G490R mutation condition respectively, and at the LA/PV junction (Fig 6B) it changed from 9.3 ms in WT to 19.4 ms and 43.7 ms for the heterozygous and homozygous *CACNA1C* A39V mutation respectively. In simulations of CT/PM junctions with intermediate ${\text{I}}_{\text{CaL}}$ deficiency, the measured VW width showed a biphasic trend: over the range of *f* values from 0 to 1 there was an initial decrease and then an increase, as depicted in Fig 6C. On the contrary, the LA/PV junction witnessed an increase in the width of VW in all deficient ${\text{I}}_{\text{CaL}}$ conditions. Measurements of VW at the CT/PM junction suggested an increased tissue susceptibility at corresponding regional tissue to uni-directional conduction in the G490R mutation and greatly deficient ${\text{I}}_{\text{CaL}}$ conditions, but reduced tissue susceptibility in conditions of smaller I_CaL_ deficiency. By contrast, the great enlargements of VW width at the LA/PV regional tissue represent the increased tissue susceptibility for all ${\text{I}}_{\text{CaL}}$ reductions tested. Similar results of VW width were observed for the A39V mutation and its-linked intermediate I_CaL_ deficient conditions, as shown in the Online Supplementary Materials (S9 Fig, Section 5.2 in S1 Text).

**Fig 7:**
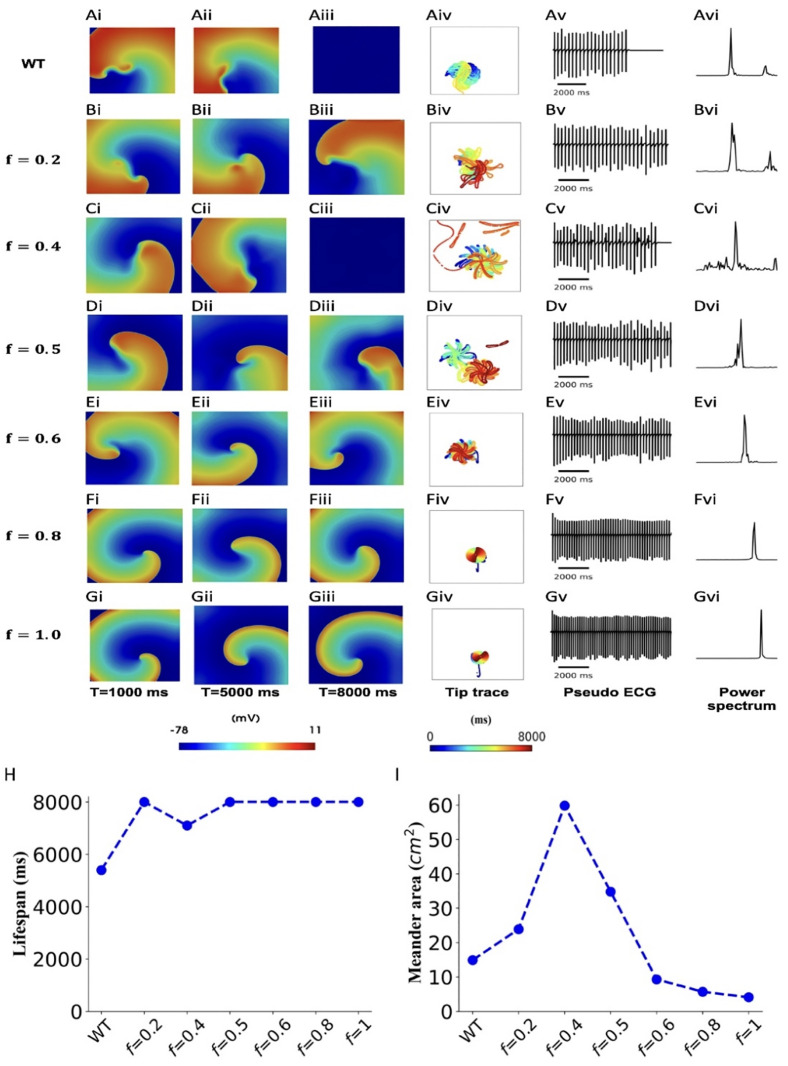
Dynamic behaviours of reentrant spiral waves in two-dimensional atrial tissue in WT (A), heterozygous (D; *f* = 0.5), homozygous *CACNA1C* G490R mutation (G; *f* = 1) and intermediate ${𝐈}_{\text{CaL}}$ deficiency (B, C, E &F) conditions. In each case, snapshots of reentry (e.g., Ai to Aiii), and tip trajectory pattern (e.g., Aiv), time series of integrated transmembrane potential across the entire 2D tissue (e.g., Av) and its power spectrum (e.g., Avi) are shown. The lifespan (H) of reentries and the meandering area of tip traces for WT, G490R and intermediate ${𝐈}_{\text{CaL}}$ deficient conditions (I).

***Effects of*** $I_{CaL}$ ***deficiency on dynamic behaviours of reentry***. Using the 2D model, we evaluated the functional impacts of the deficient ${\text{I}}_{\text{CaL}}$ on the dynamic behaviours of reentrant excitation waves (for more information see S1–S7 Videos in the Online Supplementary Materials). Results are shown in Fig 7, which shows the snapshots of reentry, tip trajectory pattern, time series of integrated transmembrane potential across entire 2D sheet and its power spectrum. Over the range of *f* values between (0.6 and 1), the spiral wave showed reduced wavelength with reduced meandering region as compared to that of WT, heterozygous (*f *= 0.5) or slightly deficient ${\text{I}}_{\text{CaL}}$ cases (*f* equal to 0.2 and 0.4). In the WT condition, spiral waves self-terminated with a lifespan 5500 ms. However, in the considerably ${\text{I}}_{\text{CaL}}$ deficient tissue (with *f* = 0.5, 0.6, 0.8 and 1), including heterozygous and homozygous G490R mutation conditions, re-entry sustained over the simulation period (8.0 s). In the case with *f* = 0.4, re-entry self-terminated with a lifespan 7100 ms. The tip trajectories of spiral waves in WT, G490R mutation and ${\text{I}}_{\text{CaL}}$ deficient tissues showed different patterns, with that in the WT and deficient $\begin{array}{c} {\text{I}}_{\text{CaL}} \end{array}$ with *f *< 0.5 cases showing a more complex petal shape with greater meandering areas than that in the cases of G490R mutation and *f* > 0.5 conditions, indicating that significant ${\text{I}}_{\text{CaL}}$ deficiency (*f* > 0.5) helped to sustain re-entries. The time series of integrated $V_{m}$ of cells across the 2D tissue showed more irregularity at slightly deficiency conditions (especially *f* = 0.4) than that in greatly ${\text{I}}_{\text{CaL}}$ deficient tissues, also suggesting a more stable reentry was generated in the ${\text{I}}_{\text{CaL}}$ highly deficient tissue. The computed dominant frequencies of the $V_{m}$ time series via fast Fourier Transformation (FFT) showed an increased atrial excitation frequency in ${\text{I}}_{\text{CaL}}$ deficient cases ${\text{I}}_{\text{CaL}}$, which was 3.8 Hz in WT, 4.9 Hz and 7.1 Hz for heterozygous (*f* = 0.5) and homozygous (*f* = 1) *CACNA1C* G490R mutant tissue. Details are given in Table 3. The measured area of the spiral wave core meandering also showed a biphasic change with the change of *f*. With the increase of *f* from 0 to 1, the meandering area increased firstly until *f* = 0.4, and then decreased, as illustrated in Fig 7I. Similar biphasic effects of meandering areas of reentrant waves’ tip were presented in A39V and relative ${\text{I}}_{\text{CaL}}$ deficient cases, as given in S6 Table and S10 Fig, section 5.3 in S1 Text in the Online Supporting Materials. All mutant tissue conditions exhibited pro-arrhythmic effects with decreased meandering areas and increased dominant frequency of time series (section 6.4 in the Online Supporting Materials).

**Table 3:** Measured characteristics of re-entrant spiral waves in 2D simulations for WT and the ${𝐈}_{CaL}$ deficient tissue. DF and the meandering area of spiral wave cores were shown.

	WT	*f* = 0.2	*f* = 0.4	*f* = 0.5	*f* = 0.6	*f* = 0.8	*f* = 1
DF (HZ)	3.8	3.9	4.2	4.9	5.2	6.1	7.1
Tip Meander Area (${\text{cm}}^{\text{2}}$)	14.9	23.9	59.6	34.8	9.3	5.7	4.1

**Fig 8:**
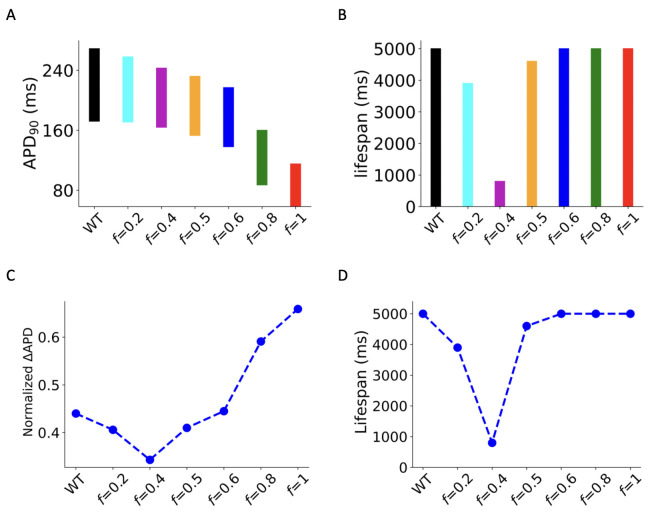
(A) Bar charts of $\begin{array}{c} {𝐀𝐏𝐃}_{90} \end{array}$ range across the whole 3D human atrial tissue in all types. (B) Lifespan of re-entrant scroll waves in WT, G490R mutation and deficient ${𝐈}_{\text{CaL}}$ conditions. (C) Normalized APD dispersion: $\begin{array}{c} {\Delta APD}_{90} \end{array}$ (normalized by the midrange of spatial $\begin{array}{c} {APD}_{90} \end{array}$ across the 3D atria) versus scaling factor *f*. (D) The lifespan of 3D re-entrant scroll waves with dashed lines.

***Pro-arrhythmic effects of markedly deficient*** $I_{CaL}$ ***in 3D simulations***. To test whether the pro-arrhythmic effects of markedly reduced ${\text{I}}_{\text{CaL}}$ seen in single cell, 1D and 2D tissue models are reflective of those in 3D model of the human atria with detailed anatomical structures, electrical heterogeneity and anisotropy, further simulations were conducted in the 3D model of human atria (for more information see S8–S14 Videos in the Online Supplementary Materials). For each simulation case of I_CaL_ deficiency, the initial conditions for the 3D model and the initiation of 3D reentry remained the same, allowing relative comparisons of results. Results are shown in Fig 8 for the measured ${\text{APD}}_{\text{90}}$ across the atria (Fig 8A) and the lifespan of scroll waves (Fig 8B). To assess the APD dispersion effects of varying reduced ${\text{I}}_{\text{CaL}}$ conditions, global APD dispersion was normalized by the mid-range of ${\text{APD}}_{\text{90}}$ across the atria in each case, as shown in Fig 8C, which also showed biphasic effects with the deficiency scaling factor *f*. A biphasic change of the normalized APD dispersion is consistent with the measured width of VW in 1D simulations at the CT/PM junctions. Similarly, a biphasic change in the measured lifespan of scroll waves in the 3D model against the scaling factor *f* was also observed. With *f* increasing from 0 to 1, the measured lifespan first decreased in the range of 0.2≤f≤0.4 and then increased for cases of f≥0.5 as shown in Fig 8D.

**Fig 9:**
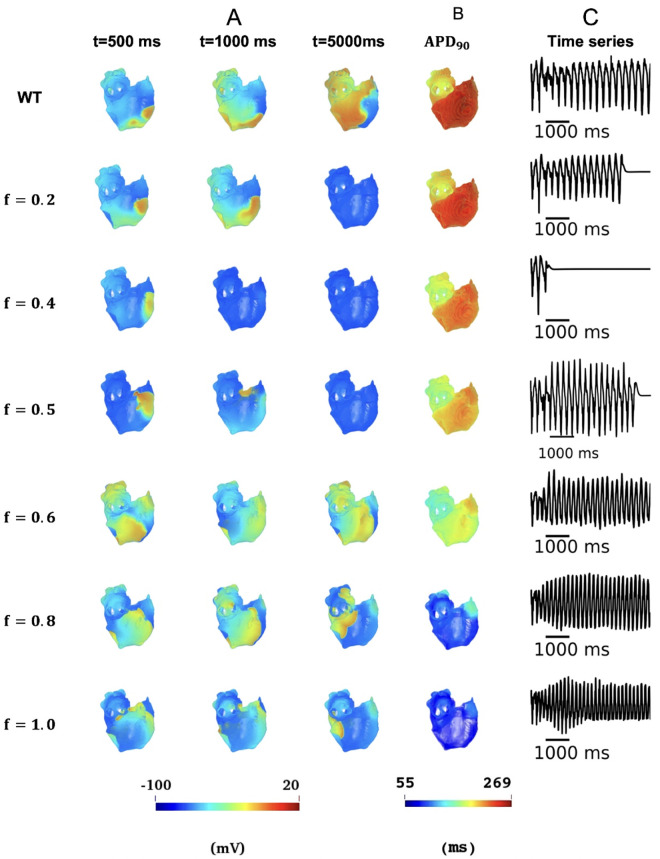
Characterisation of scroll wave dynamics and action potential properties in 3D human atrial simulations. (A) Temporal evolution of re-entrant scroll waves at representative time points (indicated above each panel) obtained from 3D computational simulations. The action potential distribution is represented using a colour gradient map ranging from -100 mV (blue) to +20 mV (dark red), with the corresponding colour scale provided below the panel. (B) Spatial distribution of APD heterogeneity across the 3D human atrial tissue. APD dispersion is visualised using a color-coded map ranging from 55 ms (blue) to 269 ms (dark red), with the colour scale displayed below the panel. (C) Temporal integration of the total membrane potential, calculated as the spatial integration of action potentials across the entire 3D human atrial tissue.

Fig 9 shows snapshots of scroll waves (panel A), APD spatial distribution in 3D human atrial model (panel B) and time series of integrated transmembrane potential (panel C) across 3D atrial tissue. The measured DF of the time series of scroll waves increased with the increase of *f* (see Table 4). A biphasic change of normalized APD dispersion and lifespan of three-dimensional reentries was also observed in A39V mutations and A39V-related intermediate ${\text{I}}_{\text{CaL}}$ deficiency, presented in S11 and S12 Figs and S7 Table, section 5.4 in S1 Text of the Online Supplementary Materials.

**Table 4:** Measured lifespan and computed dominant frequency of scroll waves in WT, *CACNA1C* G490R mutation and deficient ${\text{I}}_{\text{CaL}}$ conditions in 3D model.

WT/MT metrics	WT	*f* = 0.2	*f* = 0.4	*f* = 0.5	*f* = 0.6	*f* = 0.8	*f *= 1
Lifespan (ms)	5000	3900	800	4600	5000	5000	5000
DF (HZ)	3.7	3.9	3.9	4.3	4.9	5.7	6.2

For all homozygous mutations with large ${\text{I}}_{\text{CaL}}$ reduction, pro-arrhythmic effects were observed (S13–S17 Figs, Section 6 in S1 Text of the Online Supplementary Materials).

## 4. Discussion

In this study, functional consequences of loss-of-function of LTCC associated with G490R, and A39V Cav1.2 mutations and progressively scaled LTCC deficiency were investigated by using multi-scale computational models of the human atria, ranging from single cell level to 1D, 2D and 3D tissue levels. Our major findings are: (1) reductions in I_CaL_ arising from the G490R and A39V mutations accelerated atrial repolarization, resulting in APD shortening and ERP abbreviation. The abbreviation in APD and ERP were correlated with the degree of I_CaL_ reduction, characterized by a scaling factor *f* (*f *= 0 for WT, *f *= 0.5 and 1 for heterozygous and homozygous mutation conditions, respectively): the greater degree of I_CaL_ deficiency, the greater ${\text{APD}}_{\text{90}}$ and ERP abbreviation. The APD/ERP abbreviation was largely attributable to reduced I_CaL_ (the observed secondary effects on other ion channels would be expected to partially mitigate rather than augment APD/ERP abbreviation, except the reduction in inward I_NaCa_); ${\text{I}}_{\text{CaL}}$ (2) significant reduction in ${\text{I}}_{\text{CaL}}$ reduced tissue excitability at low pacing rates, but increased tissue excitability at fast pacing rates, resulting in supernormal excitability of atrial tissue, which was manifested by a negative maximal slope of the APD restitution curves. It also caused a loss of rate-adaptation of tissue APD and CV restitution properties; (3) markedly reduced ${\text{I}}_{\text{CaL}}$ shortened the wavelength of atrial excitation waves, leading to sustained reentry in ${\text{I}}_{\text{CaL}}$ deficient tissue. It also augmented APD dispersion at the CT/PM and LA/PV junctions, favouring the genesis of uni-directional conduction block predisposing to AF onsetting; (4) deficient ${\text{I}}_{\text{CaL}}$ had biphasic effects on atrial arrhythmogenesis. With an intermediate reduction in current (i.e., a small increase of the scaling factor f when it is ≤ 0.4), anti-arrhythmic effects were observed; while more extensively ${\text{I}}_{\text{CaL}}$ deficient tissue was pro-arrhythmic. Collectively, these results provide novel mechanistic insights into the understanding of arrhythmogenic effects of ${\text{I}}_{\text{CaL}}$ reduction.

***Secondary effects of LTCC mutation on other ion channels*.** Our simulations demonstrated that impairment of L-type calcium channel (LTCC) activity has a significant impact on sodium-calcium exchanger current ($I_{NaCa}$), primarily by reducing its inward current component. $I_{CaL}$ serves as the primary source of Ca² ⁺ influx during the action potential (AP), triggering calcium-induced calcium release (CICR) from the sarcoplasmic reticulum (SR). $I_{CaL}$ deficiency reduced the Ca² ⁺ influx into the myocytes, leading to a reduced intracellular Ca²⁺ ([Ca²⁺]_i_), which in turn reduced the extrusion of Ca^2+^ by sodium-calcium exchanger and coupled Na^+^ entry. This reduced the inward component of $I_{NaCa}$ during both the resting and plateau AP phases, which hyperpolarised the resting membrane potential. The hyperpolarised RMP increased sodium channel availability, thereby augmenting $I_{Na}$ during depolarisation. Furthermore, $I_{CaL}$ deficiency shortened AP duration by attenuating the plateau phase, resulting in a less activated I_Kr_.

***Combined effect of altered ion channel currents on APs.*** The altered AP profile was attributable to an integral action of all altered ion channel currents. As I_Na_ mediates the rapid depolarisation phase of the action potential (AP) (phase 0), the secondarily augmented I_Na_ caused an increased overshoot of AP and its amplitude. The secondarily reduced I_Kr_ might be anticipated to prolong APD, however, due to the substantial difference in the density of $I_{CaL}$ (~8 pA/pF in wild-type) and $I_{Kr}$ (~0.4 pA/pF in wild-type), the overall effect on AP duration was dominated by the reduced I_CaL_, leading to APD abbreviation.

***Ionic mechanism of APD shortening and ERP reduction.*** Our single-cell level simulation results showed that the observed atrial APD/ERP abbreviation was primarily attributable to the loss-of-function of LTCC associated with a decreased ${\text{I}}_{\text{CaL}}$. In the case of a large decrease in ${\text{I}}_{\text{CaL}}$, the plateau phase of the AP was nearly abolished, resulting in an accelerated repolarisation phase of AP, leading the AP profile to change from spike-dome shape in WT to triangle-shape in remarkdly deficient ${\text{I}}_{\text{CaL}}$ tissue.

***Mechanism for increased tissue susceptibility to reentry***. Tissue susceptibility to initiation of reentry can be quantified by the measured width of the temporal vulnerable window, during which a premature stimulus may evoke an excitation that propagates in the direction where tissue has recovered from its previous excitation, but fails to propagate in the other direction where tissue is still in their refractoriness. In this study, we used 1D tissue models to quantify the width of VW in WT, mutations and intermediate ${\text{I}}_{\text{CaL}}$ reduction tissue. While our results suggest that at intermediate levels of I_CaL_ reduction, the width of the VW decreased at the CT/PM junction, it is notable that for the LA/PV junction, all deficient conditions, including heterozygous G490R (*f* = 0.5) and homozygous G490R (*f* = 1.0) mutations, produced an increase in VW width. These observations suggest that the LA/PV junction (but not CT/PM junction) constitutes a location for increased vulnerability to unidirectional block (and hence reentry) with I_CaL_ reduction.

***Loss of rate-adaptation of atrial excitation***. Our data have shown that ${\text{I}}_{\text{CaL}}$ deficiency caused loss of rate-adaptation of atrial excitations, which were manifested by the flattened restitution curves of APD with decreased maximal slopes. Loss of rate-adaptation of atrial excitation has been observed in previous electrophysiological studies, and is believed to be arrhythmogenic [24,28]. Our results also showed a negative maximal slope of the APDR curves, representing the supernormal excitability of atrial tissue favoring the conduction of atrial excitation at high rates that are close to AF and normally cannot be conducted in WT tissue. Such an observation was also supported by the measured CV restitution curves, in showing that whilst at slow pacing rates (i.e., large BCLs), the measured conduction velocities in ${\text{I}}_{\text{CaL}}$ deficient tissues were almost the same as in WT tissue, but at high pacing rates (i.e., small BCLs), the measured CV was greater in ${\text{I}}_{\text{CaL}}$ deficient tissue, demonstrating a role in facilitating rapid atrial excitation waves seen in AF. In addition, the measured tissue excitation threshold also suggested increased tissue excitability at high pacing rates in markedly ${\text{I}}_{\text{CaL}}$ deficient tissue. Interestingly, different effects of ${\text{I}}_{\text{CaL}}$ deficiency on tissue excitability at typical SIs were observed from our simulations depending on the extent of current reduction: whilst tissue excitability was reduced in mildly/moderately ${\text{I}}_{\text{CaL}}$ deficient conditions, it was increased for larger ${\text{I}}_{\text{CaL}}$ reduction (f ≥ 0.5), including heterozygous and homozygous G490R mutations.

***APD dispersion over 3D human atria***. Our 3D simulation results showed an increased normalized global APD dispersion with high degrees of ${\text{I}}_{\text{CaL}}$ deficiency. The measured APD dispersion (ΔAPD) across the 3D atria was normalized by the mid-range of ${\text{APD}}_{\text{90}}$ across the entire atria. The normalized ${\Delta \text{APD}}_{\text{90}}$ increased from 0.44 in WT to 0.66 for the homozygous G490R mutation condition. Such increased APD dispersion are pro-arrhythmic [28]. However, in intermediate ${\text{I}}_{\text{CaL}}$ deficient tissue, a modest decrease in the normalized APD dispersion was observed, consistent with the trend of measured width of VW in the 1D heterogeneous strand model (CT/PM junction).

***Mechanism of sustained re-entry***. Pro-arrhythmic effects of deficient I_CaL_ can also be reflected by its effect on the maintenance of initiated reentries (i.e., the lifespan) in the tissue. Simulations of re-entry using 2D and 3D tissue models showed that for mildly ${\text{I}}_{\text{CaL}}$ deficient tissue (i.e., conditions with small *f* = 0.4) reentry self-terminated, which was attributable to a decreased tissue excitability. However, there was increased maintenance of reentry in the setting of more substantial ${\text{I}}_{\text{CaL}}$ reduction. This is attributable to the abbreviated APD that led to a shortened wavelength of excitation waves. As such, the atrial tissue would have sufficient substrate size to accommodate single or multiple reentrant excitation wavelets, which is pro-arrhythmic [28].

***Therapeutic strategies for Short QT syndrome.*** Treatments are primarily aimed at mitigating arrhythmia risk through a combination of pharmacological and interventional approaches. Quinidine, a Class IA antiarrhythmic agent, has been established as a cornerstone of pharmacological management of SQTS due to its efficacy in prolonging the QT interval and reducing the incidence of arrhythmic events [58–60]. Genotype specific treatment with I_Kr_ blocking Class I antiarrhythmic drugs may be possible for SQT1 variants in which impairment of hERG channel inactivation attenuates efficacy of Class III but not Class I drugs [31]. For patients deemed at high risk of sudden cardiac death, implantable cardioverter-defibrillators (ICDs) are frequently recommended; however, their use is complicated by the potential for inappropriate shocks due to the unique electrophysiological properties of SQTS. Despite these advancements, the management of SQTS remains challenging due to its rarity and phenotypic variability, highlighting the desirability for further research to optimize individualized treatment strategies and clinical outcomes.

### 4.1 Relevance and significance of the study

Previous studies have revealed pro-arrhythmogenic mechanisms of gain-of-function of ${\text{I}}_{\text{CaL}}$ mutations in (e.g., Timothy syndrome/long QT variant 8 (LQT8)) [61,62]. However, limited investigations on the impacts of loss-of-function on LTCC on cardiac electrical pro-arrhythmic effects have been made [63,64], and possible mechanisms underlying the pro-arrhythmic effects of reduced L-type calcium channel function in the human atria have not been fully elucidated. In this study, we have shown that while reducing I_CaL_ abbreviated atrial APD monotonically, it had biphasic effects on modulating atrial excitability and tissue susceptibility to initiation and maintenance of re-entry. Our simulations identify the PV/LA junction as a potential site for arrhythmia initiation in the setting of I_CaL_ reduction. The observation of biphasic effects of I_CaL_ reduction on the stationarity (i.e., meandering area) and the lifespan of reentrant excitation waves in 3D atrial tissue models is significant, as it indicated a bi-phasic modulation of deficient I_CaL_ on atrial tissue ability to sustain re-entry, which plays an important role for the transition of paroxysmal to persistent atrial fibrillation. In the 3D model with atrial anatomical structure and electrical heterogeneity [28], a substantial reduction in LTCC also increased spatial dispersion of APD, which is consistent with previous studies of other variants of SQTs [28]. However, new observations in this study differ from the functional effects of SQT1–3 K^+^ channel mutations on atrial excitation: there was no linear or monophasic correlation between the decrease in the maximal conductance of LTCC and changes in tissue excitability or susceptibility to genesis of uni-directional conduction block in response to a premature stimulus (though there was a positive correlation between abbreviations of ${\text{APD}}_{\text{90}}$, ERP and ${\text{I}}_{\text{CaL}}$ reduction). In this study, we found that the impact of the ${\text{I}}_{\text{CaL}}$ reduction on pro-arrhythmic effects is biphasic, in terms of the initiation and maintenance of re-entry, which differs to those seen in previous studies [23–30,65]. This finding extends understanding of the functional role of a reduced I_CaL_ arising either from loss-of-function of LTCC mutations or from pharmacological agents in pro- or anti- arrhythmogenesis, which is associated with the degree of I_CaL_ deficiency.

### 4.2 Limitations

In this study, there are some potential limitations that need to be addressed in future studies. The model exhibits certain limitations, particularly regarding the representation of human atrial action potentials in the basal models (i.e., the CZ/CRN) as they incorporate some electrophysiological data from canine atrial cells for some ion channel models in instances where data from human atrial cells were not available. Additionally, the 3D model used lacks a detailed tissue fibre structure [28]. These limitations have been extensively discussed in previous studies [24,39,66]. In the 3D simulations, we implemented the phase distribution method rather than tachypacing method, the latter of which is more physiologically relevant to ectopic foci [67].

It must be noted that in this study we adopted a simplified approach of reducing the maximal macroscopic conductance of L-type calcium current for simulating the effect of the G490R and A39V mutations on the reduction of I_CaL_ amplitude, without considering other possible changes in the channel kinetics of I_CaL_ by the mutations due to the lack of experimental data. However, as pointed out by Antzelevitch et al. [5] in their original study, there was no observed change in the voltage at which I_CaL_ peak current occurred in WT and mutation conditions, which implied no major change in the voltage-dependent kinetics of the current. This supports the appropriateness of our use of a simplified approach to simulate the G490R and A39V mutations. It is warranted in future to consider possible changes in the rate of activation and inactivation process when more data become available. Further potential limitations are imposed by the lack of recombinant channel data for mimicking the heterozygous state of mutation condition as seen in the patients [5] as experimental data were only available in the WT and mutant Cav1.2 conditions. In this study, we endeavored to circumvent this limitation by conducting simulations using a range of progressive reductions of I_CaL_ magnitude. It was found that, at *f* = 0.5 (mimicking the heterozygous mutation condition), the reduction in I_CaL_ abbreviated atrial APD and ERP, reduced the excitation threshold at the critical SI, and increased the VW at the LA/PV junction, which are pro-arrhythmic. However, the overall pro-arrhythmic effect sometimes occurred at a higher degree of I_CaL_ deficiency (f ≥ 0.6). This highlights the importance of ascertaining the precise extent of loss of I_CaL_ in patients heterozygous for *CACN1AC* mutations. In this study, we simulated the I_CaL_ reduction in the heterozygous mutation condition by simply combining I_CaL_ in WT and MT conditions together (WT:MT = 50%:50%). It remains to be established whether such an assumption holds, or if it might under- or over-represent the extent of I_CaL_ reduction in patients. Therefore, further improvements of the mutant I_CaL_ formulations may be possible in the future when more experimental data become available.

Additionally, the CRN/CZ model used here was simplified for computation and lacked some electrophysiological details. For example, this CRN model only took fast transient sodium current into consideration [39] and ignored any persistent, late sodium current component. Also cardiac mechanical contraction was not considered, which might affect calcium channel and calcium dynamics and overall electrophysiology. That said, currently, no direct evidence highlights critical disadvantages of utilizing CRN/CZ model to investigation human atrial electrophysiology. Therefore, whilst we make the limitations of the study explicit, it is unlikely that these limitations fundamentally affect our conclusions on the arrhythmogenic effects of deficient I_CaL_ associated with the Brugada/abbreviated QT interval phenotype mutations [5] studied here.

## 5. Conclusions

Our results showed that a decreased ${\text{I}}_{\text{CaL}}$ and its secondary effects on other ion channel currents accelerated atrial repolarisation, leading to shortened APD and ERP, as well as the loss of their rate-dependence. At the tissue level, markedly decreased ${\text{I}}_{\text{CaL}}$ shortened the wavelength of atrial excitation waves. Though it slowed down mildly atrial conduction velocity at low pacing rates, it increased it greatly at high pacing rates. A markedly deficient ${\text{I}}_{\text{CaL}}$ (*f* ≥0.6; including homozygous mutation) increased tissue susceptibility to initiation and maintenance of atrial re-entrant excitation waves. For a moderate ${\text{I}}_{\text{CaL}}$deficiency (*f* = 0.5; the simulated heterozygous mutation condition), some pro-arrhythmic effects (e.g., APD and ERP abbreviation; increased VW at the LV/PVJ) were shown. However, smaller extents of ${\text{I}}_{\text{CaL}}$deficiency (f ≤ 0.4), were associated with opposite effects. In conclusion, this study provides mechanistic insights into the complex effect of ${\text{I}}_{\text{CaL}}$ deficiency on arrhythmogenesis and maintenance, depending on the degree of ${\text{I}}_{\text{CaL}}$ deficiency that is present.

## Supporting information

S1 Text Supplementary information about simulation methods, model validation, model-dependence tests and supplementary investigations. (DOCX)

S1 Fig Comparison of I-V curves. Experimental data (dots) from patch-clamp recordings are compared with simulations (line) generated by the modified CRN model. Model parameters are provided in S1 Table.
(TIFF)

S2 Fig Schematic of the 1D human atrial tissue model and the protocols for electrophysiological measurements. The model represents a strand of tissue composed of 100 isotropic nodes (spatial resolution: 0.25 mm). Conduction velocity was determined by recording the activation time difference between the 25th and 75th nodes. The effective refractory period (ERP) and excitability threshold (EXT) were assessed using an S1-S2 pacing protocol.
(TIFF)

S3 Fig Schematic diagram depicting the cross-field S1-S2 protocol in 2D simulations. S1 stimuli were applied at the lower edge of the 2D sheet, while the S2 stimulus was applied at the lower-left region of the sheet, occupying one-fourth of the total area of the 2D sheet, which consists of 200x200 nodes. The interaction between the S1 and S2-evoked excitation waves led to the formation of reentrant excitation waves.
(TIFF)

S4 Fig (A). Schematic illustration of variant distinct anatomical regions of the atria, annotated with corresponding anatomical labels. (B). Spatiotemporal initiation of 3D reentry in the human atrial model via phase distribution. The tissue was initialised by assigning 206 discrete phases, each of which corresponding to a phase state of an action potential of a single right atrial cell (right panel), which was mapped to the 3D spatial region of the left atrium, creating heterogeneous refractoriness. Red regions (positive membrane potential close to 10 mV) denote depolarised/active tissue, while blue regions (resting potential ≤ -80 mV) represent fully repolarised to rest substrate, while the spectrum from red to blue represents the intermediate depolarised region with membrane potential changing from -80 to +10 mV (discretised by 206 phases; see the colour. (C). Illustration of the location within the 3D atrial geometry at which the pseudo-ECG was computed.key).
(DOCX)

S5 FigIonic currents.Computed APs and related ion channel currents in WT, homozygous(*f* = 1), heterozygous(f = 0.5) A39V mutations, and intermediate ${\text{I}}_{\text{CaL}}$ deficiency (*f* = 0.2, 0.4, 0.6 and 0.8) conditions. (i) APs. (ii) ${\text{I}}_{\text{CaL}}$. (iii) ${\text{I}}_{\text{Na}}$. (iv) ${\text{I}}_{\text{Kr}}$. (v) ${\text{I}}_{\text{NaCa}}$. (vi) ${\text{I}}_{\text{K1}}$.(TIFF)

S6 FigRate-dependent changes in action potential properties at the single-cell level.Calculated restitution curves of APD in WT, homozygous A39V mutation (*f* = 1) and ${\text{I}}_{\text{CaL}}$ deficiency (*f* = 0.2, 0.4, 0.5, 0.6 and 0.8) conditions. (i) APD restitution (APDr) curve. (ii) The maximal slope of the APDr curves. (iii) ERP restitution (ERPr) curves. (iv) APD and ERP versus various deficient scaling factor *f*.(TIFF)

S7 Fig Characteristics of APs for WT, the homozygous A39V(exon 8), A39V, and G490R mutation conditions at the single-cell level. (TIFF)

S8 Fig Key ionic currents underlying action potential morphology in WT versus homozygous mutant conditions with the Grandi et al. model. (TIFF)

S9 FigTemporal vulnerability windows in atrial tissue under WT and A39V-associated ${\text{I}}_{\text{CaL}}$ deficiency.Panels A and B show the computed VWs at the CT/PM and LA/PV junctions, respectively. Panel C displays a biphasic change in VW width at the CT/PM junction with the scaling factor *f*. In contrast, at the LA/PV junction (Panel D), the VW width increased monotonically with f, a trend consistent with that observed in G490R-associated cases.(TIFF)

S10 FigDynamics of re-entrant spiral waves under WT, intermediate ${\text{I}}_{\text{CaL}}$ deficiency, and homozygous A39V mutation conditions.For each condition, WT (A), intermediate ${\text{I}}_{\text{CaL}}$ deficiency (B-F) and homozygous *CACNA1C* A39V mutation (G) conditions. Snapshots of reentry (e.g., Ai to Aiii), tip trace pattern (e.g., Aiv), time series of integrated transmembrane potential across the entire 2D tissue (e.g., Av), and its power spectrum (e.g., Avi) are shown. The lifespan of reentries is documented in Fig S10(H). Fig S10(I) presents the meandering area of core trajectory for the wild-type and A39V-associated ${\text{I}}_{\text{CaL}}$ deficient conditions.(TIFF)

S11 FigProperties of simulated scroll waves in 3D models.(A) Bar charts of ${\text{APD}}_{\text{90}}$ across the whole 3D human atrial tissue in the WT, A39V mutation, and corresponding deficient ${\text{I}}_{\text{CaL}}$ conditions. (B) The lifespan of re-entrant scroll waves in WT, A39V mutation, and intermediate deficient ${\text{I}}_{\text{CaL}}$ conditions. (C) Normalized APD dispersion: $\Delta \text{AP}{\text{D}}_{\text{90}}$versus deficient scaling factor *f*. (D) The lifespan of 3D re-entrant scroll versus deficient coefficient *f*.(TIFF)

S12 Fig Three-dimensional simulations. (A) Snapshots of scroll waves at different timings (labelled on top of the figure) from 3D model simulations for WT, homozygous CACNA1C A39V mutation, and its corresponding intermediate deficient conditions. The action potential was coloured coded from -100 mV in blue to 20 mV in dark red, with the colour key shown at the bottom. (B) Colour mapping of APD dispersion in 3D human atria. APD dispersion was colour coded from 75 ms in blue to 269 ms in dark red. Colour key is shown at the bottom. (C) Time series of integrated action potential over the whole 3D human atrial space.
(TIFF)

S13 Fig One-dimensional simulations for WT and all homozygous mutations. Restitution curves of conduction velocity (A), effective refractory period (B), and wavelength (C) of excitation waves for all homozygous mutations versus varying BCLs. Tissue refractoriness is determined via restitution curves of excitation threshold versus S1-S2 interval, as shown in panel (D).
(TIFF)

S14 Fig Temporal vulnerability windows for WT and all homozygous mutations. Measured temporal vulnerability windows at the CT/PM junction (A) and LA/PV junction (B). All homozygous mutations led to increased VW widths at both CT/PM and LA/PV junctions.
(TIFF)

S15 Fig Two-dimensional simulations for WT and all homozygous mutations. Snapshots of spiral waves in the 2D substrate model, panel i, ii and iii representing spiral waves at time equal to 1000, 5000 and 8000 ms respectively. Panel iv represented the trajectories of the core of re-entrant excitations. Panel v represents the time series of integrated transmembrane potential across the entire 2D sheet. Panel vi represents the corresponding power spectrum of the time series.
(TIFF)

S16 Fig Dynamics of re-entrant excitation in a 3D virtual human atrium for WT and all homozygous mutations. Snapshots of re-entrant excitation waves at three different time points (panel i-iii). The spatial distribution of APD_90_ is displayed in panel iV. Time series of integrated membrane potential across 3D tissue are given in panel V.
(TIFF)

S17 FigAction potential duration and its dispersion in 3D human atria under WT and mutant conditions.(A). Global ${APD}_{\text{9}\text{0}}$ in 3D virtual human atria for WT and all mutation conditions. (B). Normalized $\Delta {APD}_{\text{9}\text{0}}$ for WT, A39V (exon 8), A39V, G490R mutations. APD dispersion ${\Delta APD}_{\text{9}\text{0}}$ was normalized by midrange of APD across 3D atria in each case. The normalized APD dispersion was increased in all mutation conditions, compared to that in WT.(TIFF)

S1 Table I_CaL_ formulation parameters. Parameters of modified CRN model to match the simulated I-V curves to the experimental data presented by Antzelevitch et al.
(DOCX)

S2 Table Scaling factor of gCaL for WT and homozygous mutation cases. (DOCX)

S3 Table Regional scaling coefficients of maximal ionic conductance. List of electrophysiological scaling coefficients (GX) modulating maximal conductance of ionic current compared to the reference right atrial (RA) cellular framework, with associated reference studies. For each current listed a value of 1 was allocated to its value in RA cells and the ratios shown are relative to this. Key abbreviations: CT (crista terminalis), BB (Bachmann’s bundle), PM (pectinate muscles), AVR (atrioventricular ring), RAA (right atrial appendage), AS (atrial septal region), LA (left atrial chamber), LAA (left atrial appendage), PV (pulmonary venous tissue).
(DOCX)

S4 TableAction potential characteristics of deficient I_CaL_ conditions linked to the A39V mutation.Effects of deficient ${\text{I}}_{\text{CaL}}$ conditions linked to the A39V mutation on AP characteristics. The homozygous (*f* = 1), heterozygous (f = 0.5) A39V mutation conditions, and intermediate ${\text{I}}_{\text{CaL}}$ deficiency (*f* = 0.2, 0.4, 0.6 and 0.8) resulted in an increase in both the amplitude of the action potential and the maximal upstroke velocity, as compared to the WT. Additionally, ${\text{I}}_{\text{CaL}}$ deficiency caused a more negative resting membrane potential and a shorter duration of the action potential.(DOCX)

S5 Table Characteristics of APs in WT and all homozygous mutation conditions. (DOCX)

S6 TableTwo-dimensional simulation results for A39V corresponding deficient ${\text{I}}_{\text{CaL}}$ conditions.Lifespans of reentries and computed dominant frequency of time series, and spiral wave core movement area of re-entrant excitations in WT, CACNA1C A39V mutation and corresponding deficient ${\text{I}}_{\text{CaL}}$ conditions in the 2D model.(DOCX)

S7 TableThree-dimensional simulation results for A39V corresponding deficient ${\text{I}}_{\text{CaL}}$ conditions.Lifespan and computed dominant frequency of scroll waves in WT, CACNA1C A39V mutation, and corresponding deficient ${\text{I}}_{\text{CaL}}$ conditions within the 3D model.(DOCX)

S1 Video Videos of reentrant excitation waves in 2D tissue models (WT). (MP4)

S2 Video Videos of reentrant excitation waves in 2D tissue models (*f* = 0.2). (MP4)

S3 Video Videos of reentrant excitation waves in 2D tissue models (*f* = 0.4). (MP4)

S4 Video Videos of reentrant excitation waves in 2D tissue models (*f* = 0.5). (MP4)

S5 Video Videos of reentrant excitation waves in 2D tissue models (*f* = 0.6). (MP4)

S6 Video Videos of reentrant excitation waves in 2D tissue models (*f* = 0.8). (MP4)

S7 Video Videos of reentrant excitation waves in 2D tissue models (homozygous G490R mutation). (MP4)

S8 Video Videos of reentrant excitation waves in 3D models (WT). (MP4)

S9 Video Videos of reentrant excitation waves in 3D models (*f* = 0.2). (MP4)

S10 Video Videos of reentrant excitation waves in 3D models (*f* = 0.4). (MP4)

S11 Video Videos of reentrant excitation waves in 3D models (*f* = 0.5). (MP4)

S12 Video Videos of reentrant excitation waves in 3D models (*f* = 0.6). (MP4)

S13 Video Videos of reentrant excitation waves in 3D models (*f* = 0.8). (MP4)

S14 Video Videos of reentrant excitation waves in 3D models (homozygous G490R mutation). (MP4)
