# Supplementary material for: Biphasic effects on human atrial arrhythmogenicity of L-type calcium channel mutations associated with a Brugada/Short QT overlap syndrome - insights from a multiscale simulation study
Source: PLoS Comput Biol. 2025 Nov 19;21(11):e1013616. doi: 10.1371/journal.pcbi.1013616 (PMC12629484; doi:10.1371/journal.pcbi.1013616)
Supplement: S1 Text — (DOCX) [file pcbi.1013616.s001.docx]

*Online supplementary information*

to

Biphasic effects of on human atrial arrhythmogenicity of L-type calcium channel mutations associated with a Brugada/Short QT overlap syndrome - insights from a multiscale simulation study

Yirong Xiang^1^, Jules C. Hancox^1,2^, Henggui Zhang^1,3^*

^1^Biological Physics Group, Department of Physics and Astronomy,

The University of Manchester, UK

^2^ School of Physiology, Pharmacology and Neuroscience, Biomedical Sciences Building, University Walk, Bristol, BS8 1TD, UK

^3^State Key Laboratory of Digital Medical Engineering, School of Instrument Science and Engineering, Southeast University, Nanjing, China

*Correspondence: henggui.zhang@manchester.ac.uk

# Supplementary Materials

1. **Basal Model of** $\boldsymbol{I}_{\boldsymbol{CaL}}$

The CRN model of $I_{\mathrm{CaL}}$ used in human atrial simulations are given in following equations:

$$\begin{aligned} I_{CaL}=g_{CaL}\cdot d\cdot h\cdot h_{Ca}\cdot\left( V_{m}-E_{r} \right),\#(1-1)\# \end{aligned}$$

$$\begin{aligned} \frac{dd}{dt}=\frac{d-d_{\infty}}{\tau_{d}}\#\left( 1-2 \right) \end{aligned}$$

$$\begin{aligned} \frac{dh}{dt}=\frac{h-h_{\infty}}{\tau_{h}},\#(1-3)\# \end{aligned}$$

$$\begin{aligned} \frac{dh_{Ca}}{dt}=\frac{h_{Ca}-h_{Ca(\infty)}}{\tau_{h(Ca)}},\#\left( 1-4 \right) \end{aligned}$$

$$\begin{aligned} d_{\infty}=\frac{1.0}{1.0+\exp\left( \frac{V_{m}+10.0}{-8.0} \right)},\#\left( 1-5 \right) \\ \#\# \end{aligned}$$

$$\begin{aligned} h_{\infty}=\frac{1.0}{1.0+\exp\left( \frac{V_{m}+28.0}{6.9} \right)},\#\left( 1-6 \right)\#\# \end{aligned}$$

$$\begin{aligned} h_{Ca\left( \infty\right)}=\frac{1.0}{1.0+\frac{\left[ {Ca}^{2+} \right]_{i}}{0.00035}}, \#\left( 1-7 \right) \end{aligned}$$

$$\begin{aligned} \tau_{d}=\frac{1.0-\exp\left( -\frac{V_{m}+10.0}{6.24} \right)}{0.035\cdot(V_{m}+10.0)(1.0+\exp\left( -\frac{V_{m}+10.0}{6.24} \right))},\#\left( 1-8 \right)\#\# \end{aligned}$$

$$\begin{aligned} \tau_{h}=\frac{9\cdot1.0}{0.0197\cdot\exp\left( -{0.0337}^{2}\cdot\left( V_{m}+10.0 \right)^{2} \right)+0.02},\#\left( 1-9 \right)\#\# \end{aligned}$$

$$\begin{aligned} \tau_{h\left( Ca \right)}=2,\#\left( 1-10 \right) \end{aligned}$$

where $g_{CaL}$ is the maximum channel conductance, $V_{m}$ the transmembrane potential; *d, h* and $h_{Ca}$ are the voltage dependent activation, inactivation and intracellular calcium dependent inactivation variable respectively. And $E_{r}$ is the reversal potential of the L-type calcium channel current. $\tau_{d}$, $\tau_{h}$ and $\tau_{h(Ca)}$ are the time constants of three gate variables of *d, h* and $h_{Ca}$ respectively. And ${[{Ca}^{2+}]}_{i}$ is intracellular calcium concentration (1).

1. **Modified parameters in CRN Model to incorporate Experimental Data**

Activation, inactivation variable in Equation (1) were updated to incorporate the experimental data, Equation (1-5), (1-6) ,(1-8) (1-9) are modified in following equations:

$$\begin{aligned} d_{\infty}=\frac{1.0}{1.0+exp(\frac{V_{m}+V_{d\frac{1}{2}}+10.0}{-8.0\cdot{grad}_{d}})},\#(2-1)\#\# \end{aligned}$$

$$\begin{aligned} h_{\infty}=\frac{1.0}{1.0+\exp\left( \frac{V_{m}+V_{h\frac{1}{2}}+28.0}{6.9\cdot{grad}_{h}} \right)},\#(2-2)\#\# \end{aligned}$$

$$\begin{aligned} \tau_{d}=\frac{1.0-\exp\left( -\frac{V_{m}+10.0+V_{d\frac{1}{2}}}{6.24} \right)}{0.035\cdot(V_{m}+10.0+V_{d\frac{1}{2}})(1.0+\exp\left( -\frac{V_{m}+10.0+V_{d\frac{1}{2}}}{6.24} \right))},\#\left( 2-3 \right) \end{aligned}$$

$$\begin{aligned} \tau_{h}=\frac{9\cdot1.0}{0.0197\cdot\exp\left( -{0.0337}^{2}\cdot\left( V_{m}+10.0+V_{h\frac{1}{2}} \right)^{2} \right)+0.02},\#\left( 2-4 \right) \end{aligned}$$

where $V_{d\frac{1}{2}}$ and $V_{h\frac{1}{2}}$ are the shift of voltage for $V_{\frac{1}{2}}$ of the steady-state activation (*d)* and inactivation *(h)* curves caused by the gene mutation, and ${grad}_{d}$ and ${grad}_{h}$ are their corresponding slopes. In the basal CRN model, $V_{d\frac{1}{2}}$ and $V_{h\frac{1}{2}}$ equal to 0 and ${grad}_{d}$, ${grad}_{h}$ equal to 1. The basal CRN model of $I_{CaL}$ was modified with considering alterations of activation/inactivation variables, $V_{d\frac{1}{2}}$ , $V_{h\frac{1}{2}}$ , ${grad}_{d}$, and ${grad}_{h}$ to fit experimental I-V curves of patch-clamp protocols (2). In addition, the maximal macroscopic channel conductance of $I_{CaL}$ was reduced to match a significant decrease of the peak value of the I-V curve in the mutation conditions as compared to the WT condition. Fitting the simulation data of the $I_{CaL}$ model was done via an optimization method based on Nelder-Mead algorithm (3). The obtained parameters for the modified CRN model to fit experimental patch-clamp measurements of Antzelevitch et al. (2) are listed in Table S1.

**Table S1.** **I_CaL_ formulation parameters.**

| **Model component** | | **Exon 8A** | | | **Exon 8** | | |
| --- | --- | --- | --- | --- | --- | --- | --- |
|  |  | **WT** | **A39V** | **G490R** | **WT** | **A39V** | **G490R** |
| Activation | $V_{d\frac{1}{2}} (mV)$ | -18.0 | - 14.3 | -18.0 | -12.6 | -11.0 | -25.9 |
|  | ${grad}_{d}$ | 0.8 | 0.8 | $\text{1}\text{.}\text{0}$ | 1.0 | 0.9 | 1.5 |
| Inactivation | $V_{h\frac{1}{2}} (mV)$ | 40.1 | 40.1 | 49.8 | 50.7 | 50.7 | 50.7 |
|  | ${grad}_{h}$ | 1.5 | 1.5 | 1.5 | 0.6 | 1.1 | 0.7 |
| Scaling factor of the maximum channel conductance (g_CaL_) relative to that in the basal model | | × 1.39 | $\times$ 0.18 | $\times$ 0.10 | $\times$ 1.73 | $\times$ 0.35 | $\times$ 0.12 |

Parameters of modified CRN model to match the simulated I-V curves to the experimental data presented by Antzelevitch et al. (2).

Fig S1 presents the comparison of the experimental data of the I-V relationship from the study of Antzelevitch et al.(2) and that from the modified CRN model with parameters listed in Table S1. As shown in the figure, the modified CRN model reproduced the experimentally observed I-V curves in WT and mutation conditions, by which I_CaL_ was reduced. Note that it was necessary to implement a scaling factor for the maximal channel conductance of the basal I_CaL_ to allow the $I_{CaL}$ peak value in the I-V curve to match the basal CRN model for human atria in WT. However, when the modified WT I_CaL_ equation was incorporated in the basal CRN model, the simulated action potentials had a duration at 90% repolarization significantly shorter than that of the basal CRN model for a healthy human atrial cell. As the experimental data of Antzelevitch et al. (2) were obtained from Chinese hamster ovary (CHO) cells at room temperature, which was different from the native human atrial cells at physiological temperature, in the following simulations the basal CRN I_CaL_ model was used for the WT condition.

**Fig S1. Comparison of I-V curves.​** Experimental data (dots) from patch-clamp recordings are compared with simulations (line) generated by the modified CRN model. Model parameters are provided in S1 Table.

1. **Implemented CRN Model with reduced maximum conductance of** $\boldsymbol{I}_{\boldsymbol{CaL}}$

Experimental data of Antzelevitch et al. (2) showed that the G490R/A39V mutations decreased L-type calcium channel current densities with no significant shift in the voltage-dependent I-V curves. In simulations, a scaling factor of the maximal conductance $g_{CaL}$ for the mutation conditions in relative to that in WT was used. To match the I_CaL_ reduction to that observed experimentally, the scaling factor for g_CaL_ was re-normalised to WT and set to 0.07, 0.13 and 0.20 respectively for the homogeneous G490R, A39V, and A39V (Exon 8) mutations (shown in Table S2), based on the optimization results shown in Table S1. Since the scaling factors for g_CaL_ in G490R (exon 8A) and G490R (exon 8) mutations were almost identical, in simulations, the G490R mutation was studied without distinguishing exon 8A and exon 8 conditions.

To simulate a general condition of deficient $I_{CaL}$ including heterozygous mutations, we considered I_CaL_ with two mixed components of WT ($I_{CaL}^{WT})$and mutation ($I_{CaL}^{mut}),$with a proportion factor of $f$ changing from 0 to 1. Therefore, a general deficient ${I'}_{CaL}$ was expressed as:

$$\begin{aligned} I_{CaL}^{'}=(1-f)\cdot I_{CaL}^{WT}+f\cdot I_{CaL}^{mut},\#\left( 3 \right) \end{aligned}$$

where $I_{CaL}^{WT}$ and $I_{CaL}^{mut}$ are $I_{CaL}$ for WT and the homozygous mutation. In simulations, f=0 represented the WT condition, f=0.5 and 1 represented heterozygous and homozygous mutation respectively. An intermediate $g_{CaL}$ deficiency was represented by 0 < f <1.

**Table S2. Scaling factor of gCaL for WT and homozygous mutation cases.**

| WT/MT | WT | G490R | A39V | A39V (exon 8) |
| --- | --- | --- | --- | --- |
| Relative to $g_{CaL}$ | 1 | 0.07 | 0.13 | 0.20 |

1. **Multi-dimensional simulations of deficient** $\boldsymbol{I}_{\boldsymbol{CaL}}$ **in the human atria**

# Single-cell model and AP simulations

At the single-cell level, APs were evoked by applying a series of supra-threshold external stimuli to the CRN model with varied basic cycle lengths (BCLs). To measure the characteristics of APs, the model was first stimulated by applying 100 external conditioning stimuli (S1) to stabilize the model. The final AP was registered for analysis. To measure the restitution properties of the model, the standard S1-S2 protocol was utilized. After a time interval to the final stimulus of the S1 stimuli (SI; also labeled as the S1-S2 interval), an additional external stimulus (S2) was applied to the model to measure the characteristics of the S2-evoked AP, which include the action potential duration at 90% repolarization (${APD}_{90}$), AP amplitude (APA), the maximum upstroke velocity (MUV), the resting membrane potential (RMP) and the effective refractory period (ERP) of atrial cells in WT and mutation conditions.

***APD restitution curves***: the restitution curves of the APD and EPR were computed and constructed using the single-cell model. In simulations, the standard S1-S2 stimulation protocol described above was used with varied S1-S2 intervals. In response to the S2 stimulus, the evoked AP was registered and the APD_90_ was computed. The relationship between action potential duration and diastolic interval (DI) was subsequently depicted as APDr curves.

***ERP restitution curves***: The EPRr curves were established through the application of varying basic cycle lengths (BCLs) of S1 stimuli in our simulations. For each specific BCL, a range of S1-S2 intervals was imposed at the single-cell level. The smallest S1-S2 interval that enabled the subsequent initiated action potential to attain 80% of the amplitude of the preceding action potential was identified as the ERP for that particular BCL in the corresponding cardiac myocytes. The relationship between the measured ERP and BCL was subsequently depicted as ERPr plots.

# One-dimensional tissue strand model

A one-dimensional strand model was created utilizing 100 isotropic single cells configured in a linear alignment with a spacing of 0.25 mm as shown in Fig S2. The 1D strand tissue model was used to characterize the conduction velocity (CV), effective refractory period (ERP), and wavelength (WL) of excitation waves at different BCLs. In addition, the excitation threshold (EXT), defined as the minimal amplitude of a stimulus strength to evoke propagation of excitation waves in a one-dimensional strand tissue model, was quantitatively evaluated at varying S1-S2 intervals in order to investigate tissue excitability.

***Measurement of conduction velocity and CVr***. Conduction Velocity (CV) was calculated by dividing the distance of excitation wave propagation by the corresponding propagation time. In our one-dimensional simulations, we set the initiation and termination nodes for recording propagation at the 25th and 75th nodes, respectively. We induced excitation at one end of the 1D tissue using fixed-strength stimuli (20 pA/pF) with a standardized duration of 2.0 ms. The conduction velocities were measured across a range of Basic Cycle Lengths from 200 ms to 1000 ms, and were used to construct CV restitution curves (CVr).

***Measurement of the effective refractory period (ERP), ERP restitution (ERPr), wavelength (WL) and wavelength restitution (WLr)***. The Effective Refractory Period (ERP) is the shortest S1-S2 interval that allows excitation wave propagation to ¾ of the total strand length. We calculated the Wavelength (WL) by multiplying the measured CV and ERP. Restitution curves for ERP and CV were derived by varying the BCL of different rates, following a methodology similar to that used for CVr.

***Measurement of Excitation threshold (EXT)***. In the simulations, we applied S1 stimuli at the start of the 1D strand model and the S2 stimulus at the midpoint. We measured the propagation of excitation to the terminals at both ends of the strand model. We defined EXT as the minimum S2 stimulus strength capable of inducing propagated action potentials at both ends of the 1D strand in our study.

***Measurement of vulnerability window (VW)***. In the 1D strand model, we measured the temporal vulnerable window (VW) to investigate tissue refractoriness under both WT and deficient $I_{\mathrm{CaL}}$ conditions. The temporal vulnerability window represents the time delay between the S2 stimulus and the last S1 stimulus, which occurs after the effective refractory period. Using the S1-S2 protocol, an action potential initiated by the S2 stimulus at the junction of the 1D model could encounter one of three scenarios. (i) The action potential (AP) can propagate in both directions if both regions have recovered (bi-directional conduction). (2) The AP will be blocked in both directions if all regions are in a refractory state (bi-directional block). (3) The AP can propagate in one direction in which the region has recovered and be blocked in the other direction where the region is still in a refractory state (uni-directional conduction block). The uni-directional conduction block can be quantitatively assessed by measuring the vulnerability window reciprocally. Spatially heterogeneous tissues were created in 1D simulations using a 1D CT/PM junction model and a 1D LA/PV junction model (4). The 1D model was first stabilized by applying multi-S1 stimuli at the beginning of the CT region. The action potential (AP) originating from CT then propagated to the PM region. Tissue refractoriness was assessed by applying an S2 stimulus to the junction of the 1D model after the S1-S2 interval. Measurements were taken at both ends of the 1D model to evaluate the propagation of the action potential (AP) elicited by the S2 stimulus. The threshold used to determine the propagation of the AP was -20 mV. The temporal vulnerability window was identified as the S1-S2 interval under which the AP initiated at the junction of the 1D model could only propagate in one direction. The width of the vulnerability window is the difference between the maximal and minimal stimulus time leading to a uni-directional conduction block. The AP of the 1D LA/PV junction model was initiated at the beginning of the LA region and propagated to the PV region. An S2 stimulus was then applied at the junction of LA/PV with various S1-S2 intervals to measure VW.


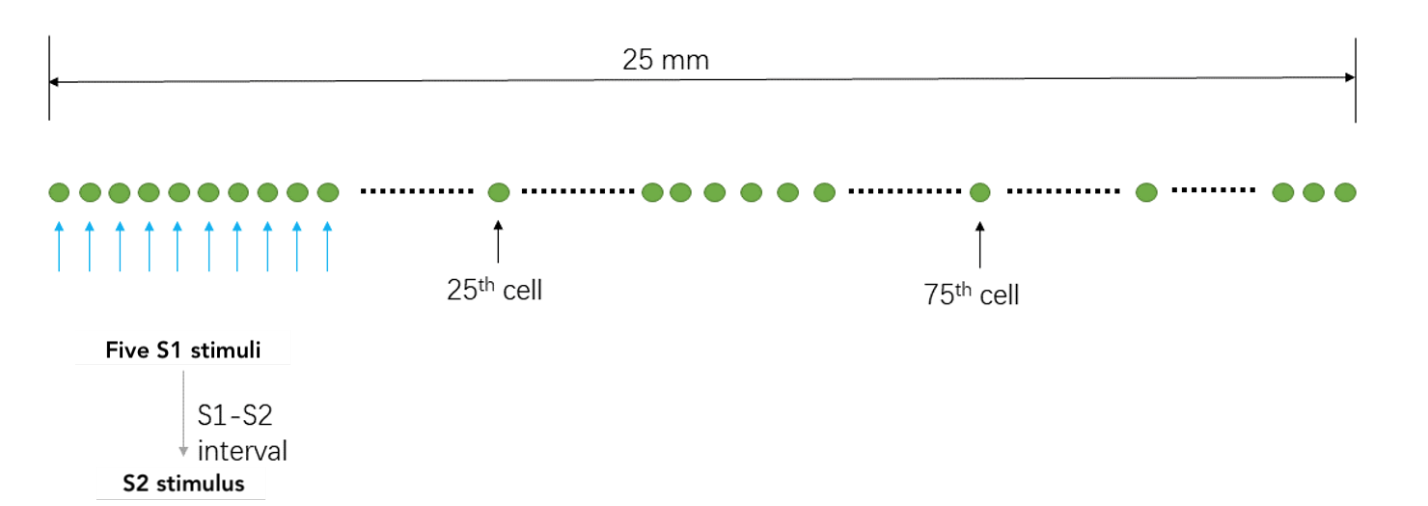


**​Fig S2. Schematic of the 1D human atrial tissue model and the protocols for electrophysiological measurements.​​** The model represents a strand of tissue composed of 100 isotropic nodes (spatial resolution: 0.25 mm). Conduction velocity (CV) was determined by recording the activation time difference between the 25th and 75th nodes. The effective refractory period (ERP) and excitability threshold (EXT) were assessed using an S1-S2 pacing protocol.

# Simulations in the Two-dimensional idealized sheet model

To investigate the dynamics of re-entry, a 2D substrate tissue model was employed, consisting of 400x400 nodes with a spacing of 0.25 mm. This 2D idealized sheet model had a surface area 10x10 ${cm}^{2}$, which is comparable in size to that of real human atria (5). In order to initialize spiral waves in the 2D sheet, a cross-field S1-S2 stimulus protocol was employed in simulations (6). Specifically, the S1 stimulus was applied at the bottom edge of the 2D sheet, and after a defined S1-S2 interval, the S2 stimulus was administrated to the lower left quadrant of the entire substrate (5x5 ${cm}^{2}$) of the 2D sheet, as demonstrated in Fig S3. The plane waves evoked by the last S1 stimulus interacted with excitations induced by the S2 stimulus, resulting in re-entrant excitations. In simulations, measurements were conducted for WT, mutations and $I_{CaL}$ deficient conditions, using different S1-S2 intervals (around its effective refractory period in each condition).

To investigate the dynamical behaviors of re-entries, membrane potential was visualized over the entire 2D sheet to monitor the evolution of spiral waves, with a particular focus on the trajectories of cores, using the method of phase singularities (7). Furthermore, to obtain the power spectrum of re-entrant excitations, Fourier transformations of the integrated membrane potential (the electrode being strategically positioned at (18.75, 30.0) mm over the entire 2D sheet were performed under both WT and deficient $I_{CaL}$ conditions, with the dominant frequency being identified (8).


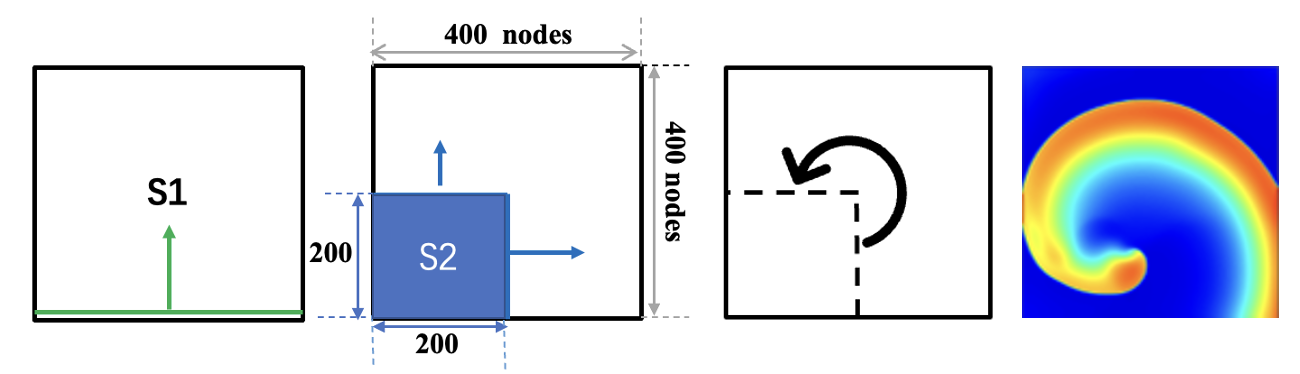


**Fig S3. Schematic diagram depicting the cross-field S1-S2 protocol in 2D simulations.** S1 stimuli were applied at the lower edge of the 2D sheet, while the S2 stimulus was applied at the lower-left region of the sheet, occupying one-fourth of the total area of the 2D sheet, which consists of 200x200 nodes. The interaction between the S1 and S2-evoked excitation waves led to the formation of reentrant excitation waves.

# Simulations in 3D anatomical human atrial model

A 3D virtual human atrial anatomical model was constructed utilizing the visible human cardiac dataset, built upon our previous studies (4, 6, 9, 10). In the 3D anatomical model, heterogeneous electrical properties of various regions (Fig S4(A)) within human atria were considered as previously described in detail，corresponding ion channel maximal conductance ratio relative to right atrial myocytes are presented in Table S3 (4). The (Colman-Zhang) CZ model was updated with a family of regional cell model which were described in the work of Whittaker et al (4). The spatial resolution of cells within anatomical atrial tissue is 0.33 × 0.33× 0.33 *mm^3^*, with a total of 1.6 million cells. In the model, we used a diffusion coefficient tensor to simulate the anisotropic spatial diffusion of excitation waves. Due to the absence of sheet structure, only two components of ***D***: along the direction of AP propagation and transverse to it were included in the 3D model. The component in the transverse direction, $D_{\perp}$, was set to 0.21 ${mm}^{2}/ms$, with a 9-fold increase of the component in the longitudinal direction (6). To solve the partial differential equation of the transmembrane potential, the finite difference method was used (11).

**Table S3. Regional scaling coefficients of maximal ionic conductance.**

|  | $G_{CaL}$ | $G_{to}$ | $G_{Kur}$ | $G_{Na}$ | $G_{Kr}$ | $G_{Ks}$ | $G_{K1}$ | Paper Source |
| --- | --- | --- | --- | --- | --- | --- | --- | --- |
| CT | 1.68 | 1.35 | 1.0 | 1.0 | 1.0 | 1.0 | 1.0 | (6, 12) |
| BB | 1.72 | 1.35 | 1.0 | 1.0 | 1.0 | 1.0 | 1.0 | (6, 12, 13) |
| PM | 0.94 | 1.0 | 1.0 | 1.0 | 1.0 | 1.0 | 1.0 | (6, 12) |
| AVR | 0.67 | 0.6 | 1.0 | 1.0 | 1.63 | 1.0 | 1.0 | (12) |
| RAA | 1.0 | 0.68 | 1.0 | 1.0 | 1.0 | 1.0 | 1.0 | (12, 14) |
| AS | 0.4 | 0.212 | 0.667 | 1.3 | 1.0 | 1.0 | 1.0 | (14) |
| LA | 1.0 | 1.0 | 1.0 | 1.0 | 1.6 | 1.0 | 1.0 | (12, 15, 16) |
| LAA | 1.0 | 0.53 | 0.8 | 1.0 | 1.6 | 1.0 | 1.0 | (12, 16, 17) |
| PV | 0.7 | 0.75 | 1.0 | 1.0 | 2.4 | 1.5 | 0.62 | (15, 18, 19) |

List of electrophysiological scaling coefficients (GX) modulating maximal conductance of ionic current compared to the reference right atrial (RA) cellular framework, with associated reference studies. For each current listed a value of 1 was allocated to its value in RA cells and the ratios shown are relative to this. Key abbreviations: CT (crista terminalis), BB (Bachmann’s bundle), PM (pectinate muscles), AVR (atrioventricular ring), RAA (right atrial appendage), AS (atrial septal region), LA (left atrial chamber), LAA (left atrial appendage), PV (pulmonary venous tissue).


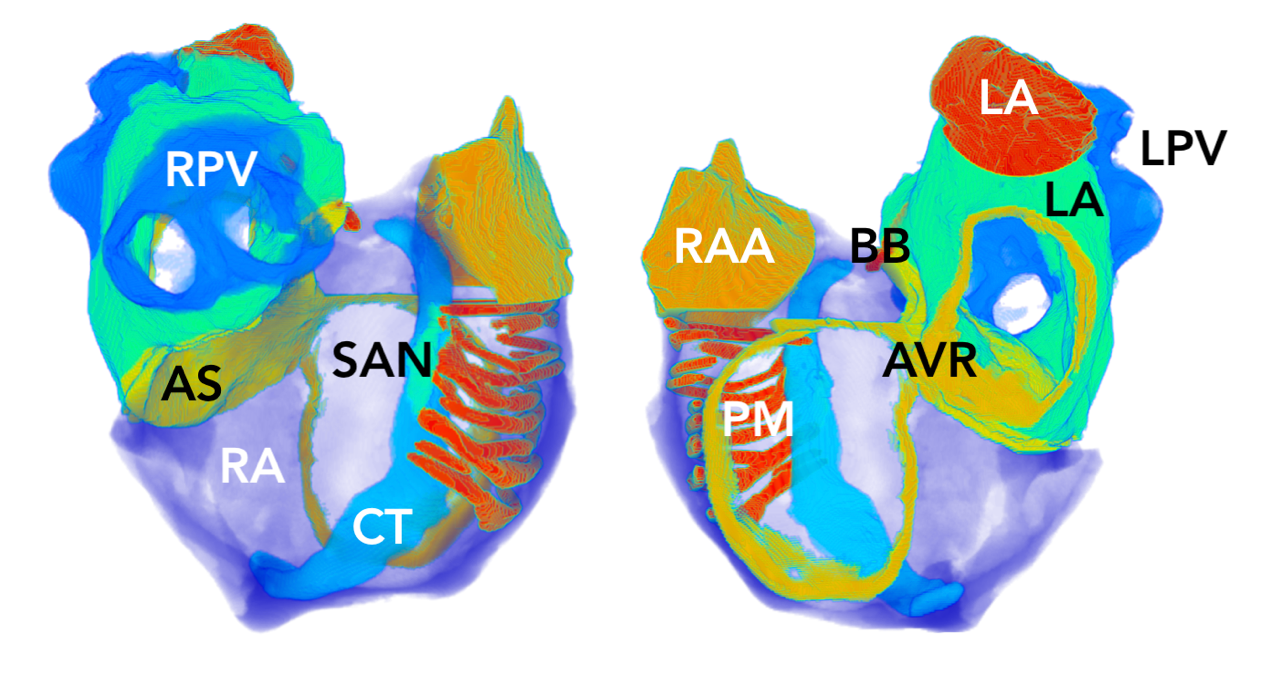


**Fig S4(A). Schematic illustration of variant distinct anatomical regions of the atria, annotated with corresponding anatomical labels.**

The phase distribution method was used in the study to initiate reentry (20), and the initial conditions for all simulations remained the same for purposes of relative comparison of results, as presented in Fig S4(B). In simulations, state variables of 1D atrial tissue from an 1D tissue model were computed, which was then spatially mapped to region of the 3D model following the methodology employed in our previous investigations (4), which facilitated the formation of a three-dimensional (3D) spiral (scroll) wave. Reentrant scroll waves were observed under an appropriate phase map, and WT, mutations, and intermediate deficient $I_{CaL}$ were simulated with the same phase map.

Fig S4(B). **Spatiotemporal initiation of 3D reentry in the human atrial model via phase distribution.** The tissue was initialised by assigning 206 discrete phases, each of which corresponding to a phase state of an action potential of a single right atrial cell (right panel), which was mapped to the 3D spatial region of the left atrium, creating heterogeneous refractoriness. Red regions (positive membrane potential close to 10 mV) denote depolarised/active tissue, while blue regions (resting potential ≤ -80 mV) represent fully repolarised to rest substrate, while the spectrum from red to blue represents the intermediate depolarised region with membrane potential changing from -80 to +10 mV (discretised by 206 phases; see the colour key).


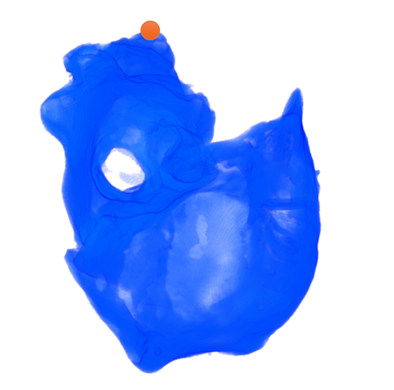


**Fig S4(C). Illustration of the location within the 3D atrial geometry at which the pseudo-ECG was computed.**

For computing the pseudo-ECGs, the electrode to integrate membrane potential was placed at the position as shown in Fig S4(C) (the physical coordinates are (59.4, 59.4, 66.0) mm in the 3D model).

The dispersion of APD was quantitatively assessed in both WT and conditions of deficient $I_{CaL}$, in order to investigate the tissue heterogeneity across 3D atria. The computation of ${APD}_{90}$ used the standard definition as it is at the single-cell level. In simulations of APD, the anatomical 3D virtual atria would be excited with the pacing of the sinoatrial node (SAN) at a frequency of 1 Hz. These results of the difference in normalized $\Delta{APD}_{90}$ for general $I_{CaL}$ deficiency are presented in the study.

# Electrophysiological effects of WT/A39V and corresponding $\boldsymbol{I}_{\boldsymbol{CaL}}$ deficiency

In addition to the study of G490R-related deficient conditions presented in the paper, simulations were conducted under other deficient conditions, including the A39V mutation (on exon 8A) and corresponding f values ranging from 0.2 to 0.8 to represent $I_{\mathrm{CaL}}$ deficiency. The corresponding results are presented and compared with those of the wild type.

- 1. **Characteristic of APs in A39V-based** $\mathbf{I}_{\mathbf{CaL}}$ **deficiency and all mutant types**

# Characteristic of AP in WT and all homozygous mutations

Simulations were conducted at the single-cell level to investigate potential differences in electrophysiology between WT and A39V-related deficient conditions. Table S4 provides the typical characteristics of AP in all types. These results are consistent with those found in G490R-related deficient cases.

**Table S4. Action potential characteristics of deficient I_CaL conditions linked to the A39V mutation.**

| WT/MT | $APA(mV)$ | MUV(V/s) | $RMP(mV)$ | $\mathrm{APD}_{90}(ms)$ |
| --- | --- | --- | --- | --- |
| WT | 99.2 | 192.0 | -75.6 | 247.4 |
| f=0.2 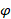 | 99.6 | 194.7 | -75.8 | 235.4 |
| f=0.4 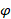 | 99.9 | 196.9 | -76.0 | 224.3 |
| f=0.5 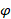 | 100.0 | 198.1 | -76.1 | 212.8 |
| f=0.6 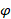 | 100.4 | 200.1 | -76.5 | 139.1 |
| f=0.8 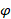 | 100.9 | 204.6 | -77.6 | 65.8 |
| f=1.0 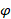 | 101.1 | 206.6 | -78.2 | 45.6 |

Effects of deficient $I_{\mathrm{CaL}}$ conditions linked to the A39V mutation on AP characteristics. The homozygous (f=1), heterozygous (f=0.5) A39V mutation conditions, and intermediate $I_{\mathrm{CaL}}$ deficiency (f=0.2, 0.4, 0.6 and 0.8) resulted in an increase in both the amplitude of the action potential and the maximal upstroke velocity, as compared to the WT. Additionally, $I_{\mathrm{CaL}}$ deficiency caused a more negative resting membrane potential and a shorter duration of the action potential.


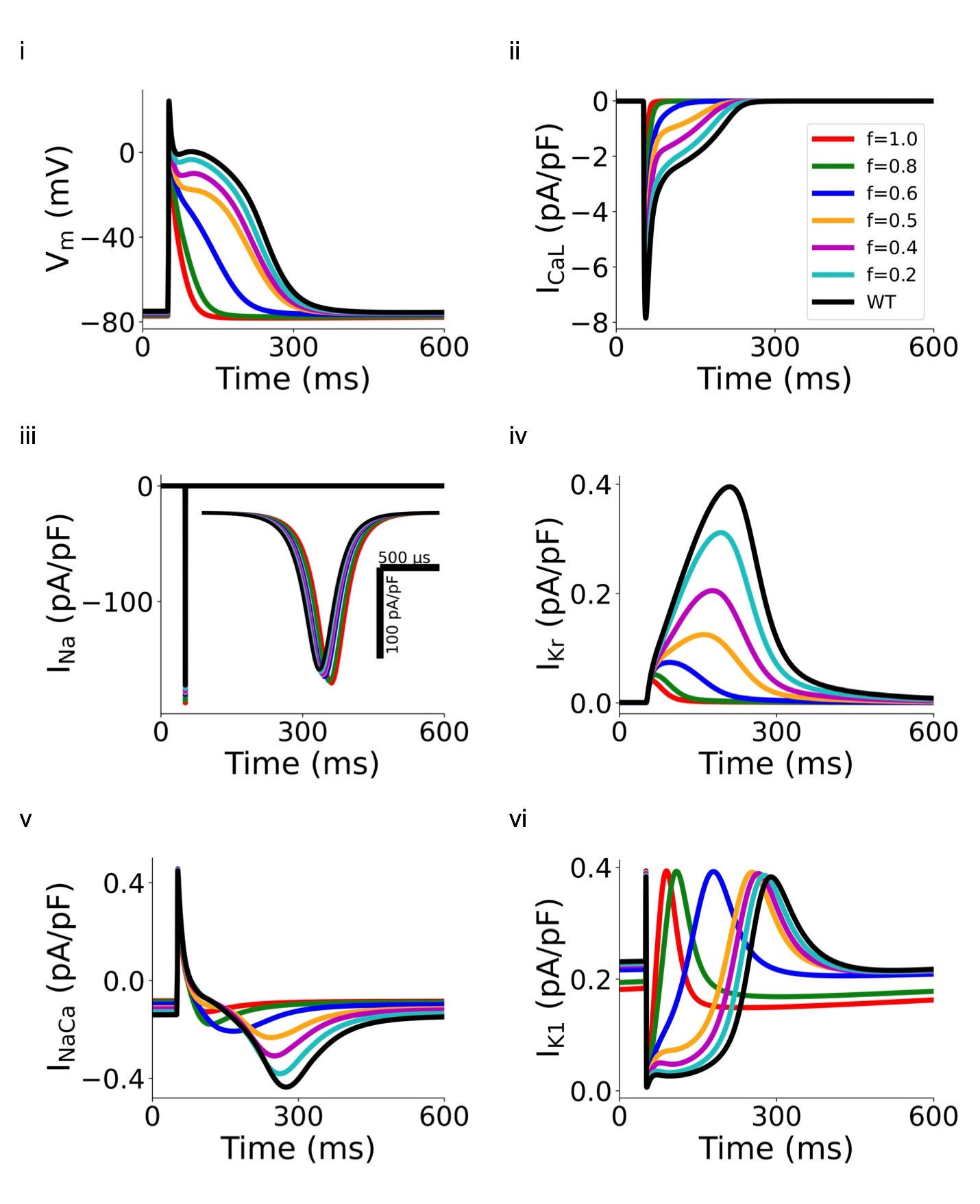


**Fig S5.** **Ionic currents.** Computed APs and related ion channel currents in WT, homozygous(f=1), heterozygous(f=0.5) A39V mutations, and intermediate $I_{\mathrm{CaL}}$ deficiency (f=0.2, 0.4, 0.6 and 0.8) conditions. (i) APs. (ii) $I_{\mathrm{CaL}}$. (iii) $I_{\mathrm{Na}}$. (iv) $I_{\mathrm{Kr}}$. (v) $I_{\mathrm{NaCa}}$. (vi) $I_{K1}$.


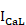

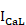

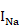

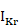

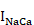

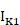

**Fig S6.** **Rate-dependent changes in action potential properties at the single-cell level.​.** Calculated restitution curves of APD in WT, homozygous A39V mutation (f=1) and $I_{\mathrm{CaL}}$ deficiency (f=0.2, 0.4, 0.5, 0.6 and 0.8) conditions. (i) APD restitution (APDr) curve. (ii) The maximal slope of the APDr curves. (iii) ERP restitution (ERPr) curves. (iv) APD and ERP versus various deficient scaling factor f.


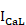


Fig S5 shows the specific action potentials and ion channel currents for WT, $I_{\mathrm{CaL}}$ deficiency, and homozygous/heterozygous A39V mutation conditions. Additionally, rate adaptations of atrial myocytes under deficient $I_{\mathrm{CaL}}$ conditions at the single cell level were evaluated, as shown in Fig S6. The decrease in the maximal slope of APDr illustrates the reduction of rate adaptations in deficient $I_{\mathrm{CaL}}$ conditions, similar to the results for G490R-related deficient cases in the paper.

# Characteristic of AP in WT and all homozygous mutations

Table S5 shows computed characteristics of APs for WT and homozygous A39V, A39V (exon 8), and G490R mutation conditions. All mutations produced a significant decrease in $I_{CaL}$, leading to large reductions in APDs and ERPs. The simulated time courses of APs and several major ion channel currents in WT and homozygous mutation conditions are shown in Fig S7, showing ignorable differences among different types of mutations. A highly deficient $I_{CaL}$ abolished the plateau phase of APs, resulting in accelerated repolarization, and shortened APD and ERP.

**Table S5. Characteristics of APs in WT and all homozygous mutation conditions.**

| WT/MT | $APA(mV)$ | $MUV(V/s)$ | $RMP(mV)$ | $\mathrm{APD}_{90}(ms)$ | $ERP(ms)$ |
| --- | --- | --- | --- | --- | --- |
| $\mathrm{WT}$ | 99.2 | 192.0 | -75.6 | 247.4 | 305 |
| A39V (exon 8) | 101.0 | 205.8 | -78.0 | 51.9 | 90 |
| A39V | 101.1 | 206.6 | -78.2 | 45.6 | 83 |
| $G490R$ | 101.1 | 206.9 | -78.3 | 41.7 | 78 |

# Model independence study

To test the model-dependence of simulation results, another model of human atrial cells developed by Grandi et al. (21) was also used. Results are shown in Fig S8. It was also shown that a great $I_{CaL}$ deficiency by homozygous mutations of A39V, A39V (exon 8) and G490R abbreviated AP, leading to accelerated repolarization and greatly shortened ${APD}_{90}$. Overall electrophysiological effects of homozygous mutations were consistent with that in the CRN model, including shortened APD, reduced $I_{CaL}$, decreased amplitude of $I_{Kr}$ and sodium-calcium exchange current $I_{CaL}$, and early activated $I_{K1}$, suggesting that our observations of AP on great $I_{CaL}$ deficiency due to mutations at the single-cell level were model-independent.


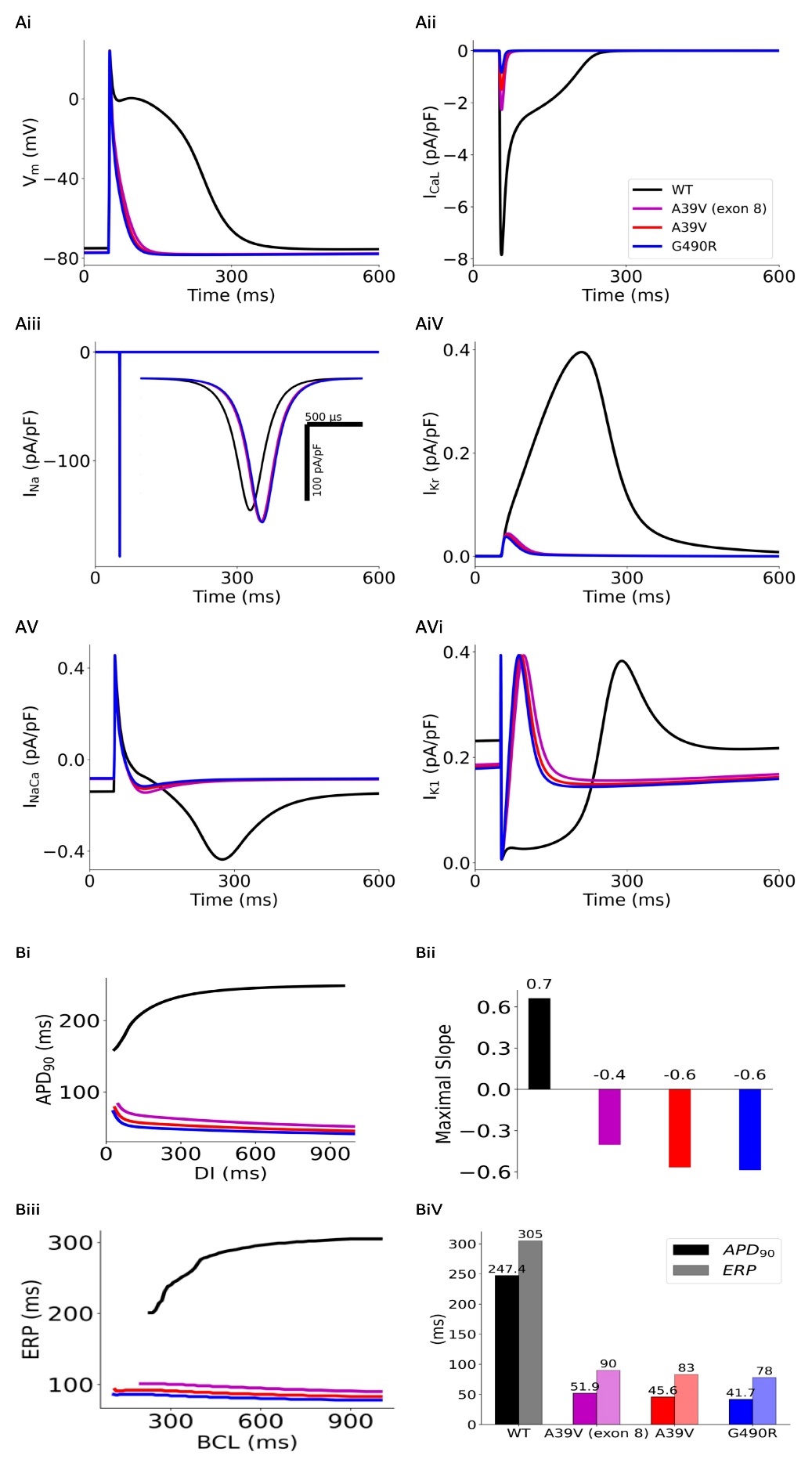


**Fig S7. Characteristics of APs for WT, the homozygous A39V(exon 8), A39V, and G490R mutation conditions at the single-cell level.Bottom of Form**


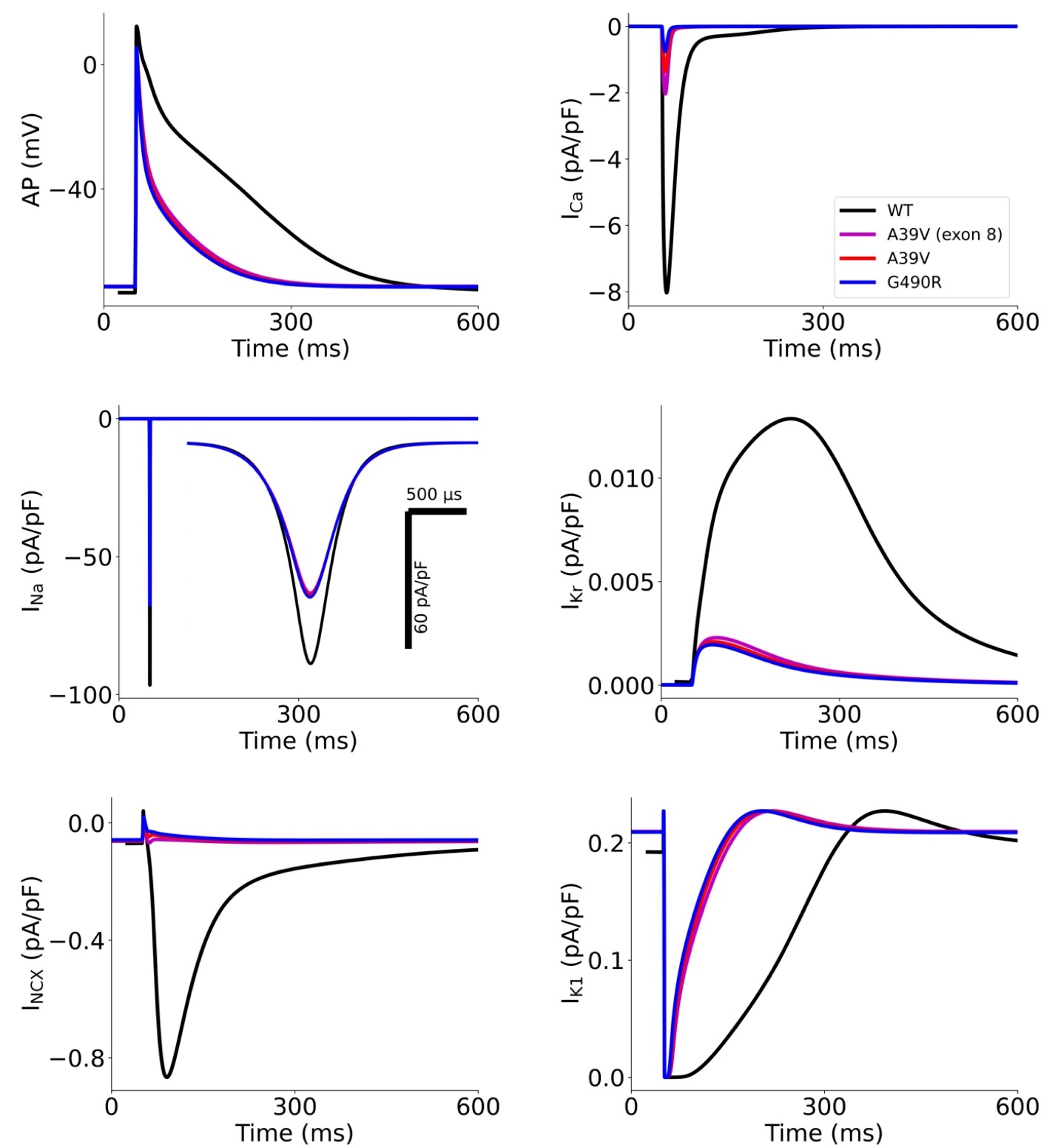


**Fig S8.** **Key ionic currents underlying action potential morphology in WT versus homozygous mutant conditions with the Grandi et al. model.**

- 1. **1D Tissue strand model**

**Fig S9. Temporal vulnerability windows (VW) in atrial tissue under WT and A39V-associated** $\mathbf{I}_{\mathbf{CaL}}$ $I_{\mathrm{CaL}}$ **deficiency.** Panels A and B show the computed VWs at the CT/PM and LA/PV junctions, respectively. Panel C displays a biphasic change in VW width at the CT/PM junction with the scaling factor f. In contrast, at the LA/PV junction (Panel D), the VW width increased monotonically with f, a trend consistent with that observed in G490R-associated cases.

The vulnerability windows in the 1D strand model were assessed for deficient $I_{\mathrm{CaL}}$ conditions linked to the A39V mutation, as shown in Fig S9. The widths of the vulnerability window at the CT/PM junction exhibit biphasic behavior, with the VW width decreasing from the WT to slightly deficient $I_{\mathrm{CaL}}$ conditions (f≤0.5), and expanding under severely deficient $I_{\mathrm{CaL}}$ conditions (f≥0.8). In the context of the LA/PV junction, the width of the vulnerability window increased in all deficient $I_{\mathrm{CaL}}$ conditions, in contrast to the wild-type configuration.

- 1. **Dynamics of reentries in two-dimensional idealized sheet**

Fig S10 presents snapshots of the spatial distribution of transmembrane potential (S10i, S10ii, S10iii) in a two-dimensional sheet, time series of integrated membrane potential across the entire 2D sheet (S10v), and the power spectrum of the computed time series (S10vi). The area of meander of the core of the spiral waves was also calculated, as shown in Fig S10iv, which illustrates the biphasic behavior. The area of tip movement increased from the WT to slight $I_{\mathrm{CaL}}$ deficiency (f$\leq0.4$) and decreased within greatly deficient tissue (f$\geq0.5$). The biphasic effect of the meandering area is consistent with those in deficient investigations with G490R mutation-based $I_{\mathrm{CaL}}$ deficiency. The maintenance of re-entries for greatly deficient $I_{\mathrm{CaL}}$ conditions (f≥0.5) suggests the pro-arrhythmic effects of severely deficient scenarios, as shown in Fig S10vi. The lifespan of re-entries, dominant frequency, and core trace meandering area are given in Table S6.

**Table S6: Two-dimensional simulation results for A39V corresponding deficient** $\mathbf{I}_{\mathbf{CaL}}$ $I_{\mathrm{CaL}}$ **conditions.**

|  | WT | f=0.2 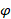 | f=0.4 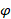 | f=0.5 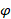 | f=0.6 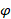 | f=0.8 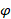 | f=1 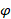 |
| --- | --- | --- | --- | --- | --- | --- | --- |
| LS (ms) | 5400 | 6900 | 6000 | 8000 | 8000 | 8000 | 8000 |
| $DF (Hz)$ | 3.8 | 3.9 | 4.3 | 4.6 | 5.1 | 6.1 | 6.8 |
| Tip Meander Area ($\mathrm{cm}^{2}$ $\mathrm{cm}^{2}$) | 14.9 | 18.5 | 43.4 | 42.4 | 11.6 | 7.2 | 5.0 |

Lifespans of reentries and computed dominant frequency (DF) of time series, and spiral wave core movement area of re-entrant excitations in WT, CACNA1C A39V mutation and corresponding deficient $I_{\mathrm{CaL}}$ $I_{\mathrm{CaL}}$ conditions in the 2D model.


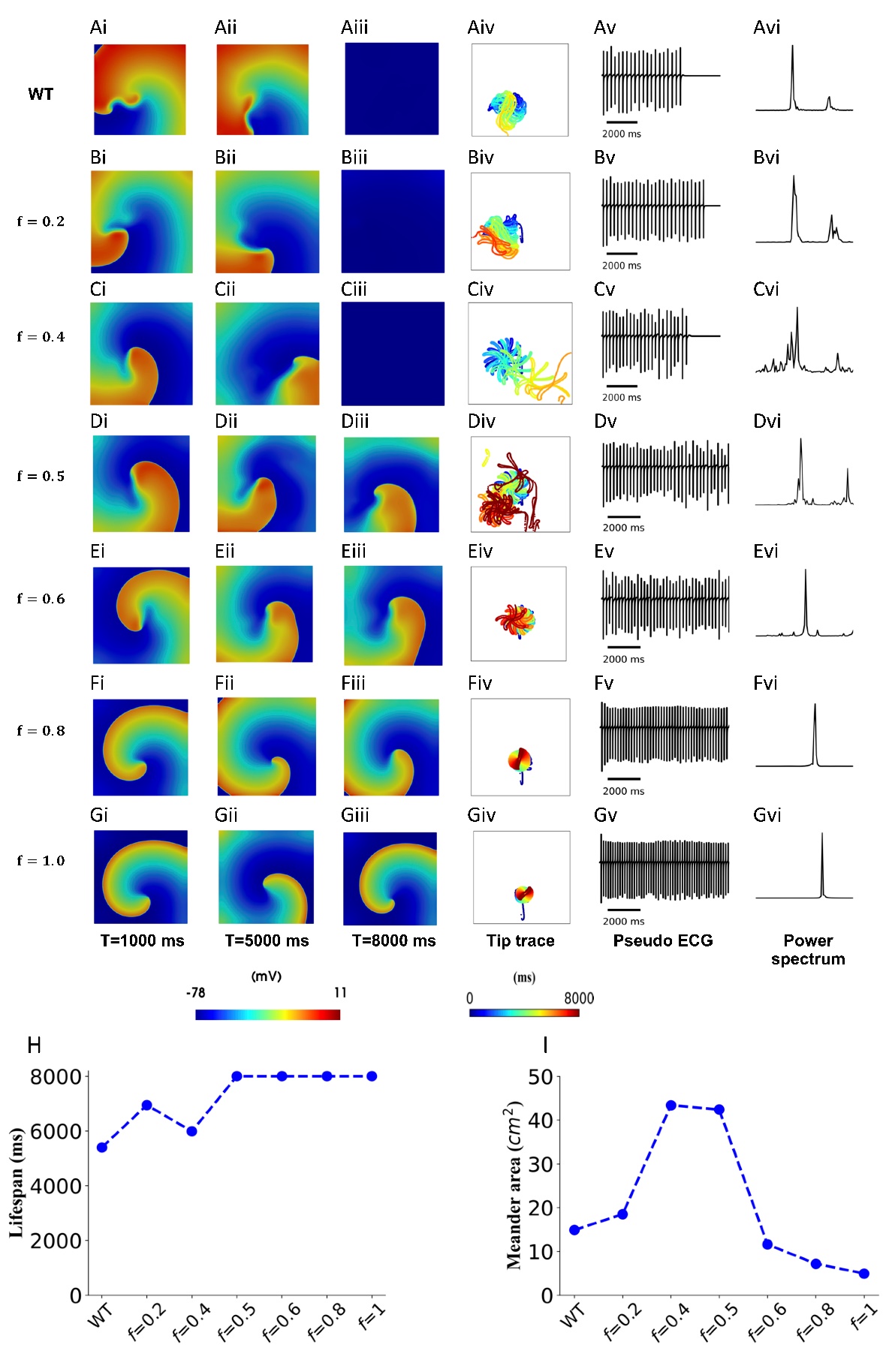


**Fig S10. Dynamics of re-entrant spiral waves under WT, intermediate**$I_{\mathrm{CaL}}$**deficiency, and homozygous A39V mutation conditions.​​** For each condition, WT (A), intermediate $I_{\mathrm{CaL}}$ deficiency (B-F) and homozygous *CACNA1C* A39V mutation (G) conditions. Snapshots of reentry (e.g. Ai to Aiii), tip trace pattern (e.g. Aiv), time series of integrated transmembrane potential across the entire 2D tissue (e.g Av), and its power spectrum (e.g Avi) are shown. The lifespan of reentries is documented in Fig S10(H). Fig S10(I) presents the meandering area of core trajectory for the wild-type and A39V-associated $I_{\mathrm{CaL}}$ deficient conditions.


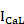


- 1. **Dynamics of scroll waves in 3D human atrial anatomical model**

**Fig S11.** **Properties of simulated scroll waves in 3D models.** (A) Bar charts of $\mathrm{APD}_{90}$ across the whole 3D human atrial tissue in the WT, A39V mutation, and corresponding deficient $I_{\mathrm{CaL}}$ conditions. (B) The lifespan of re-entrant scroll waves in WT, A39V mutation, and intermediate deficient $I_{\mathrm{CaL}}$ conditions. (C) Normalized APD dispersion: $\Delta APD_{90}$versus deficient scaling factor f. (D) The lifespan of 3D re-entrant scroll versus deficient coefficient f.


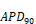

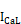

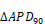

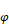


Fig S11 and Fig S12 present three-dimensional simulations of the human atria, representing the WT, $I_{\mathrm{CaL}}$ deficiency and A39V mutation conditions. Fig S11(A) displays the range of $\mathrm{APD}_{90}$ across the entire 3D atrial tissue, while Fig S11(B) illustrates the lifespan of reentrant scroll waves under all conditions. Fig S11(C) shows the normalized dispersion of $\mathrm{APD}_{90}$ across different tissue types, revealing a biphasic trend. Fig S11(D) delineates lifespans through dashed lines, displaying biphasic characteristics. The biphasic patterns in normalized $\Delta{APD}_{90}$ and lifespan trends recur, similar to those observed in the context of $I_{\mathrm{CaL}}$ deficiency linked to the G490R mutation.


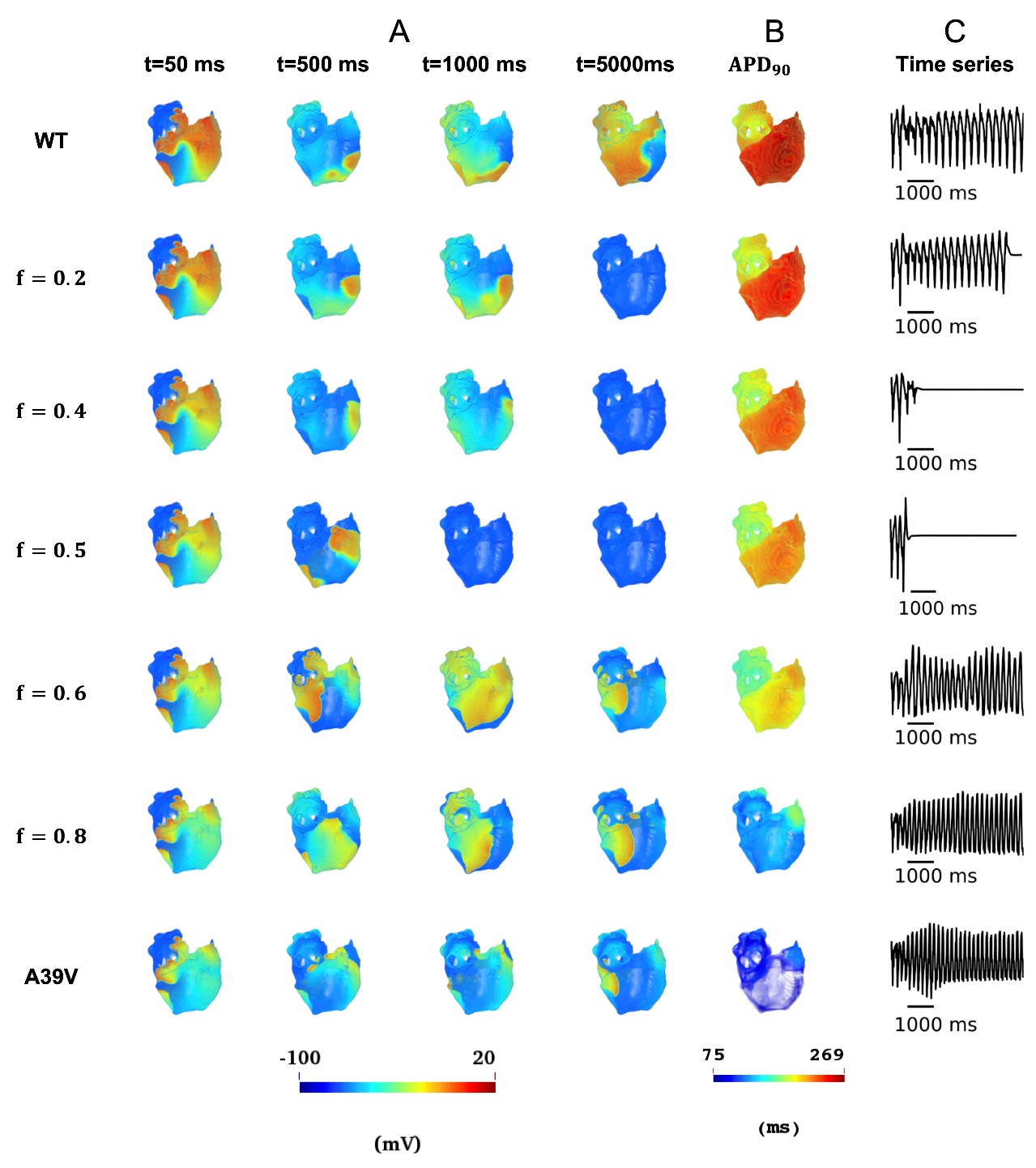


**Fig S12.** **Three-dimensional simulations.** (A) Snapshots of scroll waves at different timings (labelled on top of the figure) from 3D model simulations for WT, homozygous CACNA1C A39V mutation, and its corresponding intermediate deficient conditions. The action potential was coloured coded from -100 mV in blue to 20 mV in dark red, with the colour key shown at the bottom. (B) Colour mapping of APD dispersion in 3D human atria. APD dispersion was colour coded from 75 ms in blue to 269 ms in dark red. Colour key is shown at the bottom. (C) Time series of integrated action potential over the whole 3D human atrial space.

Fig S12(A) displays reentrant patterns in three-dimensional virtual human atria under different conditions, including WT, $I_{\mathrm{CaL}}$ deficiency, and the homozygous A39V mutation. Fig S12(B) displays the spatial distribution of $\mathrm{APD}_{90}$ across the entire 3D virtual human atrial construct. Additionally, Fig S12(C) presents the calculated time series representing the integrated membrane potential spanning the entirety of the 3D atrial tissue. Table S7 provides information on the lifespan and dominant frequency of reentrant excitation waves in the three-dimensional atrial model.

**Table S7: Three-dimensional simulation results for A39V corresponding deficient** $\mathbf{I}_{\mathbf{CaL}}$ $I_{\mathrm{CaL}}$ **conditions.**

| WT/MT  metrics | WT | f=0.2 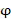 | f=0.4 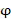 | f=0.5 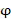 | f=0.6 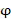 | f=0.8 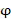 | f=1 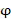 (A39V) |
| --- | --- | --- | --- | --- | --- | --- | --- |
| $Lifespan (ms)$ | 5000 | 4500 | 1100 | 800 | 5000 | 5000 | 5000 |
| $DF (HZ)$ | 3.7 | 3.8 | 3.9 | / | 4.6 | 5.5 | 6.0 |

Lifespan and computed dominant frequency (DF) of scroll waves in WT, CACNA1C A39V mutation, and corresponding deficient $I_{\mathrm{CaL}}$ conditions within the 3D model.


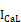


# Electrophysiological effects of deficient $\mathbf{I}_{\mathbf{CaL}}$ into all mutations

In the section, we evaluated the pro-arrhythmogenic effects of the homozygous G490R, A39V and A39V (exon 8) mutations with parameters listed in S2 Table.

- 1. **1D tissue strand model**

Fig S13 shows the results of restitution curves of CV, ERP, and WL as well as the excitation threshold computed from a 1D atrial strand model. It was shown that the restitution curves of CV, ERP and WL in all mutations presented similar features, but were significantly different from those in the wild type. At fast rates, all mutation tissue enhanced tissue excitability, facilitating excitation propagation.


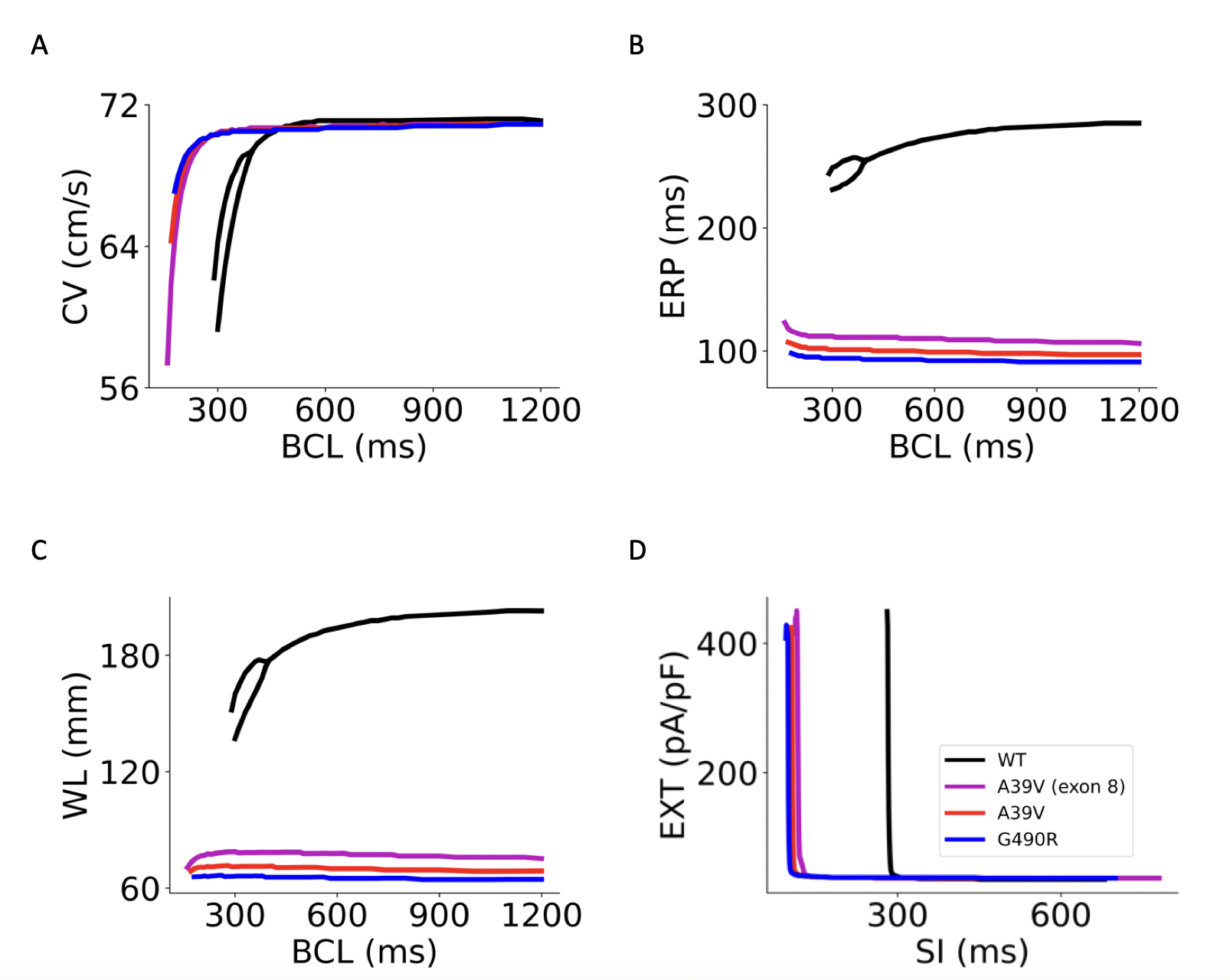


**Fig S13.** **One-dimensional simulations for WT and all homozygous mutations.** Restitution curves of conduction velocity (A), effective refractory period (B), and wavelength (C) of excitation waves for all homozygous mutations versus varying BCLs. Tissue refractoriness is determined via restitution curves of excitation threshold versus S1-S2 interval, as shown in panel (D).

Fig S14 shows the measured VW in a 1D model of the heterogeneous atrial strand in WT and mutations. At both the CT/PM junction and the LA/PV junction, homozygous mutations increased the width of VW, showing their pro-arrhythmogenic effects.


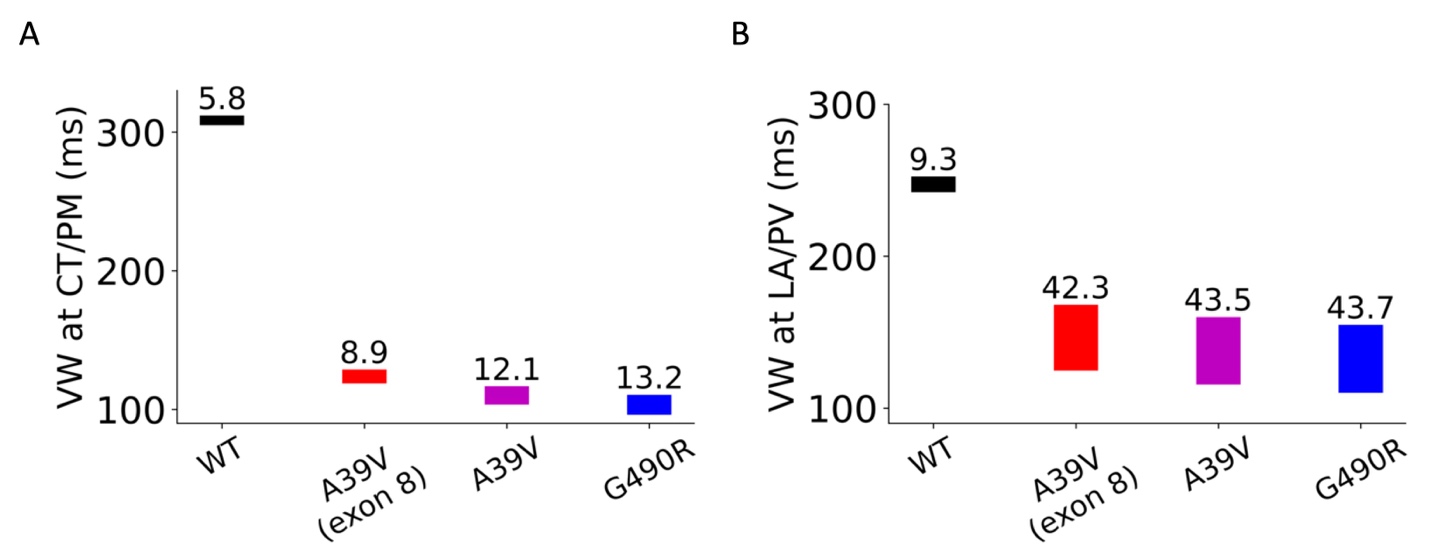


**Fig S14.** **Temporal vulnerability windows for WT and all homozygous mutations.** Measured temporal vulnerability windows at the CT/PM junction (A) and LA/PV junction (B). All homozygous mutations led to increased VW widths at both CT/PM and LA/PV junctions.

- 1. **Dynamics of reentries in two-dimensional sheet model**

Fig S15 shows simulated spiral waves in a 2D model of atrial mutant tissue. Snapshots of reentrant excitation waves at different timings (Ai-Aiii), tip trajectory of meandering (Aiv), time course of integrated transmembrane potentials across the 2D tissue (Av) and power spectrum (Avi) are shown for WT. Corresponding results for mutations are shown in Bi-Bvi, Ci-Cvi and Di-Dvi respectively. The homozygous mutations increased the stationarity of meandering spiral waves, and increased the dominant frequency of atrial excitations.

**Fig S15.** **Two-dimensional simulations for WT and all homozygous mutations.** Snapshots of spiral waves in the 2D substrate model, panel i, ii and iii representing spiral waves at time equal to 1000, 5000 and 8000 ms respectively. Panel iv represented the trajectories of the core of re-entrant excitations. Panel v represents the time series of integrated transmembrane potential across the entire 2D sheet. Panel vi represents the corresponding power spectrum of the time series.

**6.3 Dynamics of scroll waves in the 3D human atrial anatomical model**

Using an appropriate phase map as initial conditions, scroll waves in the atria in WT, homozygous A39V (exon 8), A39V, and G490R mutations were initiated. As depicted in Fig S16, all homozygous CACNA1C A39V, A39V (exon 8) and G490R mutations led to sustained reentries. The time series of re-entrant excitations by integrating membrane potential across 3D virtual atria for WT and all mutation conditions are presented in the last column of Fig S16, panel V. The mutations accelerated atrial reentrant excitation waves.


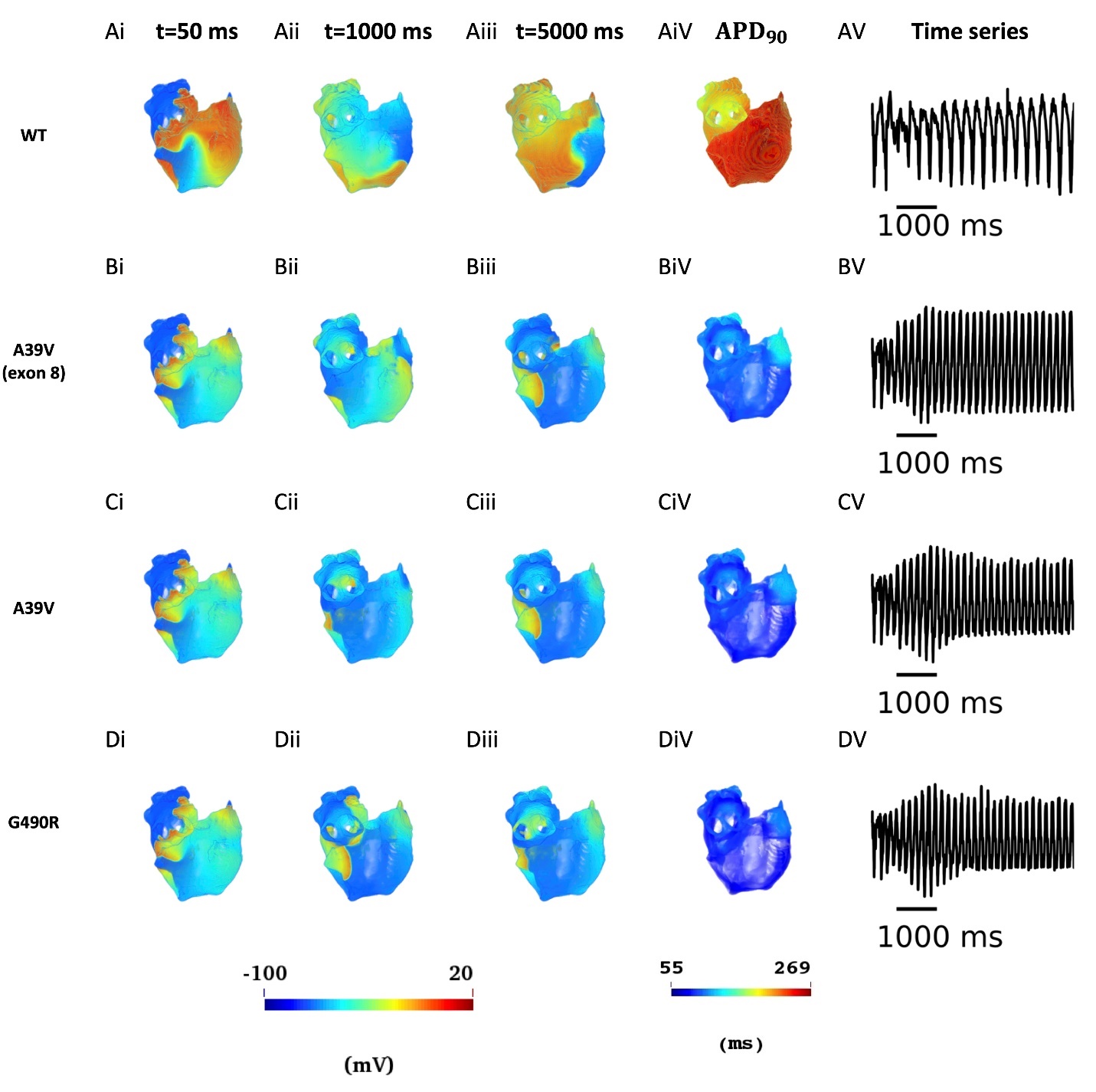


**Fig S16.** **Dynamics of re-entrant excitation in a 3D virtual human atrium.** Snapshots of re-entrant excitation waves at three different time points (panel i-iii). The spatial distribution of $\mathrm{APD}_{90}$ is displayed in panel iV. Time series of integrated membrane potential across 3D tissue are given in panel V.

**6.4 APD dispersion at the 3D tissue level**

Fig S17 shows the ${APD}_{90}$ range across the 3D human atrial anatomical tissue for WT and mutation conditions. In simulations, ${APD}_{90}$ was measured in the whole 3D anatomical tissue model, from which the maximal APD spatial dispersion ($\Delta{APD}_{90})$ was computed, and its range was exhibited in Fig S17A. As APD dispersion ($\Delta{APD}_{90})$ was significantly affected by magnitudes of ${APD}_{90}$, in our studies, the ($\Delta{APD}_{90}$) was normalized by the mid-range ${APD}_{90}$ ($({APD}_{90}^{min} +{APD}_{90}^{max})/2$) across whole 3D atrial tissue. It was shown that the mutations increased APD dispersion, as shown in Fig S17B. The normalized $\Delta{APD}_{90}$ in mutations were greater than that in WT, which implied an enlarged APD dispersion by a deficient I_CaL_.

**Fig S17.** **Action potential duration and its dispersion in 3D human atria under WT and mutant conditions.​​** (A). Global ${APD}_{90}$ in 3D virtual human atria for WT and all mutation conditions. (B). Normalized $\Delta{APD}_{90}$ for WT, A39V (exon 8), A39V, G490R mutations. APD dispersion ${\Delta APD}_{90}$ was normalized by midrange of APD across 3D atria in each case. The normalized APD dispersion was increased in all mutation conditions, compared to that in WT.

**Reference**

1. Courtemanche M, Ramirez RJ, Nattel S. Ionic mechanisms underlying human atrial action potential properties: insights from a mathematical model. Am J Physiol. 1998;275(1):H301-21.

2. Antzelevitch C, Pollevick GD, Cordeiro JM, Casis O, Sanguinetti MC, Aizawa Y, et al. Loss-of-function mutations in the cardiac calcium channel underlie a new clinical entity characterized by ST-segment elevation, short QT intervals, and sudden cardiac death. Circulation. 2007;115(4):442-9.

3. Nelder JA, Mead R. A Simplex Method for Function Minimization. The Computer Journal. 1965;7(4):308-13.

4. Whittaker DG, Ni H, El Harchi A, Hancox JC, Zhang H. Atrial arrhythmogenicity of KCNJ2 mutations in short QT syndrome: Insights from virtual human atria. PLoS Comput Biol. 2017;13(6):e1005593.

5. Keren G, Etzion T, Sherez J, Zelcer AA, Megidish R, Miller HI, et al. Atrial fibrillation and atrial enlargement in patients with mitral stenosis. Am Heart J. 1987;114(5):1146-55.

6. Colman MA, Aslanidi OV, Kharche S, Boyett MR, Garratt C, Hancox JC, et al. Pro-arrhythmogenic effects of atrial fibrillation-induced electrical remodelling: insights from the three-dimensional virtual human atria. J Physiol. 2013;591(17):4249-72.

7. Bray MA, Wikswo JP. Use of topological charge to determine filament location and dynamics in a numerical model of scroll wave activity. IEEE Trans Biomed Eng. 2002;49(10):1086-93.

8. Mitra S, Mitra M, Chaudhuri BB. Generation of digital time database from paper ECG records and Fourier transform-based analysis for disease identification. Comput Biol Med. 2004;34(7):551-60.

9. Aslanidi OV, Colman MA, Stott J, Dobrzynski H, Boyett MR, Holden AV, et al. 3D virtual human atria: A computational platform for studying clinical atrial fibrillation. Prog Biophys Mol Biol. 2011;107(1):156-68.

10. Kharche S, Adeniran I, Stott J, Law P, Boyett MR, Hancox JC, et al. Pro-arrhythmogenic effects of the S140G KCNQ1 mutation in human atrial fibrillation - insights from modelling. J Physiol. 2012;590(18):4501-14.

11. Rush S, Larsen H. A practical algorithm for solving dynamic membrane equations. IEEE Trans Biomed Eng. 1978;25(4):389-92.

12. Feng J, Yue L, Wang Z, Nattel S. Ionic mechanisms of regional action potential heterogeneity in the canine right atrium. Circulation research. 1998;83(5):541-51.

13. Burashnikov A, Mannava S, Antzelevitch C. Transmembrane action potential heterogeneity in the canine isolated arterially perfused right atrium: effect of I Kr and I Kur/I to block. American Journal of Physiology-Heart and Circulatory Physiology. 2004;286(6):H2393-H400.

14. Gong D, Zhang Y, Cai B, Meng Q, Jiang S, Li X, et al. Characterization and comparison of Na+, K+ and Ca2+ currents between myocytes from human atrial right appendage and atrial septum. Cellular Physiology and Biochemistry. 2008;21(5-6):385-94.

15. Ehrlich JR, Cha TJ, Zhang L, Chartier D, Melnyk P, Hohnloser SH, et al. Cellular electrophysiology of canine pulmonary vein cardiomyocytes: action potential and ionic current properties. The Journal of physiology. 2003;551(3):801-13.

16. Li D, Zhang L, Kneller J, Nattel S. Potential ionic mechanism for repolarization differences between canine right and left atrium. Circulation research. 2001;88(11):1168-75.

17. Caballero R, de la Fuente MG, Gómez R, Barana A, Amorós I, Dolz-Gaitón P, et al. In humans, chronic atrial fibrillation decreases the transient outward current and ultrarapid component of the delayed rectifier current differentially on each atria and increases the slow component of the delayed rectifier current in both. Journal of the American College of Cardiology. 2010;55(21):2346-54.

18. Cha T-J, Ehrlich JR, Zhang L, Chartier D, Leung TK, Nattel S. Atrial tachycardia remodeling of pulmonary vein cardiomyocytes: comparison with left atrium and potential relation to arrhythmogenesis. Circulation. 2005;111(6):728-35.

19. Datino T, Macle L, Qi X-Y, Maguy A, Comtois P, Chartier D, et al. Mechanisms by which adenosine restores conduction in dormant canine pulmonary veins. Circulation. 2010;121(8):963-72.

20. Biktashev VN, Holden AV. Reentrant waves and their elimination in a model of mammalian ventricular tissue. Chaos. 1998;8(1):48-56.

21. Grandi E, Pandit SV, Voigt N, Workman AJ, Dobrev D, Jalife J, et al. Human atrial action potential and Ca2+ model: sinus rhythm and chronic atrial fibrillation. Circ Res. 2011;109(9):1055-66.
