# Supplementary figures and images for: Biphasic effects on human atrial arrhythmogenicity of L-type calcium channel mutations associated with a Brugada/Short QT overlap syndrome - insights from a multiscale simulation study
Source: PLoS Comput Biol. 2025 Nov 19;21(11):e1013616. doi: 10.1371/journal.pcbi.1013616 (PMC12629484; doi:10.1371/journal.pcbi.1013616)

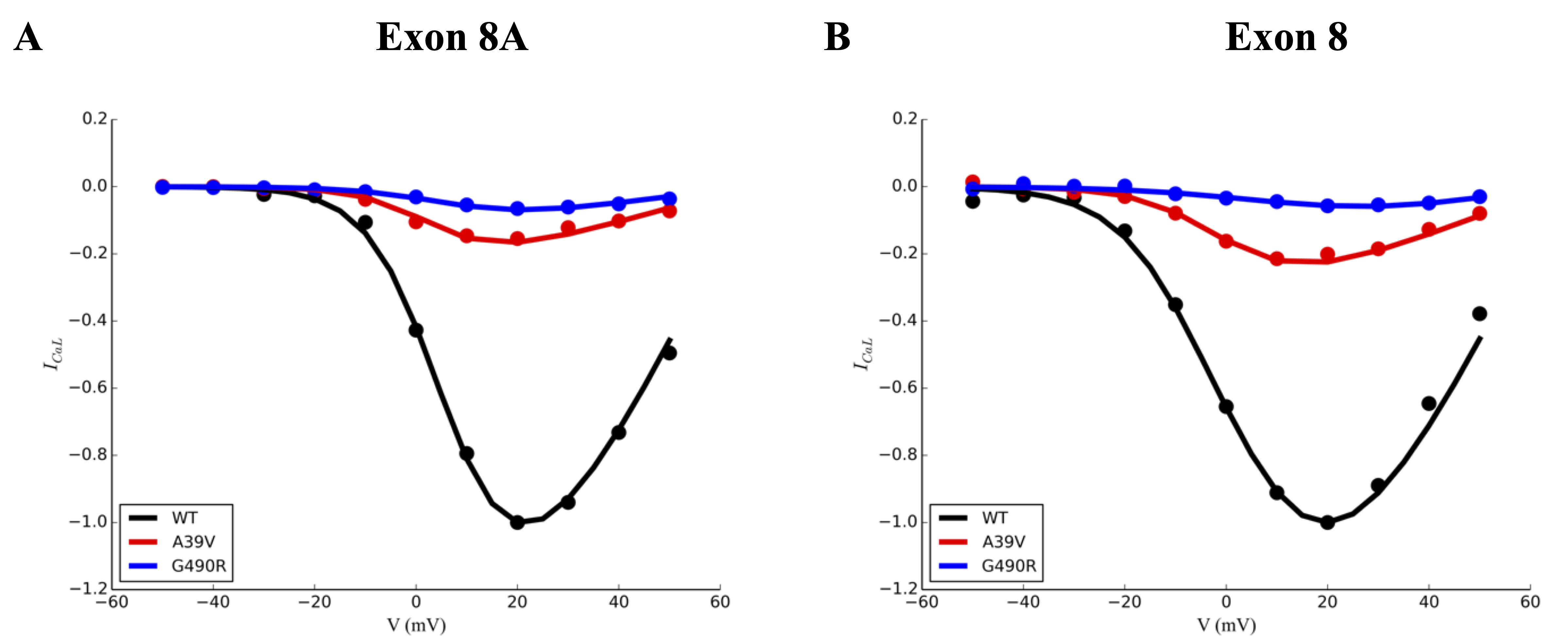

Supplement: S1 Fig — Experimental data (dots) from patch-clamp recordings are compared with simulations (line) generated by the modified CRN model. Model parameters are provided in S1 Table. (TIFF) [file pcbi.1013616.s002.tiff]

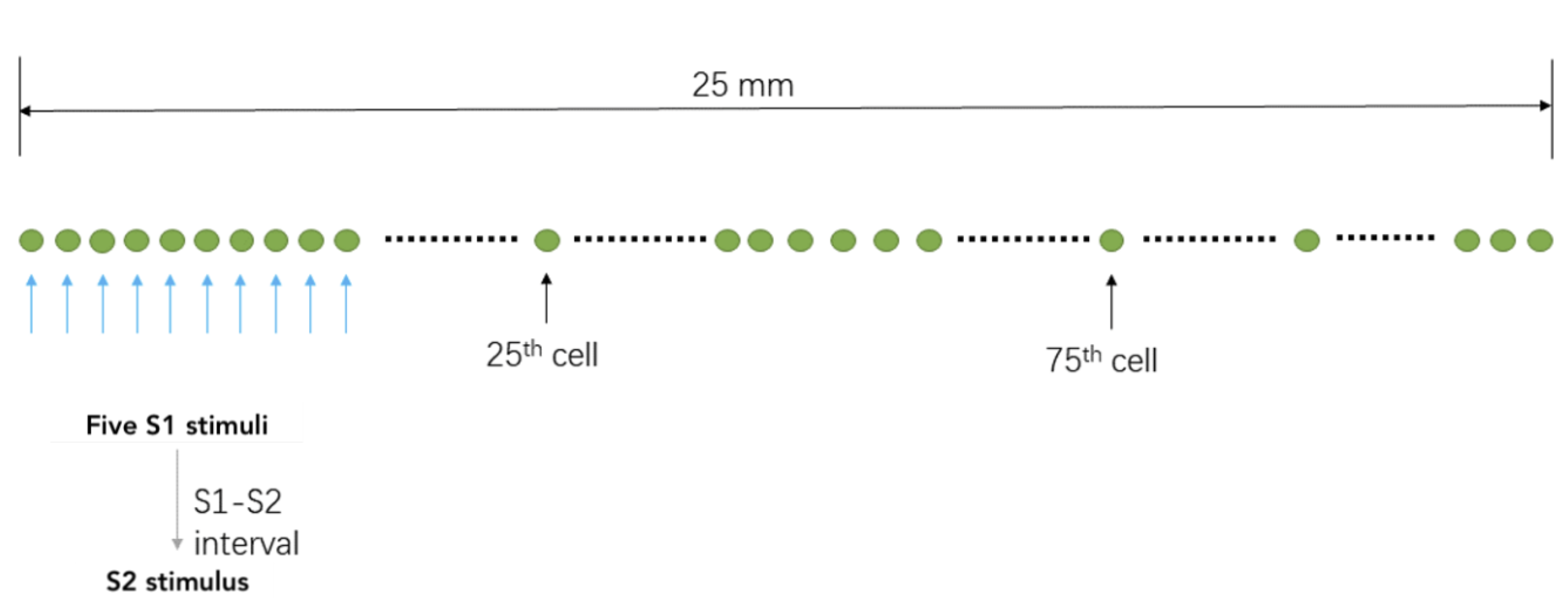

Supplement: S2 Fig — The model represents a strand of tissue composed of 100 isotropic nodes (spatial resolution: 0.25 mm). Conduction velocity was determined by recording the activation time difference between the 25th and 75th nodes. The effective refractory period (ERP) and excitability threshold (EXT) were assessed using an S1-S2 pacing protocol. (TIFF) [file pcbi.1013616.s003.tiff]

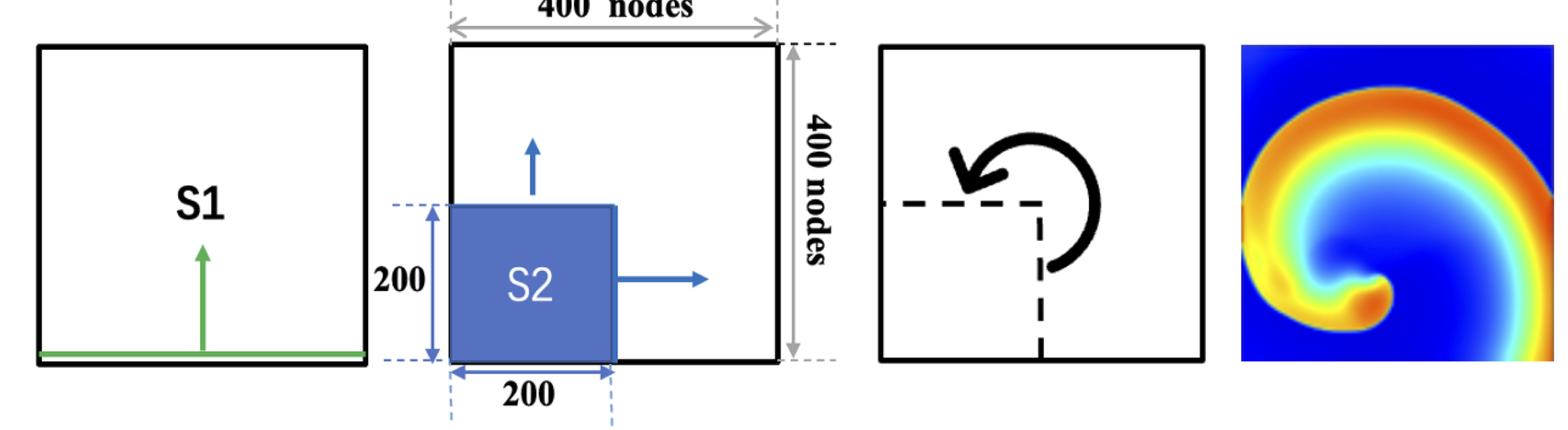

Supplement: S3 Fig — S1 stimuli were applied at the lower edge of the 2D sheet, while the S2 stimulus was applied at the lower-left region of the sheet, occupying one-fourth of the total area of the 2D sheet, which consists of 200x200 nodes. The interaction between the S1 and S2-evoked excitation waves led to the formation of reentrant excitation waves. (TIFF) [file pcbi.1013616.s004.tiff]

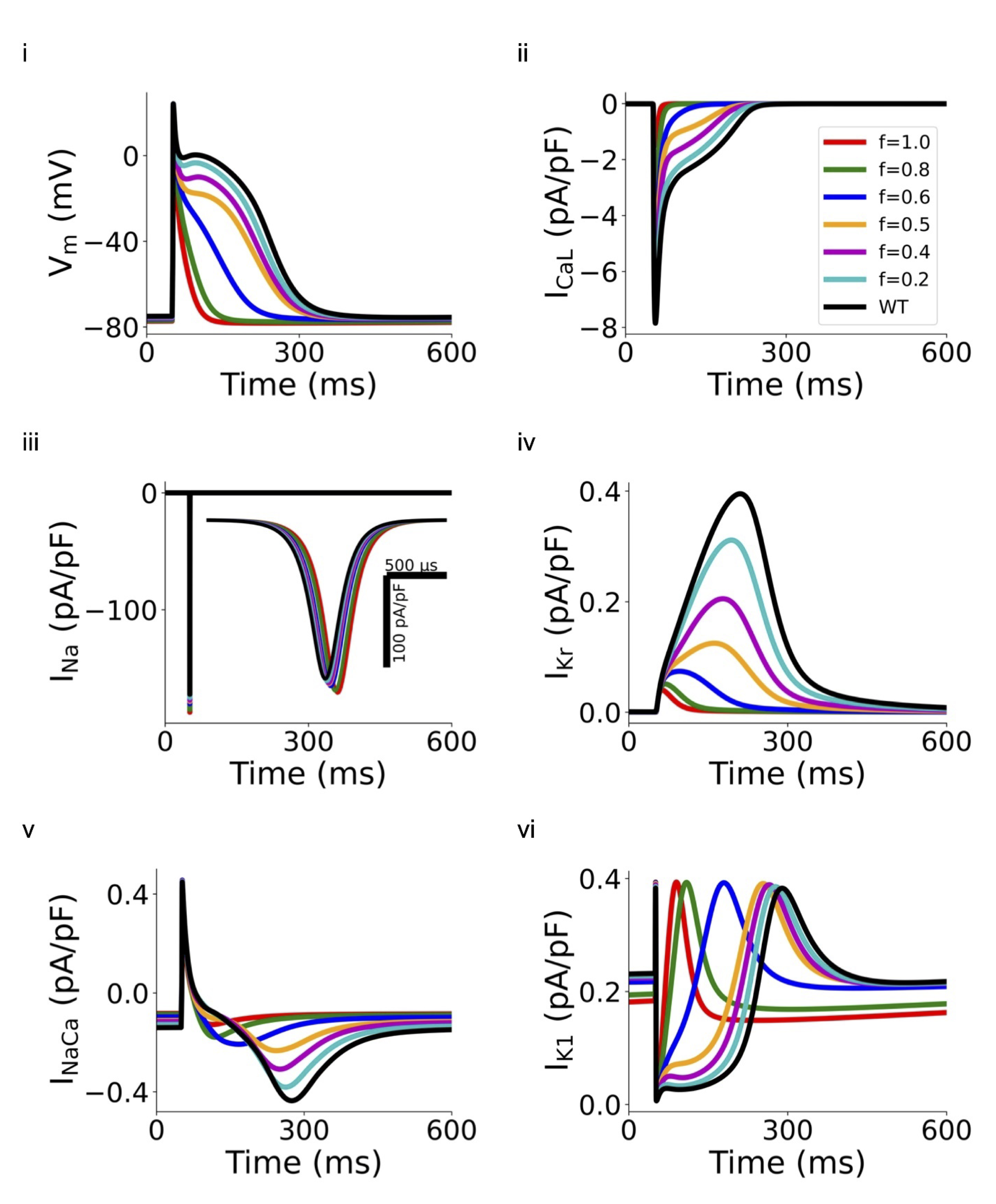

Supplement: S5 Fig — Computed APs and related ion channel currents in WT, homozygous(f = 1), heterozygous(f = 0.5) A39V mutations, and intermediate ICaL deficiency (f = 0.2, 0.4, 0.6 and 0.8) conditions. (i) APs. (ii) ICaL. (iii) INa. (iv) IKr. (v) INaCa. (vi) IK1. (TIFF) [file pcbi.1013616.s006.tiff]

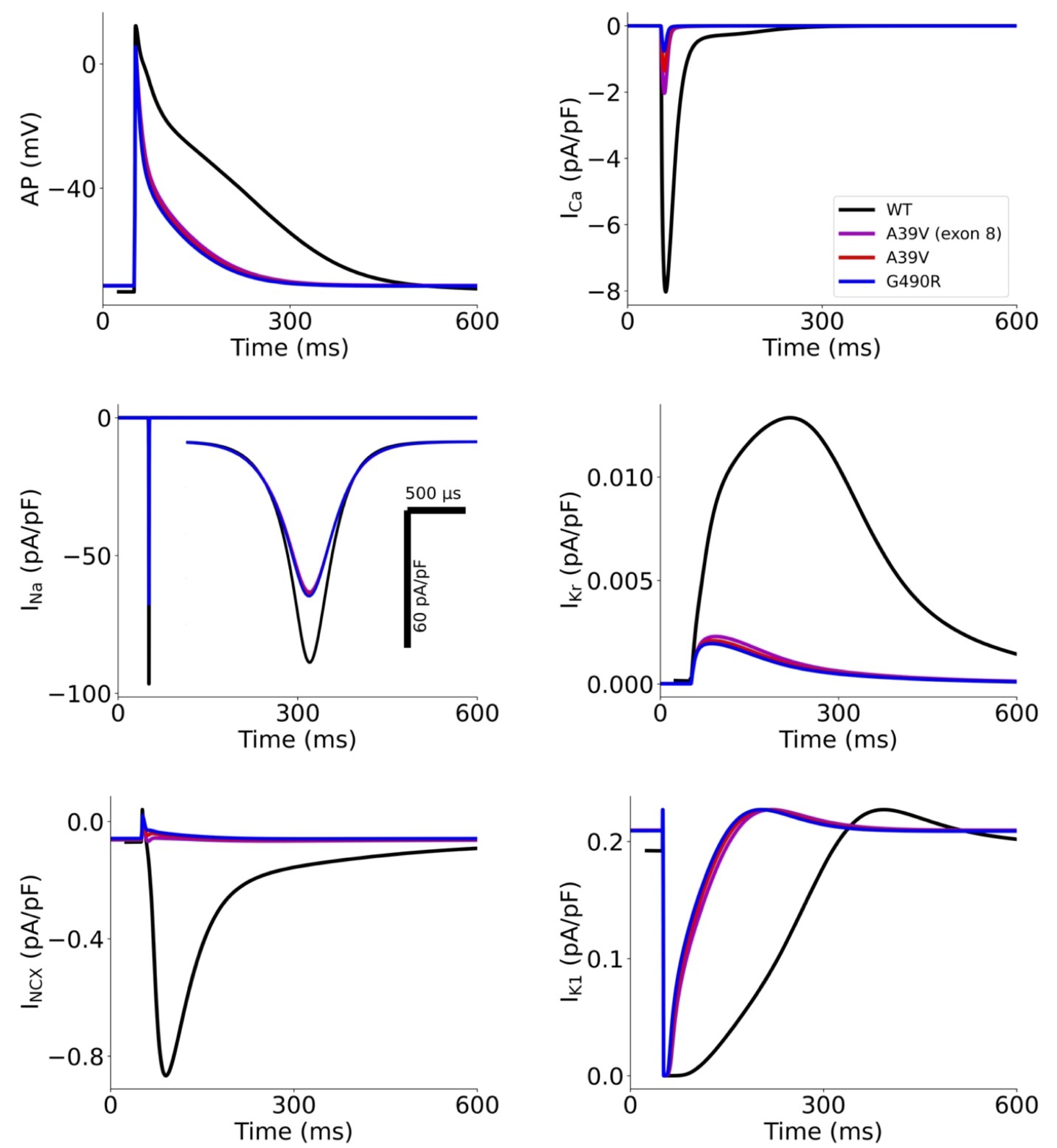

Supplement: S8 Fig — (TIFF) [file pcbi.1013616.s009.tiff]

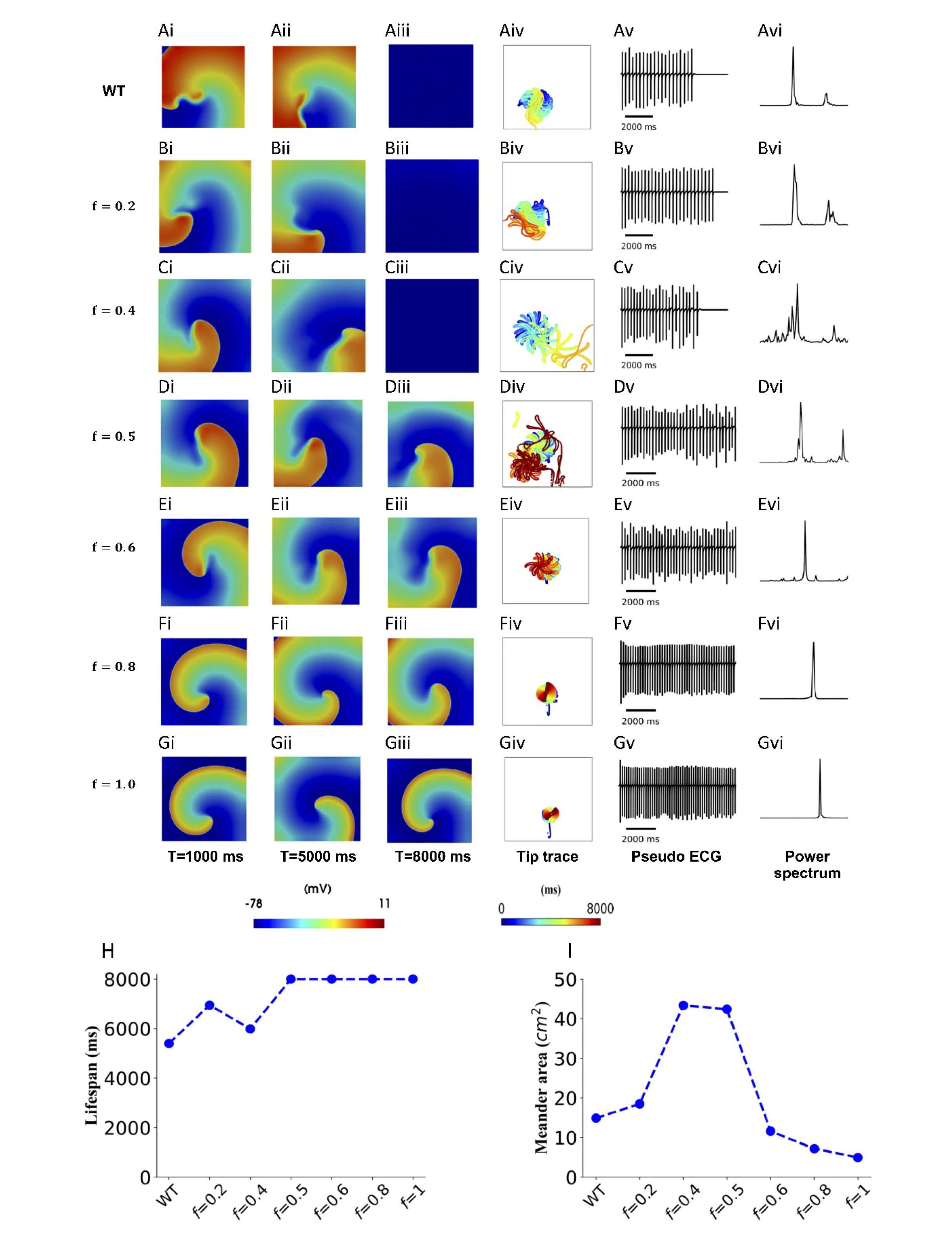

Supplement: S10 Fig — For each condition, WT (A), intermediate ICaL deficiency (B-F) and homozygous CACNA1C A39V mutation (G) conditions. Snapshots of reentry (e.g., Ai to Aiii), tip trace pattern (e.g., Aiv), time series of integrated transmembrane potential across the entire 2D tissue (e.g., Av), and its power spectrum (e.g., Avi) are shown. The lifespan of reentries is documented in Fig S10(H). Fig S10(I) presents the meandering area of core trajectory for the wild-type and A39V-associated ICaL deficient conditions. (TIFF) [file pcbi.1013616.s011.tiff]

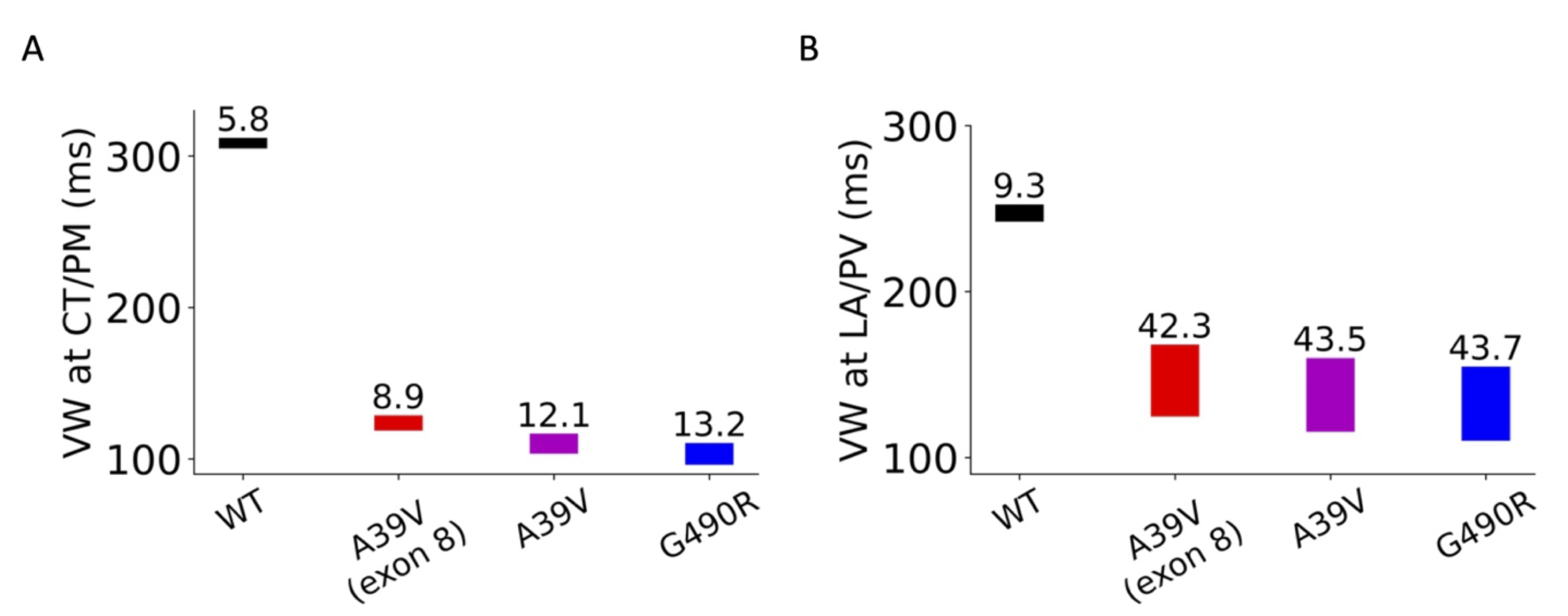

Supplement: S14 Fig — Measured temporal vulnerability windows at the CT/PM junction (A) and LA/PV junction (B). All homozygous mutations led to increased VW widths at both CT/PM and LA/PV junctions. (TIFF) [file pcbi.1013616.s015.tiff]

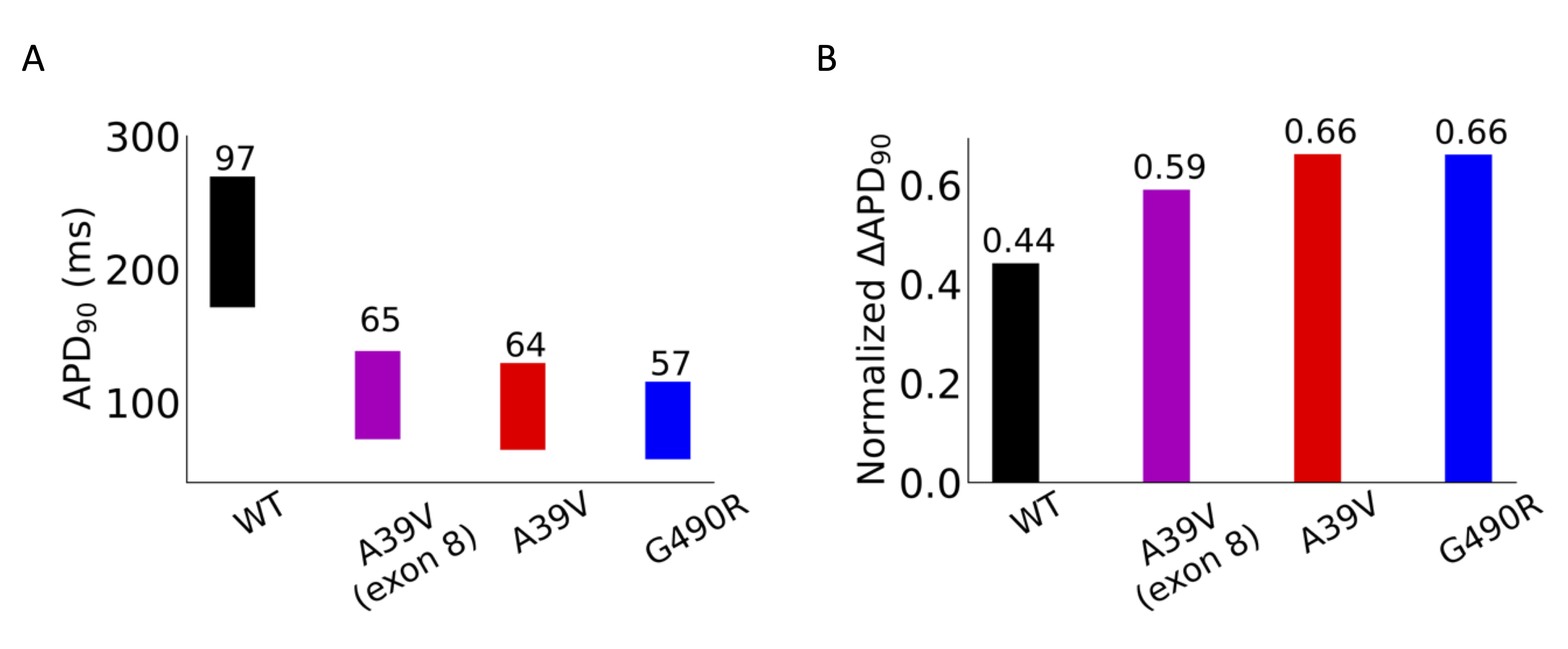

Supplement: S17 Fig — (A). Global APD90 in 3D virtual human atria for WT and all mutation conditions. (B). Normalized ΔAPD90 for WT, A39V (exon 8), A39V, G490R mutations. APD dispersion ΔAPD90 was normalized by midrange of APD across 3D atria in each case. The normalized APD dispersion was increased in all mutation conditions, compared to that in WT. (TIFF) [file pcbi.1013616.s018.tiff]
