# Supplementary material for: Biphasic effects on human atrial arrhythmogenicity of L-type calcium channel mutations associated with a Brugada/Short QT overlap syndrome - insights from a multiscale simulation study
Source: PLoS Comput Biol. 2025 Nov 19;21(11):e1013616. doi: 10.1371/journal.pcbi.1013616 (PMC12629484; doi:10.1371/journal.pcbi.1013616)
Supplement: S4 Fig — (B). Spatiotemporal initiation of 3D reentry in the human atrial model via phase distribution. The tissue was initialised by assigning 206 discrete phases, each of which corresponding to a phase state of an action potential of a single right atrial cell (right panel), which was mapped to the 3D spatial region of the left atrium, creating heterogeneous refractoriness. Red regions (positive membrane potential close to 10 mV) denote depolarised/active tissue, while blue regions (resting potential ≤ -80 mV) represent fully repolarised to rest substrate, while the spectrum from red to blue represents the intermediate depolarised region with membrane potential changing from -80 to +10 mV (discretised by 206 phases; see the colour. (C). Illustration of the location within the 3D atrial geometry at which the pseudo-ECG was computed.key). (DOCX) [file pcbi.1013616.s005.docx]

**Fig S4**

**Biphasic effects of on human atrial arrhythmogenicity of L-type calcium channel mutations associated with a Brugada/Short QT overlap syndrome - insights from a multiscale simulation study**

Yirong Xiang, Jules C. Hancox, Henggui Zhang


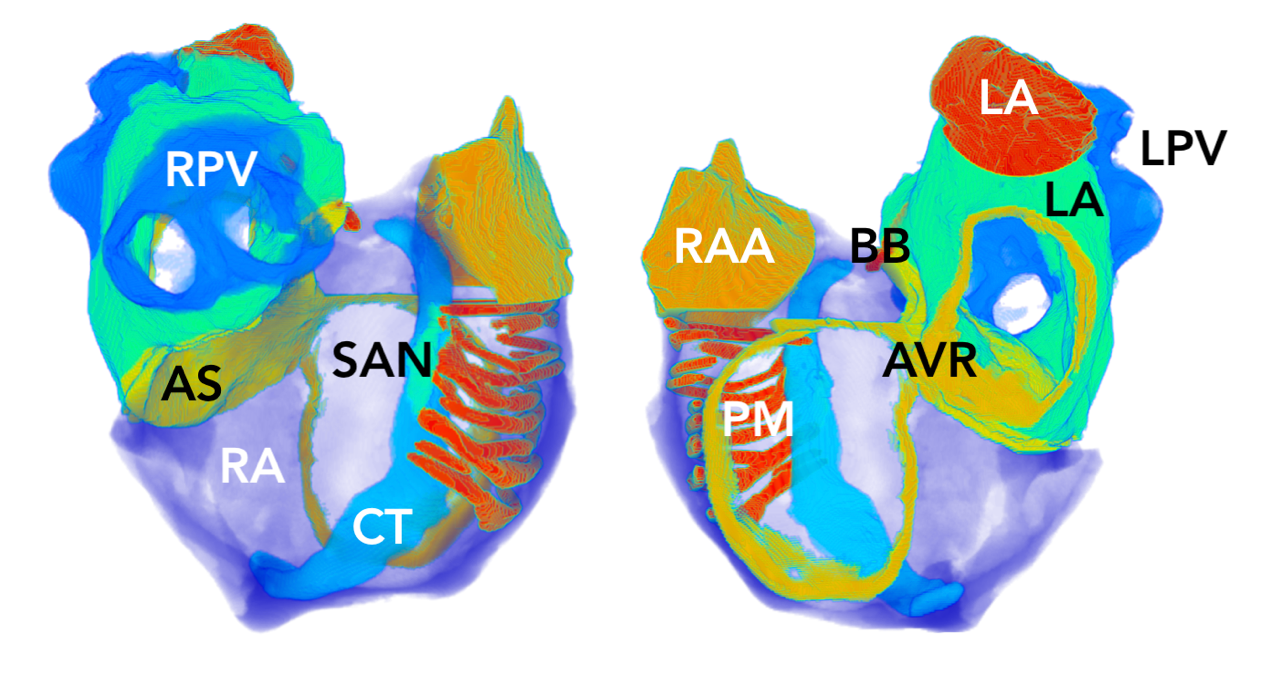


**Figure S4(A). Schematic illustration of variant distinct anatomical regions of the atria, annotated with corresponding anatomical labels.**

**Figure S4(B). Spatiotemporal initiation of 3D reentry in the human atrial model via phase distribution.** The tissue was initialised by assigning 206 discrete phases, each of which corresponding to a phase state of an action potential of a single right atrial cell (right panel), which was mapped to the 3D spatial region of the left atrium, creating heterogeneous refractoriness. Red regions (positive membrane potential close to 10 mV) denote depolarised/active tissue, while blue regions (resting potential ≤ -80 mV) represent fully repolarised to rest substrate, while the spectrum from red to blue represents the intermediate depolarised region with membrane potential changing from -80 to +10 mV (discretised by 206 phases; see the colour key).


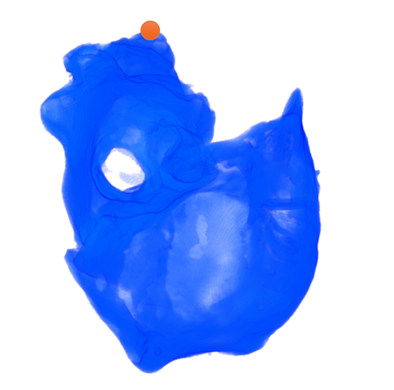


**Figure S4(C). Illustration of the location within the 3D atrial geometry at which the pseudo-ECG was computed.**
