# Supplementary material for: Biphasic effects on human atrial arrhythmogenicity of L-type calcium channel mutations associated with a Brugada/Short QT overlap syndrome - insights from a multiscale simulation study
Source: PLoS Comput Biol. 2025 Nov 19;21(11):e1013616. doi: 10.1371/journal.pcbi.1013616 (PMC12629484; doi:10.1371/journal.pcbi.1013616)
Supplement: S1 Table — Parameters of modified CRN model to match the simulated I-V curves to the experimental data presented by Antzelevitch et al. (DOCX) [file pcbi.1013616.s019.docx]

**Table S1**

**Biphasic effects of on human atrial arrhythmogenicity of L-type calcium channel mutations associated with a Brugada/Short QT overlap syndrome - insights from a multiscale simulation study**

Yirong Xiang, Jules C. Hancox, Henggui Zhang

**Table S1. I_CaL_ formulation parameters.**

| **Model component** | | **Exon 8A** | | | **Exon 8** | | |
| --- | --- | --- | --- | --- | --- | --- | --- |
|  |  | **WT** | **A39V** | **G490R** | **WT** | **A39V** | **G490R** |
| Activation | $V_{d\frac{1}{2}} (mV)$ | -18.0 | - 14.3 | -18.0 | -12.6 | -11.0 | -25.9 |
|  | ${grad}_{d}$ | 0.8 | 0.8 | $\text{1}\text{.}\text{0}$ | 1.0 | 0.9 | 1.5 |
| Inactivation | $V_{h\frac{1}{2}} (mV)$ | 40.1 | 40.1 | 49.8 | 50.7 | 50.7 | 50.7 |
|  | ${grad}_{h}$ | 1.5 | 1.5 | 1.5 | 0.6 | 1.1 | 0.7 |
| Scaling factor of the maximum channel conductance (g_CaL_) relative to that in the basal model | | × 1.39 | $\times$ 0.18 | $\times$ 0.10 | $\times$ 1.73 | $\times$ 0.35 | $\times$ 0.12 |

Parameters of modified CRN model to match the simulated I-V curves to the experimental data presented by Antzelevitch et al. (1).

1. Antzelevitch C, Pollevick GD, Cordeiro JM, Casis O, Sanguinetti MC, Aizawa Y, et al. Loss-of-function mutations in the cardiac calcium channel underlie a new clinical entity characterized by ST-segment elevation, short QT intervals, and sudden cardiac death. Circulation. 2007;115(4):442-9.
