# Supplementary material for: Biphasic effects on human atrial arrhythmogenicity of L-type calcium channel mutations associated with a Brugada/Short QT overlap syndrome - insights from a multiscale simulation study
Source: PLoS Comput Biol. 2025 Nov 19;21(11):e1013616. doi: 10.1371/journal.pcbi.1013616 (PMC12629484; doi:10.1371/journal.pcbi.1013616)
Supplement: S2 Table — (DOCX) [file pcbi.1013616.s020.docx]

**Table S2**

**Biphasic effects of on human atrial arrhythmogenicity of L-type calcium channel mutations associated with a Brugada/Short QT overlap syndrome - insights from a multiscale simulation study**

Yirong Xiang, Jules C. Hancox, Henggui Zhang

**Table S2. Scaling factor of gCaL for WT and homozygous mutation cases.**

| WT/MT | WT | G490R | A39V | A39V (exon 8) |
| --- | --- | --- | --- | --- |
| Relative to $g_{CaL}$ | 1 | 0.07 | 0.13 | 0.20 |
