# Supplementary material for: Biphasic effects on human atrial arrhythmogenicity of L-type calcium channel mutations associated with a Brugada/Short QT overlap syndrome - insights from a multiscale simulation study
Source: PLoS Comput Biol. 2025 Nov 19;21(11):e1013616. doi: 10.1371/journal.pcbi.1013616 (PMC12629484; doi:10.1371/journal.pcbi.1013616)
Supplement: S3 Table — List of electrophysiological scaling coefficients (GX) modulating maximal conductance of ionic current compared to the reference right atrial (RA) cellular framework, with associated reference studies. For each current listed a value of 1 was allocated to its value in RA cells and the ratios shown are relative to this. Key abbreviations: CT (crista terminalis), BB (Bachmann’s bundle), PM (pectinate muscles), AVR (atrioventricular ring), RAA (right atrial appendage), AS (atrial septal region), LA (left atrial chamber), LAA (left atrial appendage), PV (pulmonary venous tissue). (DOCX) [file pcbi.1013616.s021.docx]

**Table S3**

**Biphasic effects of on human atrial arrhythmogenicity of L-type calcium channel mutations associated with a Brugada/Short QT overlap syndrome - insights from a multiscale simulation study**

Yirong Xiang, Jules C. Hancox, Henggui Zhang

**Table S3. Regional scaling coefficients of maximal ionic conductance.**

|  | $G_{CaL}$ | $G_{to}$ | $G_{Kur}$ | $G_{Na}$ | $G_{Kr}$ | $G_{Ks}$ | $G_{K1}$ | Paper Source |
| --- | --- | --- | --- | --- | --- | --- | --- | --- |
| CT | 1.68 | 1.35 | 1.0 | 1.0 | 1.0 | 1.0 | 1.0 | (1, 2) |
| BB | 1.72 | 1.35 | 1.0 | 1.0 | 1.0 | 1.0 | 1.0 | (1, 2, 3) |
| PM | 0.94 | 1.0 | 1.0 | 1.0 | 1.0 | 1.0 | 1.0 | (1, 2) |
| AVR | 0.67 | 0.6 | 1.0 | 1.0 | 1.63 | 1.0 | 1.0 | (2) |
| RAA | 1.0 | 0.68 | 1.0 | 1.0 | 1.0 | 1.0 | 1.0 | (2, 4) |
| AS | 0.4 | 0.212 | 0.667 | 1.3 | 1.0 | 1.0 | 1.0 | (4) |
| LA | 1.0 | 1.0 | 1.0 | 1.0 | 1.6 | 1.0 | 1.0 | (2, 5, 6) |
| LAA | 1.0 | 0.53 | 0.8 | 1.0 | 1.6 | 1.0 | 1.0 | (2, 6, 7) |
| PV | 0.7 | 0.75 | 1.0 | 1.0 | 2.4 | 1.5 | 0.62 | (5, 8, 9) |

List of electrophysiological scaling coefficients (GX) modulating maximal conductance of ionic current compared to the reference right atrial (RA) cellular framework, with associated reference studies. For each current listed a value of 1 was allocated to its value in RA cells and the ratios shown are relative to this. Key abbreviations: CT (crista terminalis), BB (Bachmann’s bundle), PM (pectinate muscles), AVR (atrioventricular ring), RAA (right atrial appendage), AS (atrial septal region), LA (left atrial chamber), LAA (left atrial appendage), PV (pulmonary venous tissue).

1. Colman MA, Aslanidi OV, Kharche S, Boyett MR, Garratt C, Hancox JC, et al. Pro-arrhythmogenic effects of atrial fibrillation-induced electrical remodelling: insights from the three-dimensional virtual human atria. J Physiol. 2013;591(17):4249-72.

2. Feng J, Yue L, Wang Z, Nattel S. Ionic mechanisms of regional action potential heterogeneity in the canine right atrium. Circulation research. 1998;83(5):541-51.

3. Burashnikov A, Mannava S, Antzelevitch C. Transmembrane action potential heterogeneity in the canine isolated arterially perfused right atrium: effect of I Kr and I Kur/I to block. American Journal of Physiology-Heart and Circulatory Physiology. 2004;286(6):H2393-H400.

4. Gong D, Zhang Y, Cai B, Meng Q, Jiang S, Li X, et al. Characterization and comparison of Na+, K+ and Ca2+ currents between myocytes from human atrial right appendage and atrial septum. Cellular Physiology and Biochemistry. 2008;21(5-6):385-94.

5. Ehrlich JR, Cha TJ, Zhang L, Chartier D, Melnyk P, Hohnloser SH, et al. Cellular electrophysiology of canine pulmonary vein cardiomyocytes: action potential and ionic current properties. The Journal of physiology. 2003;551(3):801-13.

6. Li D, Zhang L, Kneller J, Nattel S. Potential ionic mechanism for repolarization differences between canine right and left atrium. Circulation research. 2001;88(11):1168-75.

7. Caballero R, de la Fuente MG, Gómez R, Barana A, Amorós I, Dolz-Gaitón P, et al. In humans, chronic atrial fibrillation decreases the transient outward current and ultrarapid component of the delayed rectifier current differentially on each atria and increases the slow component of the delayed rectifier current in both. Journal of the American College of Cardiology. 2010;55(21):2346-54.

8. Cha T-J, Ehrlich JR, Zhang L, Chartier D, Leung TK, Nattel S. Atrial tachycardia remodeling of pulmonary vein cardiomyocytes: comparison with left atrium and potential relation to arrhythmogenesis. Circulation. 2005;111(6):728-35.

9. Datino T, Macle L, Qi X-Y, Maguy A, Comtois P, Chartier D, et al. Mechanisms by which adenosine restores conduction in dormant canine pulmonary veins. Circulation. 2010;121(8):963-72.
