# Supplementary material for: Biphasic effects on human atrial arrhythmogenicity of L-type calcium channel mutations associated with a Brugada/Short QT overlap syndrome - insights from a multiscale simulation study
Source: PLoS Comput Biol. 2025 Nov 19;21(11):e1013616. doi: 10.1371/journal.pcbi.1013616 (PMC12629484; doi:10.1371/journal.pcbi.1013616)
Supplement: S4 Table — Effects of deficient ICaL conditions linked to the A39V mutation on AP characteristics. The homozygous (f = 1), heterozygous (f = 0.5) A39V mutation conditions, and intermediate ICaL deficiency (f = 0.2, 0.4, 0.6 and 0.8) resulted in an increase in both the amplitude of the action potential and the maximal upstroke velocity, as compared to the WT. Additionally, ICaL deficiency caused a more negative resting membrane potential and a shorter duration of the action potential. (DOCX) [file pcbi.1013616.s022.docx]

**Table S4**

**Biphasic effects of on human atrial arrhythmogenicity of L-type calcium channel mutations associated with a Brugada/Short QT overlap syndrome - insights from a multiscale simulation study**

Yirong Xiang, Jules C. Hancox, Henggui Zhang

**Table S4. Action potential characteristics of deficient I_CaL conditions linked to the A39V mutation.**

| WT/MT | $APA(mV)$ | MUV(V/s) | $RMP(mV)$ | $\mathrm{APD}_{90}(ms)$ |
| --- | --- | --- | --- | --- |
| WT | 99.2 | 192.0 | -75.6 | 247.4 |
| f=0.2 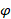 | 99.6 | 194.7 | -75.8 | 235.4 |
| f=0.4 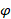 | 99.9 | 196.9 | -76.0 | 224.3 |
| f=0.5 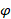 | 100.0 | 198.1 | -76.1 | 212.8 |
| f=0.6 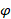 | 100.4 | 200.1 | -76.5 | 139.1 |
| f=0.8 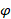 | 100.9 | 204.6 | -77.6 | 65.8 |
| f=1.0 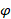 | 101.1 | 206.6 | -78.2 | 45.6 |

Effects of deficient $I_{\mathrm{CaL}}$ conditions linked to the A39V mutation on AP characteristics. The homozygous (f=1), heterozygous (f=0.5) A39V mutation conditions, and intermediate $I_{\mathrm{CaL}}$ deficiency (f=0.2, 0.4, 0.6 and 0.8) resulted in an increase in both the amplitude of the action potential and the maximal upstroke velocity, as compared to the WT. Additionally, $I_{\mathrm{CaL}}$ deficiency caused a more negative resting membrane potential and a shorter duration of the action potential.
