# Supplementary material for: Biphasic effects on human atrial arrhythmogenicity of L-type calcium channel mutations associated with a Brugada/Short QT overlap syndrome - insights from a multiscale simulation study
Source: PLoS Comput Biol. 2025 Nov 19;21(11):e1013616. doi: 10.1371/journal.pcbi.1013616 (PMC12629484; doi:10.1371/journal.pcbi.1013616)
Supplement: S5 Table — (DOCX) [file pcbi.1013616.s023.docx]

**Table S5**

**Biphasic effects of on human atrial arrhythmogenicity of L-type calcium channel mutations associated with a Brugada/Short QT overlap syndrome - insights from a multiscale simulation study**

Yirong Xiang, Jules C. Hancox, Henggui Zhang

**Table S5. Characteristics of APs in WT and all homozygous mutation conditions.**

| WT/MT | $APA(mV)$ | $MUV(V/s)$ | $RMP(mV)$ | $\mathrm{APD}_{90}(ms)$ | $ERP(ms)$ |
| --- | --- | --- | --- | --- | --- |
| $\mathrm{WT}$ | 99.2 | 192.0 | -75.6 | 247.4 | 305 |
| A39V (exon 8) | 101.0 | 205.8 | -78.0 | 51.9 | 90 |
| A39V | 101.1 | 206.6 | -78.2 | 45.6 | 83 |
| $G490R$ | 101.1 | 206.9 | -78.3 | 41.7 | 78 |
