# Supplementary material for: Biphasic effects on human atrial arrhythmogenicity of L-type calcium channel mutations associated with a Brugada/Short QT overlap syndrome - insights from a multiscale simulation study
Source: PLoS Comput Biol. 2025 Nov 19;21(11):e1013616. doi: 10.1371/journal.pcbi.1013616 (PMC12629484; doi:10.1371/journal.pcbi.1013616)
Supplement: S6 Table — Lifespans of reentries and computed dominant frequency of time series, and spiral wave core movement area of re-entrant excitations in WT, CACNA1C A39V mutation and corresponding deficient ICaL conditions in the 2D model. (DOCX) [file pcbi.1013616.s024.docx]

**Table S6**

**Biphasic effects of on human atrial arrhythmogenicity of L-type calcium channel mutations associated with a Brugada/Short QT overlap syndrome - insights from a multiscale simulation study**

Yirong Xiang, Jules C. Hancox, Henggui Zhang

**Table S6: Two-dimensional simulation results for A39V corresponding deficient** $\mathbf{I}_{\mathbf{CaL}}$ $I_{\mathrm{CaL}}$ **conditions.**

|  | WT | f=0.2 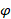 | f=0.4 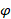 | f=0.5 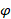 | f=0.6 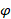 | f=0.8 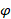 | f=1 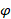 |
| --- | --- | --- | --- | --- | --- | --- | --- |
| LS (ms) | 5400 | 6900 | 6000 | 8000 | 8000 | 8000 | 8000 |
| $DF (Hz)$ | 3.8 | 3.9 | 4.3 | 4.6 | 5.1 | 6.1 | 6.8 |
| Tip Meander Area ($\mathrm{cm}^{2}$ $\mathrm{cm}^{2}$) | 14.9 | 18.5 | 43.4 | 42.4 | 11.6 | 7.2 | 5.0 |

Lifespans of reentries and computed dominant frequency (DF) of time series, and spiral wave core movement area of re-entrant excitations in WT, CACNA1C A39V mutation and corresponding deficient $I_{\mathrm{CaL}}$ $I_{\mathrm{CaL}}$ conditions in the 2D model.
