# Supplementary material for: Biphasic effects on human atrial arrhythmogenicity of L-type calcium channel mutations associated with a Brugada/Short QT overlap syndrome - insights from a multiscale simulation study
Source: PLoS Comput Biol. 2025 Nov 19;21(11):e1013616. doi: 10.1371/journal.pcbi.1013616 (PMC12629484; doi:10.1371/journal.pcbi.1013616)

**Table S7**

**Biphasic effects of on human atrial arrhythmogenicity of L-type calcium channel mutations associated with a Brugada/Short QT overlap syndrome - insights from a multiscale simulation study**

Yirong Xiang, Jules C. Hancox, Henggui Zhang

**Table S7: Three-dimensional simulation results for A39V corresponding deficient** $\mathbf{I}_{\mathbf{CaL}}$ $I_{\mathrm{CaL}}$ **conditions.**

| WT/MT  metrics | WT | f=0.2 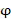 | f=0.4 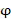 | f=0.5 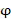 | f=0.6 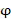 | f=0.8 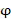 | f=1 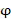 (A39V) |
| --- | --- | --- | --- | --- | --- | --- | --- |
| $Lifespan (ms)$ | 5000 | 4500 | 1100 | 800 | 5000 | 5000 | 5000 |
| $DF (HZ)$ | 3.7 | 3.8 | 3.9 | / | 4.6 | 5.5 | 6.0 |

Lifespan and computed dominant frequency (DF) of scroll waves in WT, CACNA1C A39V mutation, and corresponding deficient $I_{\mathrm{CaL}}$ conditions within the 3D model.


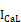

Supplement: S7 Table — Lifespan and computed dominant frequency of scroll waves in WT, CACNA1C A39V mutation, and corresponding deficient ICaL conditions within the 3D model. (DOCX) [file pcbi.1013616.s025.docx]
